# Slowly Expanding Stable Dust Spacetimes

**DOI:** 10.1007/s00205-024-02030-7

**Published:** 2024-09-13

**Authors:** David Fajman, Maximilian Ofner, Zoe Wyatt

**Affiliations:** 1 Faculty of Physics, University of Vienna, Boltzmanngasse 5, 1090 Vienna, Austria; 2 Department of Pure Mathematics and Mathematical Statistics, Wilberforce Road, Cambridge, CB3 0WB UK

**Keywords:** 35Q75, 83C05, 35B35

## Abstract

We establish the future nonlinear stability of a large class of FLRW models as solutions to the Einstein-Dust system. We consider the case of a vanishing cosmological constant, which, in particular implies that the expansion rate of the respective models is linear, i.e. has zero acceleration. The resulting spacetimes are future globally regular. These solutions constitute the first generic class of future regular Einstein-Dust spacetimes not undergoing accelerated expansion and are thereby the slowest expanding generic family of future complete Einstein-Dust spacetimes currently known.

## Introduction

### General Relativistic Hydrodynamics

The Einstein-relativistic Euler system (EES)

1.1 $$\begin{aligned} \begin{aligned} R_{\mu \nu }-\frac{1}{2} R g_{\mu \nu }&=T_{\mu \nu }\\ \nabla ^{\mu }T_{\mu \nu }&=0\\ T_{\mu \nu }&=(\rho +p)u_{\mu }u_{\nu }+p g_{\mu \nu }\end{aligned} \end{aligned}$$

describes the dynamical evolution of a four-dimensional spacetime $(\mathcal {M}, g)$ containing a relativistic perfect fluid with pressure *p*, energy density $\rho$ and 4-velocity vector $u^\mu$. Perfect fluids compatible with relativity were one of the earliest matter models considered in general relativity [7, 22] and have been extensively studied in the context of general relativistic hydrodynamics with numerous applications ranging from astrophysics to cosmological evolution (see e.g. [10, 34]).

The Eq. (1.1) are supplemented by specifying an equation of state which relates the energy density and pressure $p=f(\rho )$. Different choices of the function *f* encode different behaviour of the fluid. We focus in the following on the class of linear, barotropic equations of state, $p=c_S^2 \rho$, where the constant $c_S$ denotes the speed of sound of the fluid with $0\le c_S\le 1$. This equation of state contains well-known fluid models: for $c_S=0$, i.e. $p=0$, (1.1) reduces to the *Einstein-Dust* system, the case $c_S=1/\sqrt{3}$ is the *Einstein-radiation fluid* system and $c_S=1$ is the *Einstein-stiff fluid* system. The main result of the present paper can be roughly stated as follows:

#### Theorem 1.1

All four-dimensional FLRW spacetime models with compact spatial slices and negative spatial Einstein geometry are future stable solutions of the Einstein-Dust system.

To date, all known future stability results establishing the global existence, regularity and completeness of solutions to (1.1) concern the regime of accelerated expansion. In such a setting, the fast decay rates of perturbations induced by the expansion have a strong regularization effect on the fluid. For slower expansion rates this effect becomes weaker and global regularity of solutions is less likely to hold.

Theorem 1.1 establishes the **first nonlinear future stability result for a coupled Einstein-relativistic Euler system in the absence of accelerated expansion** and thereby initiates the study of the EES in the regime of non-accelerated expansion. Such a regime is also relevant in cosmology. The epoch in the early universe, shortly after a hypothetical inflationary phase, is expected to not initially have exhibited accelerated expansion. It is this epoch, which is not covered by previous results on the EES, which we intend to make accessible by the research initiated in the present paper.

### Background and Previous Results

For the sake of the following presentation we consider four-dimensional FLRW spacetimes of the form

1.2 $$\begin{aligned} \left( (0,\infty )\times M,-\text {d}t_c^2+a(t_c)^2\cdot \gamma \right) , \end{aligned}$$

where $(M,\gamma )$ is a complete Riemannian manifold. We remind the reader that in the standard FLRW-models the spatial slices appearing in (1.2) have constant sectional curvature $k_M\in \{ -1,0,1\}$ and so are taken to be one of $\mathbb {R}^3, \mathbb {S}^3, \mathbb {H}^3$ or quotients thereof (eg, $\mathbb {T}^3$). We distinguish three classes of scale factors: $\ddot{a}(t_c)>0$ are referred to as *accelerated expansion*, $\ddot{a}(t_c)<0$ as *deccelerated expansion* and $\ddot{a}(t_c)=0$ as *linear expansion*. We introduce the notion of *power law inflation*, where $a(t_c)=(t_c)^{p}$ for $p>0$. Finally, we recall that a cosmological constant can be included by adding $+ \Lambda g_{\mu \nu }$ to the LHS of (1.1). In the following discussion only $\Lambda \ge 0$ is relevant.

#### Shock Formation

Relavistic and non-relativistic fluids are well-known to form shocks in finite time. This was first observed in the general relativistic context by Oppenheimer and Snyder when they investigated the collapse of spherically symmetric clouds of dust [22]. In the terminology of current stability analysis this constitutes the instability of Minkowski spacetime as a particular solution to the Einstein-Dust system. Note that a part of Minkowski spacetime corresponds to $M = \mathbb {R}^3, \gamma =\delta , a(t_c) = 1$ in (1.2).

More recently, Christodoulou’s monograph [9] demonstrated that under a very general equation of state, the constant solutions to the relativistic Euler equations on a fixed background Minkowski space are unstable, i.e. even without gravitational backreaction, fluids form shocks from arbitrarily small initial inhomogeneities in finite time. This suggests that Minkowski spacetime is unstable as a solution to the EES for a large class of equations of state. Note also that Christodoulou’s monograph gave a detailed description of the nature of the fluid shock formation, thus providing a major extension beyond work of Sideris [29] on the non-relativistic Euler equations.

#### $\Lambda$-induced Accelerated Expansion and Stabilisation of Fluids

As is clear from the previous paragraphs, a powerful dispersive mechanism is required to regularise fluids and to prevent finite-time shock formation. The prime example of such a mechanism comes from cosmological models exhibiting exponential expansion. Heuristically speaking, a cosmological constant $\Lambda >0$ generates expansion of the form $a(t_c) \sim e^{Ht_c}$ where $H = \sqrt{\Lambda /3}$, for *all* cases of the sectional curvature $k_M$. The cosmological constant creates damping terms in the equations of motion for the fluid, which dilutes the fluid and causes fluid lines to ‘stretch apart’, thus preventing shock formation.

This effect was first observed by Brauer, Rendall and Reula [6] who studied Newtonian cosmological models with $\Lambda >0$ and a perfect fluid (albeit for a slightly different equation of state). They found that the regularising effect from the exponential expansion was strong enough to prevent shock formation for small inhomogeneities of initially uniformly quiet fluid states. See also the late-time asymptotics work by Reula [25] and Rendall [23].

Moving to the fully coupled Einstein-relativistic Euler system, there has been much research concerning spacetimes undergoing exponential expansion. The first result is by Rodnianski and Speck [28], who proved future stability to irrotational perturbations of uniformly quiet fluids with $0<c_S<1/\sqrt{3}$ on FLRW-spacetimes with underlying spatial manifold $M=\mathbb {T}^3$. The irrotational restriction was later removed by Speck in [30]. An alternative proof of future stability for these FLRW-background solutions was later given by Oliynyk [20], whose Fuchsian techniques were able to uniformly cover the cases $0<c_S\le 1/\sqrt{3}$.

Moving to the case of dust $c_S = 0$, stability for FLRW-spacetimes with underlying spatial manifold $M=\mathbb {T}^3$ was given by Hadžić and Speck [16], while more general spatial manifolds were considered by Friedrich [15] using his conformal method. Indeed the work by Lübbe and Valiente-Kroon [19] treated the radiation case with $c_S = 1/3$ using an extension of Friedrich’s conformal method. Finally, we note that very recent work of Oliynyk [21] has established stability for ultra-radiation fluids $1/\sqrt{3}<c_S\le 1/\sqrt{2}$ on a fixed, exponentially expanding spacetime.

#### Alternative Mechanisms for Accelerated Expansion

A cosmological constant is not the only known mechanism for generating solutions to Einstein’s equations with accelerated expansion. In work that predates the references of Sect. 1.2.2, Ringström [26] considered the future global stability of a large class of solutions to the Einstein-nonlinear-scalar field system with a scalar field potential $V(\Phi )$ that satisfied $V(0)>0, V'(0)=0, V''(0)>0$. Roughly speaking *V*(0) emulates the cosmological constant $\Lambda$ and so these spacetimes undergo accelerated expansion. Ringström [27] later considered alternative potentials $V(\Phi )$ which relaxed the rate of spacetime expansion to the class of accelerated power law inflation, which in our terminology corresponds to $p>1$.

Note that in Ringström’s papers the global spatial topology becomes irrelevant for the long time behaviour of cosmological spacetimes in the small data regime. This is in sharp contrast to the Einstein *vacuum* equations where the spatial topology does affect the long-time behaviour. In this case, only the Milne geometry with a negative spatial curvature yields future eternally expanding cosmological models with precisely linear expansion rate.

Finally we note that the Chaplygin equation of state, which describes a fluid with negative pressure, can also generate sufficient spatial expansion to ensure future stability results for the coupled EES system [18].

#### Critical Expansion Rates

Interpolating between Minkowski space (which can be considered as a cosmological spacetime with non-compact slices and no expansion) and exponentially expanding spacetimes, it is clear that there must be a transition between shock formation and stability. To investigate the expansion rate for which this transition occurs, and how it depends on the equation of state, it is useful to study the stabilisation of fluids on fixed Lorentzian geometries obeying power-law inflation. We consider $M = \mathbb {T}^3$ with $a(t_c) = (t_c)^p$ for $p>0$. The following table summarises some of the main results concerning linear equation of states $p= c_S^2 \rho$ from [13, 31], (see also [33]):

**Table:** 

Case	Power-law rate	Range of $c_S$	Behaviour	References
No. 1	$p>1$	$0<c_S<1/\sqrt{3}$	Stable	[31]
No. 2	$p=1$	$c_S = 1/\sqrt{3}$	Shocks	[31]
No. 3	$p=1$	$0<c_S<1/\sqrt{3}$	Stable (irrot.)	[13]
No. 4	$p>\frac{1}{2}$	$c_S = 0$	Stable	[31]

In combination, these results indicate that in spacetimes undergoing power-law inflation whether shocks form from small data depends on the equation of state and, in particular in the linear case, on the speed of sound. Indeed the literature suggests that *slower speeds of sound reduce the tendency of shock formation*. For the particular case of dust ($c_S=0$), case No. 4 shows that shocks are avoided even in deccelerating spacetimes with scale factors $a(t)=t^{1/2+\delta }$ for $\delta >0$.

### Main Results

In the present paper we consider the Einstein-Dust system in the regime of linear expansion. For the linearly expanding case, Cases No. 2 and 3 above show that even in the absence of backreaction the speed of sound determines whether shocks form or not. We prove that for the case of dust ($c_S=0$) shock formation does not occur under the full gravity-fluid dynamics.

Our background geometry is that of the Milne model, which generalises the $k_M = -1$ FLRW vacuum spacetimes. Let $(M, \gamma )$ be a closed, connected, orientable three-dimensional manifold admitting a Riemannian Einstein metric $\gamma$ with negative Einstein constant. After rescaling, we suppose that

$$\begin{aligned} \text {Ric}[\gamma ] = -\frac{2}{9} \gamma . \end{aligned}$$

The generalised Milne spacetime is the Lorentz cone spacetime $\mathcal {M} = (0,\infty ) \times M$ with metric

$$\begin{aligned} g_M :=-\text {d}t_c^2 + \frac{t_c^2}{9}\gamma _{ab} \text {d}x^a \text {d}x^b. \end{aligned}$$

The spacetime $(\mathcal {M}, g_M)$ is globally hyperbolic and a solution to the four-dimensional vacuum Einstein equations. We formulate the main theorem using terminology introduced in Sect. 3.1. We let $\mathscr {B}_\varepsilon ^{j,k,l,m}\big (\tfrac{t_0^2}{9}\gamma ,-\tfrac{t_0}{9} \gamma ,0,0\big )$ denote the ball of radius $\varepsilon$ in the space $H^j\times H^k\times H^l\times H^m$ centred at $\big (\tfrac{t_0^2}{9}\gamma ,-\tfrac{t_0}{9} \gamma ,0,0\big )$. Our main theorem is

#### Theorem 1.2

Let $(\mathcal {M},g_M)$ be as above. Let $\varepsilon >0$ and $(g_0,k_0,\rho _0,u_0)$ be initial data for the Einstein-Dust system at $t_c=t_0$ such that

$$\begin{aligned} (g_0,k_0,\rho _0,u_0)\in \mathscr {B}_\varepsilon ^{6,5,4,5}\left( \frac{t_0^2}{9}\gamma ,-\frac{t_0}{9} \gamma ,0,0\right) . \end{aligned}$$

Then, for $\varepsilon$ sufficiently small the corresponding future development under the Einstein-Dust system is future complete and admits a CMC foliation labelled by $\tau \in [\tau _0, 0)$ such that the induced metric and second fundamental form on constant CMC slices converge as

$$\begin{aligned} (\tau ^2 g,\tau k)\rightarrow \left( \gamma ,\frac{1}{3} \gamma \right) \text{ as } \tau \nearrow 0 \quad \text {i.e. as } t_c\nearrow \infty . \end{aligned}$$

If the initial energy density of the dust field is non-negative, $\rho _0\ge 0$, then it remains so throughout the evolution.

#### Remark 1.3

The Milne model is known to be a stable solution to the Einstein vacuum equations [3], the Einstein massive-Vlasov equations [1], the coupled Einstein-Maxwell-scalar field system arising from a Kaluza-Klein reduction [5], and the Einstein Klein-Gordon equations [14, 32].

#### Remark 1.4

Negative spatial curvature is crucial as spherical or toroidal spatial topologies would lead to recollapsing or slowly expanding matter dominated solutions, respectively. The asymptotic behaviour of the solutions in the theorem coincide with the corresponding vacuum solutions.

#### Structure and Key Novelties in the Proof

The proof of Theorem 1.2 consists of three major parts: (*i*) energy estimates for the perturbation of the spacetime geometry with sources given by the dust variables, (*ii*) energy estimates for the dust variables in the perturbed spacetime geometry and (*iii*) a bootstrap argument based on both sets of energy estimates establishing global existence and asymptotic behaviour. This rough approach is standard in the literature on the Milne stability problem (see e.g. [1, 3, 5]), however, for the Einstein–dust system there are crucial difficulties caused by a regularity problem inherent to the dust equations, which turns out to affect all parts of the argument. We outline the difficulties and how these are overcome in the following.

To control the perturbed spacetime geometry throughout the evolution we use a CMC time-foliation in combination with a spatial-harmonic gauge [2]. The existence of such a foliation for small perturbations of negative Einstein spaces is non-trivial but standard [12]. The Einstein equations then take the form of an elliptic-hyperbolic system (see (2.7)) where the lapse and shift are determined by elliptic PDEs with sources given in terms of metric, second fundamental form and the dust variables. This elliptic system provides Sobolev estimates for the lapse and shift.

The core idea to establish decay for the geometric variables in previous works on Milne stability is a corrected energy (${E^g}_k$ in Definition 3.8) based on the modified Einstein-operator ($\mathcal {L}_{g, \gamma }$ in Definition 3.2) of the spatial Einstein geometry [1, 3]. For the Einstein–Dust system we must deviate from this standard approach due to a regularity issue from the dust model, which in turn affects all parts of the proof.

When expanded, the equations of motion for the dust variables take a form where the source term of the evolution equation for the energy density contains the spatial divergence of the fluid velocity (see (2.7d)). Consequently, the fluid energy density can be controlled only in one order of regularity below the order of regularity of the fluid velocity. From the perspective of the Einstein equations this is very problematic as both components of the dust, energy density and fluid velocity, appear at the same order of regularity as source terms of the Einstein equations. As such, they are required to be controlled in suitable Sobolev spaces at the same order as the second fundamental form. Due to the required high regularity of the fluid velocity discussed previously, the velocity then needs to be controlled one order above the second fundamental form. However, the equation of motion for the fluid velocity requires the second fundamental form at the same order of regularity as the velocity itself (see (2.7d)). This apparent inconsistency prevents one from establishing a standard and straightforward regularity hierarchy to analyse the fully coupled nonlinear system.

An approach to circumvent this issue has been introduced by Hadžić and Speck in [16] and is modified in the present paper. The central idea is to use a fluid derivative $\partial _{\textbf{u}}\sim u^\alpha \nabla _\alpha [g_M]$ as a differential operator in the energies for the perturbations of the metric and second fundemental form (${E^g_{\partial _{\textbf{u}},N-1}}$ in Definition 3.11). At highest order of regularity, say *N*, where the loss of derivatives prevents the closure of the system of estimates, the Einstein equations are commuted with $N-1$ spatial derivatives and one fluid derivative. When this derivative acts on the dust source terms in the Einstein equations, in the subsequent calculations for the energy estimates, the equations of motion of the dust variables are used as constraint equations replacing $\partial _{\textbf{u}}\rho$ and $\partial _{\textbf{u}}u^j$. In this way, no derivatives are lost and the corresponding auxiliary energies ${E^g_{\partial _{\textbf{u}},N-1}}$ for the geometric variables can be estimated in terms of dust variables of one order of regularity below the expected one.

In a follow-up step, we need to show that the auxiliary geometric energies ${E^g_{\partial _{\textbf{u}},N-1}}$ in fact control the actual top-order regularity norms of the geometric variables. This is achieved by rewriting the wave-type evolution equation for the metric and second fundamental in terms of an elliptic part and certain mixed spatial and fluid derivative operators (see Proposition 6.3). Consequently, the auxiliary energies of the first step provide top-order estimates on the geometric variables (see Corollary 6.4) and an overall strategy to close the estimates.

Two final major regularity issues arise when proving energy estimates for the auxiliary energies ${E^g_{\partial _{\textbf{u}},N-1}}$ however. We end up needing to estimate one fluid derivative and a critical number of spatial derivatives on certain terms involving the lapse and shift, and we cannot commute the $\partial _{\textbf{u}}$ operator past the spatial derivatives without exceeding the assumed regularity of the fluid spatial velocity.

To circumvent this problem, we only commute past some of the derivatives and instead derive two auxiliary estimates using the elliptic Eq. (2.7b) for the lapse and shift. In the estimate on the lapse term (see Proposition 7.3) we crucially use the equations of motion of the dust variables to replace a certain matter term $\partial _{\textbf{u}}\eta$ as a constraint, thus avoiding derivative loss. The estimate for the shift term (see Proposition 7.6) proceeds differently, relying on a *remarkable combination* of commutator estimates, the Bianchi identity and the Einstein equations in the CMCSH gauge.

#### Final Remarks

In the regime of non-accelerated expansion, the work [6] indicates that the backreaction between the fluid and the geometry cannot be ignored. The authors consider the case of dust with a Newtonian backreaction, finding that shocks form for arbitrarily small initial data in the regime where the homogeneous background spacetime, which is perturbed, expands like $a(t)=t^{2/3}$. This contrasts *noteably* with case No. 5 above which does not include backreaction. While [6] concerns only Newtonian dynamics, it is nevertheless a fair indication that the fully coupled dynamics under the Einstein-fluid system will likely lead to the formation of shocks. Continuing this line of reasoning, we note that although the work [13] also treated linear expansion, the full coupling between gravity and fluid makes our present work highly nontrivial. Indeed the issues highlighted on the previous Sect. 1.3.1 are indicative of the substantial technical difficulties that arise in the fully coupled EES.

Finally, it is interesting to recall that Sachs and Wolfe derived a linear *in*stability result for the Einstein-Dust equations with $\Lambda = 0$, however their metric had underlying spatial manifold $M = \mathbb {R}^3$ [35]. The fluid plays a major dynamical role in these flat FLRW models. Nevertheless one gleans the importance of the negatively curved spatial slices appearing in our nonlinear stability result.

### Outline of the Paper

In Sect. 2 we introduce the system of equations and perform a natural rescaling of the variables. In Sect. 3 we introduce function spaces and energy functionals controlling the metric perturbation and shear tensor.

The main theorem is proved using continuous induction. In Sect. 4 we discuss the local existence theory and initiate the bootstrap argument. The remainder of the paper, beginning with Sect. 5, treats the individual estimates necessary to close the bootstrap argument. Section 5 gathers various auxiliary estimates, which are used in later sections. Among those are estimates on the source terms of the evolution equations, estimates on the dust-derivative acting on various quantities, commutators of the dust derivative and other operators and estimates on high derivatives combining the dust-derivative and other operators.

Section 6 derives the elliptic estimate for the Einstein operator and the evolution equations, which is crucial to turn estimates in terms of the dust derivatives into those in terms of standard energies. These estimates are then given subsequently. In Sect. 7 we provide the estimates on lapse function and shift vector field. A crucial set of lapse and shift estimates on highest order of regularity, involving also the dust derivative, are given here too. In Sect. 8 we derive the central top-order energy estimate for the auxiliary energy controlling the geometric perturbations. In Sect. 9 we derive the estimates for the dust variables and in Sect. 10 we close the bootstrap.

## Equations of Motion

### The Einstein–Dust System

The Einstein–relativistic Euler system reads

2.1 $$\begin{aligned} \begin{aligned} R_{\mu \nu }[\bar{g}]-\frac{1}{2}R[\bar{g}]\bar{g}_{\mu \nu }&=2\tilde{T}_{\mu \nu },\\ {\bar{\nabla }}_{\mu }\tilde{T}^{\mu \nu }&=0,\\ \tilde{T}^{\mu \nu }&=({\tilde{\rho }}+\tilde{P}) \tilde{u}^{\mu }\tilde{u}^\nu +\tilde{P}\bar{g}^{\mu \nu } ,\end{aligned} \end{aligned}$$

where we set $c=1$ and $4\pi G = 1$. We use ${\bar{\nabla }}$ to denote the Levi-Civita connection of the physical metric $\bar{g}$. The four-velocity of the fluid $\tilde{u}^\mu$ is a future-directed timelike vectorfield normalised by

2.2 $$\begin{aligned} \bar{g}_{\mu \nu } \tilde{u}^\mu \tilde{u}^\nu =-1 \,. \end{aligned}$$

We assume a linear, barytropic fluid equation of state $\tilde{P}= c_S^2 {\tilde{\rho }}$ where $c_S \ge 0$ is a constant, and $\tilde{P}\ge 0$ and ${\tilde{\rho }}\ge 0$ denote the pressure and energy density respectively. In the present paper, we **restrict ourselves to dust**, which means we set

$$\begin{aligned} c_S^2 :=0 \,. \end{aligned}$$

The fluid equations in (2.1) can equivalently (for ${\tilde{\rho }}>0$) be written as

2.3 $$\begin{aligned} \begin{aligned} \tilde{u}^{\alpha }{\bar{\nabla }}_{\alpha }\ln {\tilde{\rho }}+{\bar{\nabla }}_\alpha \tilde{u}^{\alpha }&=0\,,\qquad \tilde{u}^{\alpha }{\bar{\nabla }}_{\alpha } \tilde{u}^{\mu }=0\,.\end{aligned} \end{aligned}$$

The system (2.3) is overdetermined in the sense that $\tilde{u}^0$ can be determined from the other fluid velocity components via (2.2).

We will study the Einstein-Dust equations using the following ADM ansatz for the metric

2.4 $$\begin{aligned} \bar{g}= -\tilde{N}^2 \text {d}t^2 +\tilde{g}_{ab}(\text {d}x^a+\tilde{X}^a\text {d}t)(\text {d}x^b + \tilde{X}^b \text {d}t). \end{aligned}$$

Note that $\bar{g}_{ab}=\tilde{g}_{ab}$ but in general $\bar{g}^{ab}\ne \tilde{g}^{ab}$. On *t*=constant slices, we let $\tau$ be the trace of the second fundamental form ${\tilde{k}}$ with respect to ${\tilde{g}}$ and define $\Sigma$ to be the trace-free part of ${\tilde{k}}$; that is,

$$\begin{aligned} \begin{aligned} \tau&:=\text {tr}_{{\tilde{g}}} {\tilde{k}} = \tilde{g}^{ab} \tilde{k}_{ab},\qquad {\tilde{k}}:={\tilde{\Sigma }}+\tfrac{1}{3} \tau {\tilde{g}}.\end{aligned} \end{aligned}$$

We use Roman letters (*a*, *b*, *i*, *j*...) to denote spatial indices. Let $\nabla$ denote the Levi-Civita connection of the spatial metric $\tilde{g}$. Using (2.4) the Christoffel symbols of the 4-metric $\bar{g}$ become (see e.g. [24])

$$\begin{aligned} \begin{aligned} ^{(4)}\tilde{\Gamma }^{0}_{0 0}&=\tilde{N}^{-1}( \partial _t \tilde{N}+ \tilde{X}^a\nabla _a\tilde{N}- \tilde{k}_{ab}\tilde{X}^a\tilde{X}^b ),\qquad&^{(4)}\tilde{\Gamma }^{0}_{a b}&=-\tilde{N}^{-1}\tilde{k}_{ab}, \\ ^{(4)}\tilde{\Gamma }^{0}_{a 0}&=\tilde{N}^{-1}(\nabla _a\tilde{N}- \tilde{k}_{ab}\tilde{X}^b), \quad&^{(4)}\tilde{\Gamma }^{a}_{b c}&=\Gamma ^a_{bc}[\tilde{g}]+\tilde{N}^{-1}{\tilde{k}}_{bc}\tilde{X}^a,\\ ^{(4)}\tilde{\Gamma }^{a}_{0 b}&=-\tilde{N}{\tilde{k}}^a_b+\nabla _b\tilde{X}^a-\tilde{N}^{-1}\tilde{X}^a\nabla _b\tilde{N}\\&\quad +\tilde{N}^{-1}{\tilde{k}}_{bc}\tilde{X}^c\tilde{X}^a,\\ ^{(4)}\tilde{\Gamma }^{a}_{0 0}&=\partial _t\tilde{X}^a+\tilde{X}^b\nabla _b\tilde{X}^a-2\tilde{N}{\tilde{k}}_c^a\tilde{X}^c+\tilde{N}\nabla ^a\tilde{N}\\&\quad -\tilde{N}^{-1}(\partial _t\tilde{N}+\tilde{X}^b\nabla _b\tilde{N}-\tilde{k}_{bc}\tilde{X}^b\tilde{X}^c)\tilde{X}^a. \end{aligned} \end{aligned}$$

Noting the above, the fluid Eq. (2.3) reduce to

$$\begin{aligned} \tilde{u}^{\alpha }\partial _\alpha \ln {\tilde{\rho }}+\partial _\alpha \tilde{u}^{\alpha }+^{(4)}\tilde{\Gamma }^{\alpha }_{\alpha \nu } \tilde{u}^\nu&=0,\qquad \tilde{u}^{\alpha }\partial _{\alpha } \tilde{u}^{\mu }+\tilde{u}^\alpha {^{(4)}\tilde{\Gamma }^{\mu }_{\alpha \nu } \tilde{u}^\nu } =0. \end{aligned}$$

### The Rescaled Einstein–Dust System in CMCSH Gauge

Following the work of Andersson and Moncrief [2, 3], we hereon impose the CMCSH gauge which foliates by surfaces of constant mean curvature, taking advantage of the fact that on the Milne background $\tau = \tilde{g}^{ab}\tilde{k}_{ab} = -3/t_c$.

#### Definition 2.1

(CMCSH gauge)

$$\begin{aligned} \begin{aligned} t=\tau ,\qquad H^a:=\tilde{g}^{cb}(\Gamma [\tilde{g}]^{a}_{cb}-\Gamma [\gamma ]^a_{cb})&=0. \end{aligned} \end{aligned}$$

We next rescale our variables with respect to the mean curvature $\tau$.

#### Definition 2.2

(Rescaled variables $(g_{ab}, N, X^a, \Sigma _{ab}, u^a, u^0, \rho , \widehat{N}, \hat{u}^0)$ and logarithmic time *T*) The rescaled geometric variables are defined as

2.5a $$\begin{aligned} \begin{array}{rlrl} g_{ab}& :=\tau ^2\tilde{g}_{ab},& g^{ab}:=& (\tau ^2)^{-1}\tilde{g}^{ab},\\ N& :=\tau ^2\tilde{N}, & X^a:=& \tau \tilde{X}^a,\\ \Sigma _{ab}& :=\tau {\tilde{\Sigma }}_{ab}. \end{array} \end{aligned}$$

Note $\tilde{X}_a = \tilde{g}_{ab}\tilde{X}^b = \tau ^{-3} X_a$. Let $\tilde{u}^0:=\tilde{u}^\tau$. The rescaled matter variables are defined as

2.5b $$\begin{aligned} u^a:=\tau ^{-2}\tilde{u}^a , \qquad \rho :=|\tau |^{-3}{\tilde{\rho }}, \qquad u^0 :=\tau ^{-2} \tilde{u}^0 . \end{aligned}$$

Denote ${\widehat{N}}:=\frac{N}{3}-1$ and $\hat{u}^0:=u^0-1/3$. Finally we define the logarithmic time

$$\begin{aligned} T:=-\ln (\tau /(e\tau _0)), \end{aligned}$$

which satisfies $$\partial _T=-\tau \partial _\tau $$.

The above definition means we have the following ranges $\tau _0 \le \tau \nearrow 0$ and $1 \le T \nearrow \infty$ where $\tau \nearrow 0$ corresponds to the direction of cosmological expansion (i.e. $t_c \nearrow \infty$).

#### Lemma 2.3

The normalisation condition (2.2) implies

$$\begin{aligned} \begin{aligned} u^0&= \frac{1}{(N^2 - X_a X^a)}\Big (\tau X_a u^a+ \Big [\tau ^{2}(X_a u^a)^2 + (N^2 - X_aX^a)(\tau ^{2}g_{ab} u^a u^b+1)\Big ]^{1/2}\Big ) .\end{aligned} \end{aligned}$$

#### Proof

Using (2.2) we have

$$\begin{aligned} 0 = (-\tilde{N}^2 + \tilde{X}_a\tilde{X}^a)(\tilde{u}^0)^2 + 2\tilde{X}_a \tilde{u}^a \tilde{u}^0 + \tilde{g}_{ab}\tilde{u}^a\tilde{u}^b+1. \end{aligned}$$

This is a quadratic equation in $\tilde{u}^0$. The roots are

$$\begin{aligned} \tilde{u}^0 = \frac{1}{2(-\tilde{N}^2 + \tilde{X}_a\tilde{X}^a)}\Big ( -2\tilde{X}_a \tilde{u}^a\pm \Big [4(\tilde{X}_a \tilde{u}^a)^2 - 4(-\tilde{N}^2 + \tilde{X}_a\tilde{X}^a)(\tilde{g}_{ab}\tilde{u}^a\tilde{u}^b+1)\Big ]^{1/2}\Big ) . \end{aligned}$$

Applying the rescalings from Definition 2.2 we find

$$\begin{aligned} \tilde{u}^0 = \frac{\tau ^4}{(N^2 - X_a X^a)}\Big (\! \tau ^{-1}X_a u^a\mp \Big [\!\tau ^{-2}(X_a u^a)^2 + (N^2 - X_aX^a)(\tau ^{-2}g_{ab} u^a u^b+\tau ^{-4})\!\Big ]^{1/2}\!\Big ) . \end{aligned}$$

Hence, we introduce the rescaled quantity $u^0 = \tau ^{-2} \tilde{u}^0$ for the larger root. $\square$

Let $\nabla , \hat{\nabla }$ denote the Levi-Civita connection of the Riemannian metrics $g, \gamma$ respectively. The Christoffel symbols of $\bar{g}$ now become (see e.g. [1])

$$\begin{aligned}\begin{aligned} ^{(4)}\tilde{\Gamma }^{a}_{b c}&=\Gamma ^{a}_{b c}[g]-\Gamma _{bc}X^a,\quad&^{(4)}\tilde{\Gamma }^{a}_{0 0}&=\tau ^{-2}\Gamma ^a,\\ ^{(4)}\tilde{\Gamma }^{a}_{0 b}&=\tau ^{-1}\left( -\delta _b^a+\Gamma _b^a\right) ,\quad&^{(4)}\tilde{\Gamma }^{0}_{0 0}&=\tau ^{-1}(-2+\Gamma _R),\\ ^{(4)}\tilde{\Gamma }^{0}_{a b}&=\tau \Gamma _{ab},\quad&^{(4)}\tilde{\Gamma }^{0}_{0 a}&=\Gamma _a,\\ \end{aligned} \end{aligned}$$

where we have introduced the following rescaled geometric components.

#### Definition 2.4

(Rescaled Christoffel components $\Gamma ^a, \Gamma ^a_b, \Gamma _R, \Gamma _{ab}, \overset{\circ }{\Gamma } ^a$)

$$\begin{aligned} \begin{aligned} \Gamma ^a&:=-\partial _T X^a-X^a-2\widehat{N} X^a+X^b\nabla _b X^a-2 N \Sigma _c^a X^c+{N}{\nabla }^a{N}\\&\quad \,+ \Big (N^{-1}\partial _T N-N^{-1} X^b\nabla _b N+N^{-1}\left( \Sigma _{bc}+\tfrac{1}{3}g_{bc}\right) X^b X^c\Big )X^a, \\ \Gamma ^a_b&:=-N\Sigma ^a_b-\delta _b^a\widehat{N}+\nabla _b X^a- N^{-1} X^a\nabla _b N+ N^{-1}\left( \Sigma _{bc}+\tfrac{1}{3}g_{bc}\right) X^c X^a, \\ \Gamma _R&:=N^{-1}\big (-\partial _T N+X^a\nabla _a N-(\Sigma _{ab}+\tfrac{1}{3}g_{ab})X^aX^b\big ), \quad \overset{\circ }{\Gamma } ^a :=\Gamma ^a - N \nabla ^a N, \\ \Gamma _{ab}&=-N^{-1}(\Sigma _{ab}+\frac{1}{3}g_{ab}),\qquad \Gamma _a:=N^{-1}(\nabla _a N-(\Sigma _{ab}+\tfrac{1}{3}g_{ab})X^b).\end{aligned} \end{aligned}$$

#### Remark 2.5

(Background solutions) The rescaled background (B) Milne geometry written in CMCSH gauge is

$$\begin{aligned} (g_{ab}, \Sigma _{ab}, N, X^a)|_B\equiv (\gamma , 0, 3, 0). \end{aligned}$$

Furthermore,

$$\begin{aligned} (\Gamma ^a, \Gamma ^a_b, \Gamma _R, \Gamma _a) |_B \equiv 0, \quad \Gamma _{ab} |_B \equiv -\tfrac{1}{9} \gamma _{ab}. \end{aligned}$$

Let $\rho _0'>0$ be a constant. The background, uniformly quiet fluid solution in CMCSH gauge is

$$\begin{aligned} (u^0, u^i, \rho )|_B = (\tfrac{1}{3},0,\rho _0'); \end{aligned}$$

see also Appendix A.

We next evaluate certain energy momentum and matter source terms arising from the dust.

#### Definition 2.6

(Matter source terms $E, \jmath ^a, \eta , S_{ab}, \underline{T}^{ab}$)

$$\begin{aligned} E&:=\rho (u^0)^2 N^2,\quad \jmath ^a :=\rho N u^0 u^a, \\ \eta&:=E+ \rho g_{ab}(u^0 X^a+ \tau u^a)(u^0 X^b + \tau u^b), \\ S_{ab}&:=\rho ( u^0 X_a+ \tau u_a )(u^0 X_b+ \tau u_b) + \tfrac{1}{2}\rho g_{ab}, \quad \underline{T}^{ab} :=\rho u^a u^b. \end{aligned}$$

For further details on these definitions see Appendix 11.

#### Definition 2.7

(Matter source terms $F_{u^j}, F_{u^0}, F_\rho$)

2.6 $$\begin{aligned} \begin{aligned} F_{u^j}&:=\tau ^{-1}(u^0)^2\overset{\circ }{\Gamma } ^j + ({\Gamma ^{j}_{k l}}[g]-{\Gamma ^{j}_{k l}}[\gamma ])u^ku^l+2u^0u^i\Gamma ^j_i+\tau u^k u^i \Gamma _{ki}X^j, \\ F_{u^0}&:=(u^0)^2\Gamma _R+2\tau u^ju^0\Gamma _j + \tau ^2 u^k u^j \Gamma _{kj}, \\ F_\rho&:=\tau \nabla _i u^i + \Gamma ^i_i u^0 - \tau \Gamma _j u^j + \tau \Gamma _{ik}X^i u^k - \tau \frac{u^j}{u^0} \nabla _j u^0 -\tau ^2\frac{ u^k u^j}{u^0} \Gamma _{kj}.\end{aligned} \end{aligned}$$

### Equations of Motion

Bringing together all the previous notation, as well as using the general equations presented in [1], the equations of motion for the Einstein-Dust system in CMCSH gauge are the following. We have two constraint equations.

2.7a $$\begin{aligned} \begin{aligned} \text {R}(g)-|\Sigma |_g^2+\tfrac{2}{3}&= 4 \tau E, \\ \nabla ^a \Sigma _{ab}&= 2 \tau ^2 \jmath _b ,\end{aligned} \end{aligned}$$

and two elliptic equations for the lapse and shift variables,

2.7b $$\begin{aligned} \begin{aligned} (\Delta - \tfrac{1}{3})N&= N \left( |\Sigma |_g^2 - \tau \eta \right) -1, \\ \Delta X^a + \text {Ric}[g]^a _b X^b&= 2 \nabla _b N \Sigma ^{ba} - \nabla ^a \widehat{N}+ 2 N \tau ^2 \jmath ^a\\&\quad - (2N \Sigma ^{bc} - \nabla ^b X^c)(\Gamma [g]^a_{bc} - \Gamma [\gamma ]^a_{bc}).\end{aligned} \end{aligned}$$

We also have evolution equations for the induced metric and trace-free part of the second fundamental form

2.7c $$\begin{aligned} \begin{aligned} \partial _T g_{ab}&= 2N \Sigma _{ab} + 2\widehat{N}g_{ab} - \mathscr {L}_X g_{ab}, \\ \partial _T \Sigma _{ab}&= -2\Sigma _{ab} - N(\text {Ric}[g]_{ab} +\tfrac{2}{9}g_{ab} ) + \nabla _a \nabla _b N + 2N \Sigma _{ac} \Sigma ^c_b \\&\quad -\tfrac{1}{3} \widehat{N}g_{ab} - \widehat{N}\Sigma _{ab} - \mathscr {L}_X \Sigma _{ab} + N \tau S_{ab},\end{aligned} \end{aligned}$$

and, finally, evolution equations for the fluid components:

2.7d $$\begin{aligned} \begin{aligned} u^0\partial _Tu^j&= \tau u^a \nabla _a u^j + \tau ^{-1}(u^0)^2 N\nabla ^j N+F_{u^j}, \\ u^0\partial _T u^0&=\tau u^a \nabla _a u^0 + F_{u^0} , \\ u^0 \partial _T \rho&= \tau u^a \nabla _a \rho + \rho F_\rho .\end{aligned}\end{aligned}$$

Using notation from [2, 3] we introduce new variables which allow us to rewrite (2.7c).

#### Definition 2.8

(Perturbation variables *h*, *v*, *w* and geometric source terms $F_h, F_v$) Define the variables

$$\begin{aligned} h_{ab}:=g_{ab}-\gamma _{ab}, \quad v_{ab}:=6\Sigma _{ab}, \quad w :=N/3, \end{aligned}$$

and the geometric source terms

$$\begin{aligned}\begin{aligned} (F_h )_{ab}&:=2\widehat{N}g_{ab} + h_{ac}\hat{\nabla }_b X^c + h_{cb}\hat{\nabla }_a X^c , \\ (F_v )_{ab}&:=\nabla _a \nabla _b N + 2N \Sigma _{ac} \Sigma ^c_b-\tfrac{1}{3} \widehat{N}g_{ab} - \widehat{N}\Sigma _{ab}+ N \tau S_{ab}\\&\quad - v_{ac}\hat{\nabla }_b X^c - v_{cb}\hat{\nabla }_a X^c. \end{aligned}\end{aligned}$$

We start with the following identity from [2]:

$$\begin{aligned} \mathscr {L}_X g_{ab} = X^c \hat{\nabla }_c g_{ab} +g_{ac}\hat{\nabla }_b X^c + g_{cb}\hat{\nabla }_a X^c. \end{aligned}$$

Due to rigidity properties of negative Einstein manifolds in three spatial dimensions (see e.g. [3, §1.1]), we have $\partial _T \gamma = 0$. Thus the Eq. (2.7c) reduce to

2.8 $$\begin{aligned} \begin{aligned} \partial _T h_{ab}&= wv_{ab} - X^m \hat{\nabla }_m h_{ab} + F_h, \\ \partial _T v_{ab}&= -2v_{ab} - 9 w\mathcal {L}_{g, \gamma }h_{ab} - X^c\hat{\nabla }_c v_{ab} +6F_v.\end{aligned} \end{aligned}$$

In Sect. 8 we will also write the first equation in (2.8) as

2.9 $$\begin{aligned}  &   \partial _T h_{ab} = wv_{ab} + 2\widehat{N}g_{ab} - (\mathscr {L}_X g)_{ab} = wv_{ab} + 2\widehat{N}\nonumber \\  &   g_{ab} - g_{am}\nabla _b X^m - g_{bm} \nabla _a X^m. \end{aligned}$$

Hereon we use the differential Eq. (2.7b), (2.7d) and (2.8) to analyse the solutions to our Einstein-Dust system.

## Preliminary Definitions

In this section we present several preliminary definitions concerning Sobolev spaces, norms, elliptic estimates and energy functionals. All of this is standard except for Definitions 3.9 and 3.11 where we introduce the fluid derivative $\partial _{\textbf{u}}$ and then the energy functionals for the geometric variables involving this fluid derivative.

### Function Spaces and Norms

#### Definition 3.1

($L^2_{g,\gamma }$-inner product) Let $\mu _g = \sqrt{\det g}$ denote the volume element on (*M*, *g*), similarly for $\mu _\gamma$. Let *V*, *P* be (0, 2)-tensors on *M*. Define an inner product by

$$\begin{aligned} \langle V, P\rangle _\gamma :=V_{ij} P_{kl}\gamma ^{ik}\gamma ^{jl}, \end{aligned}$$

and define a mixed $L^2$-scalar product

$$\begin{aligned} (V, P)_{L^2(g, \gamma )} :=\int _M \langle V, P\rangle _\gamma \, \mu _g , \end{aligned}$$

with corresponding norm $\Vert V\Vert _{L^2_{g,\gamma }}^2 :=(V, V)_{L^2(g, \gamma )}.$

The following definition follows notation first introduced in [3].

#### Definition 3.2

(${{\,\textrm{Riem}\,}}{[}\gamma {]}\circ$ and $\mathcal {L}_{g, \gamma }$) Let *V* be a symmetric (0, 2)-tensor on *M*. Define the tensorial contraction

$$\begin{aligned} ({{\,\textrm{Riem}\,}}[\gamma ]\circ V)_{ij} :={{\,\textrm{Riem}\,}}[\gamma ]_{iajb}\gamma ^{aa'} \gamma ^{bb'}V_{a'b'} \,, \end{aligned}$$

where, following the convention of [2], the Riemann tensor is defined by $[\hat{\nabla }_a,\hat{\nabla }_i]V_b =(\hat{\nabla }_a\hat{\nabla }_i - \hat{\nabla }_i\hat{\nabla }_a) V_b:=-\text {Riem}[\gamma ]^c _{bai}V_c$. Define the following differential operators

$$\begin{aligned}\begin{aligned} \hat{\Delta }_{g, \gamma }V_{ij}&:=(\sqrt{\det g})^{-1}\hat{\nabla }_a\big (\sqrt{\det g}\cdot g^{ab} \hat{\nabla }_b V_{ij}\big ), \\ \mathcal {L}_{g, \gamma }V_{ij}&:=-\hat{\Delta }_{g, \gamma } V_{ij} - 2 ({{\,\textrm{Riem}\,}}[\gamma ]\circ V)_{ij} .\end{aligned}\end{aligned}$$

By using the gauge condition (2.1), the operator $\hat{\Delta }_{g, \gamma }$ can be rewritten as

$$\begin{aligned} \hat{\Delta }_{g, \gamma }V_{cd}= g^{ab}\hat{\nabla }_a \hat{\nabla }_b V_{cd}- H^a \hat{\nabla }_a V_{cd}. \end{aligned}$$

The operator $\mathcal {L}_{g, \gamma }$ is self-adjoint with respect to the mixed $L^2$-scalar product (see e.g. [2])

3.1 $$\begin{aligned} (\mathcal {L}_{g, \gamma }V, P)_{L^2(g, \gamma )} = (V, \mathcal {L}_{g, \gamma }P)_{L^2(g, \gamma )}. \end{aligned}$$

A self-adjoint elliptic operator on a compact manifold has a discrete spectrum of eigenvalues. Using eigenvalue estimates from [17], we are led to the following result:

#### Proposition 3.3

(Estimates on $\lambda _0$) Let $(M,\gamma )$ be a negative Einstein three-manifold with Einstein constant $k=-2/9$. Then the smallest eigenvalue of the operator $\mathcal {L}_{g, \gamma }$ satisfies $\lambda _0\ge 1/9$ and the operator also has trivial kernel $\ker (\mathcal {L}_{g, \gamma })=\{0\}$.

#### Definition 3.4

(Sobolev norms) Let $k \in \mathbb {Z}_{\ge 0}$. For $\kappa$ a Riemannian metric on *M*, *f* a function and *V* a (1, 1)-tensor, define

$$\begin{aligned} \begin{aligned} |\nabla ^k f|_\kappa ^2&:=\kappa ^{a_1 b_1} \cdots \kappa ^{a_k b_k} (\nabla _{a_1}\cdots \nabla _{a_k} f )\cdot (\nabla _{b_1}\cdots \nabla _{b_k} f ), \\ |\nabla ^k V|_\kappa ^2&:=\kappa _{ij} \kappa ^{kl} \kappa ^{a_1 b_1} \cdots \kappa ^{a_k b_k} (\nabla _{a_1}\cdots \nabla _{a_k} V^i _k)\cdot (\nabla _{b_1}\cdots \nabla _{b_k} V^j _l).\end{aligned} \end{aligned}$$

The obvious extension to (*p*, *q*)-tensors holds. We write

$$\begin{aligned} \begin{aligned} \Vert V \Vert _{H^k}&= \Big (\sum _{0\le \ell \le k}\int _M |\nabla ^\ell V|_g^2 \, \mu _g\Big )^{1/2}.\end{aligned} \end{aligned}$$

Note that under a global smallness assumption on $g-\gamma$ guaranteed by the bootstrap assumptions, we have the norm equivalence $\Vert \cdot \Vert _{L^2} \cong \Vert \cdot \Vert _{L^2_{g,\gamma }}$.

#### Remark 3.5

When we write $\Vert u \Vert _{H^{k}}$ we are denoting a sum *only* over the spatial components of the velocity vector-field $u^\mu$.

We frequently, and without comment, use the following product estimate:

#### Lemma 3.6

(Sobolev product estimates) If $s>n/p = 3/2$ then

$$\begin{aligned} \Vert uv \Vert _{H^s} \lesssim \Vert u \Vert _{H^s}\Vert v \Vert _{H^s}. \end{aligned}$$

We conclude this subsection with a result concerning elliptic regularity, see e.g. [4, App. H].

#### Lemma 3.7

(Elliptic regularity using $\mathcal {L}_{g, \gamma }$) Let *V* be a symmetric (0, 2)-tensor on *M*. There exist constants $C_1, C_2>0$ such, that for all $s \in \mathbb {Z}_{\ge 0}$,

$$\begin{aligned} C_1\Vert V \Vert _{H^{k+2s}} \le \Vert \mathcal {L}_{g, \gamma }^s V\Vert _{H^k} \le C_2\Vert V\Vert _{H^{k+2s}}\,, \end{aligned}$$

where $\mathcal {L}_{g, \gamma }^s$ denotes *s*-copies of $\mathcal {L}_{g, \gamma }$.

### Energy for the Perturbation of the Geometry

As noted in [2], in the spatially harmonic gauge (2.1) we have

$$\begin{aligned} \text {Ric}[g]_{ab}+\frac{2}{9} g_{ab} = \frac{1}{2} \mathcal {L}_{g, \gamma }(g-\gamma )_{ab} + J_{ab}, \end{aligned}$$

where $J_{ab}$ are higher-order terms (writen as $S_{ab}$ in [2, pg. 22]) satisfying, for $k\ge 1$,

$$\begin{aligned} \Vert J\Vert _{H^{k-1}}\le C \Vert g-\gamma \Vert _{H^k}. \end{aligned}$$

Following [1, 3], we define an energy for the geometric perturbation of the first and second fundamental forms by using $\mathcal {L}_{g, \gamma }$. This energy will fulfill a strong decay estimate enabled by the inclusion of certain correction terms $\Gamma _{(m)}$.

#### Definition 3.8

(Geometric energy ${E^g}_k$) Let $\lambda _0$ be the lowest eigenvalue of the operator $\mathcal {L}_{g, \gamma }$, with lower bounds given in Proposition 3.3. We define the correction parameter $\alpha =\alpha (\lambda _0,\delta _\alpha )$ by

$$\begin{aligned} \alpha :={\left\{ \begin{array}{ll} 1&  \lambda _0>1/9\\ 1-\delta _\alpha &  \lambda _0=1/9, \end{array}\right. } \end{aligned}$$

where $\delta _\alpha =\sqrt{1-9(\lambda _0-\varepsilon ')}$ with $1\gg \varepsilon '>0$ remains a variable to be determined in the course of the argument to follow. By fixing $\varepsilon '$ once and for all, $\delta _\alpha$ can be made suitably small when necessary. The corresponding correction constant, relevant for defining the corrected energies, is defined by

$$\begin{aligned} c_E:={\left\{ \begin{array}{ll} 1&  \lambda _0>1/9\\ 9(\lambda _0-\varepsilon ')&  \lambda _0=1/9. \end{array}\right. } \end{aligned}$$

We are now ready to define the energy for the geometric perturbation. For $m,k \in \mathbb {Z}_{\ge 1}$ let

$$\begin{aligned} \mathcal {E}_{(m)}&:=\frac{1}{2}\left( v,\mathcal {L}_{g,\gamma }^{m-1}( v) \right) _{L^2_{g,\gamma }}+\frac{9}{2}\left( h,\mathcal {L}_{g,\gamma }^{m}(h)\right) _{L^2_{g,\gamma }},\qquad \Gamma _{(m)}:=\left( v,\mathcal {L}_{g,\gamma }^{m-1}(h)\right) _{L^2_{g,\gamma }}. \end{aligned}$$

The energy measuring the geometric perturbation is then defined by

$$\begin{aligned} {E^g}_k:=\sum _{1\le m\le k} \big (\mathcal {E}_{(m)}+c_E\Gamma _{(m)}\big ). \end{aligned}$$

We will see later that the corrected geometric energy ${E^g}_k$ is in fact coercive over the standard Sobolev norms of the geometric variables $g, \Sigma$.

#### Definition 3.9

(Operators $\hat{\partial }_0, \partial _{\textbf{u}}$) We define the operators on *M*

$$\begin{aligned} \hat{\partial }_0:=\partial _T + \mathcal {L}_X, \qquad \partial _{\textbf{u}}:=u^0 \partial _T - \tau u^a \hat{\nabla }_a . \end{aligned}$$

The following identity, taken from [8], holds for some function *f* on *M*:

3.2 $$\begin{aligned} \partial _T \int _M f \mu _g = 3 \int _M \widehat{N}f \mu _g + \int _M \hat{\partial }_0(f) \mu _g \,. \end{aligned}$$

#### Remark 3.10

(Regularity parameters $\ell , N$) At top-order our bootstrap assumptions will involve Sobolev norms $H^N$ where *N* is a large integer. It is convenient to require *N* to be odd so that we can introduce $\ell \in \mathbb {Z}$ satisfying $\ell =\frac{N-1}{2}$.

We end this section with the top-order geometric energy for the geometric variables $g, \Sigma$, which crucially involves the fluid operator $\partial _{\textbf{u}}$. This energy will also fulfill a strong decay estimate enabled by the inclusion of the correction terms.

#### Definition 3.11

($E^g_{\partial _{\textbf{u}}, 2\ell }$ and $E_{tot}$) For $s \in \mathbb {Z}_{\ge 1}$, define

$$\begin{aligned}\begin{aligned} \mathcal {E}^g_{\partial _{\textbf{u}},2s}&:=\frac{9}{2} \left( \partial _{\textbf{u}}\mathcal {L}_{g, \gamma }^{s}(h),\partial _{\textbf{u}}\mathcal {L}_{g, \gamma }^{s}(h)\right) _{L^2_{g,\gamma }}+ \frac{1}{2}\left( \partial _{\textbf{u}}\mathcal {L}_{g, \gamma }^{s} v ,\partial _{\textbf{u}}\mathcal {L}_{g, \gamma }^{s-1} v \right) _{L^2_{g,\gamma }} , \\ \Gamma ^g_{\partial _{\textbf{u}},2s}&:=\left( \partial _{\textbf{u}}\mathcal {L}_{g, \gamma }^{s-1}v,\partial _{\textbf{u}}\mathcal {L}_{g, \gamma }^{s}(h)\right) _{L^2_{g,\gamma }}.\end{aligned} \end{aligned}$$

The corrected $\partial _{\textbf{u}}-$*boosted* geometric energy is then given by

$$\begin{aligned} {E^g_{\partial _{\textbf{u}},N-1}}:=\sum _{s=1}^\ell \Big (\mathcal {E}^g_{\partial _{\textbf{u}},2s}+ c_E \Gamma ^g_{\partial _{\textbf{u}},2s}\Big ). \end{aligned}$$

Finally we define

$$\begin{aligned} E^g_{tot}:={E^g_{\partial _{\textbf{u}},N-1}}+ E^g_{N-1}. \end{aligned}$$

## The Bootstrap Argument

In this section we first state the local-existence theory for the Einstein-Dust system in CMCSH gauge. Then we introduce the bootstrap assumptions on our solution and give some immediate consequences of these estimates.

### Local Existence

#### Theorem 4.1

Let $$N\ge 6$$. Consider CMC initial data $$(g_0,k_0,N_0,X_0,\rho _0,u_0)\in H^{N}\times H^{N-1}\times H^N\times H^N\times H^{N-2}\times H^{N-1}$$ at $$T=T_0$$ such that the constraints (2.7a) hold. Then there exists a unique classical solution $$(g,k,N,X,\rho ,u)$$ on $$[T_0,T_+)$$ for $$T_+>T_0$$ to the system (2.7), which is consequently also a solution to the Einstein-Dust equations. The components have the following regularity features

$$\begin{aligned}\begin{aligned} g,N,X \in C^0([T_0,T_+),H^N)\cap C^1([T_0,T_+),H^{N-1}),\\ k\in C^0([T_0,T_+),H^{N-1})\cap C^1([T_0,T_+),H^{N-2}),\\ u\in C^{0}([T_0,T_+],H^{N-1}),\\ \rho ,\partial _ {\textbf{u}}\rho ,\partial _{\textbf{u}}u \in C^{0}([T_0,T_+],H^{N-2}).\end{aligned} \end{aligned}$$

Furthermore, the time of existence and the norms of the solution depend continuously on the initial data. For the maximal time of existence $T_\infty$ we have either $T_\infty =+\infty$ or

$$\begin{aligned}\begin{aligned} \lim _{T\nearrow T_\infty } \sup _{[T_0,T_\infty ]}&\Vert g-\gamma \Vert _{H^N}+ \Vert \Sigma \Vert _{H^{N-1}}+\Vert N-3\Vert _{H^N}+\Vert X\Vert _{H^N}\\&+\Vert \partial _T N\Vert _{H^{N-1}}+\Vert \partial _T X\Vert _{H^{N-1}}+\Vert \rho \Vert _{H^{N-2}}+|\tau |\Vert u\Vert _{H^{N-1}}> \delta (\gamma ),\end{aligned}\end{aligned}$$

where $\delta (\gamma )$ is a positive fixed constant depending only on the background metric.

#### Proof

The proof follows analogous to [16, Theorem 3.5] where the control on the lapse and shift are replaced by the elliptic techniques applied in [11]. The adaption of the regularity scheme to avoid the loss of derivatives from [16] to the present case is executed in detail in the global analysis discussed in the remainder of the paper. The smallness condition in the continuation criterion stems from a smallness requirement in applying the corresponding elliptic equation for the lapse. $\square$

#### Remark 4.2

Since we apply the local-existence theorem only for data close to the background solution the smallness condition required to extend the solution for arbitrarily large times is automatically fulfilled when our smallness conditions hold, which we prove by a bootstrap argument. Since the smallness parameter of the continuation criterion depends only on the background geometry we can choose the smallness of the initial data, which we do accordingly without mentioning it explicitly again.

### Bootstrap Assumptions

Let $\mu , \lambda$ be fixed positive constants with $\mu \ll 1$ and $\lambda <1$. We assume that, for all $T_0 \le T\le T'$, the following bootstrap assumptions hold (2.7):

4.1 $$\begin{aligned} \begin{aligned} \Vert {g-\gamma }\Vert _{H^N} +\Vert \Sigma \Vert _{H^{N-1}}&\le C \varepsilon e^{-\lambda T},\\ \Vert N-3\Vert _{H^{N}}+\Vert X\Vert _{H^{N}}&\le C\varepsilon e^{-T},\\ \Vert \partial _T N\Vert _{H^{N-1}}+\Vert \partial _T X\Vert _{H^{N-1}}&\le C\varepsilon e^{-T},\\ \Vert \rho \Vert _{H^{N-2}}&\le C \varepsilon , \\ \Vert u \Vert _{H^{N-1}}&\le C \varepsilon e^{\mu T}.\end{aligned}\end{aligned}$$

there $T'<T_\infty$ is fixed. We hereon assume that (4.1) hold and *do not repeat this fact*. Recall also Remark 3.5 regarding the norm on *u*.

#### Definition 4.3

($\Lambda (T)$) It is convenient to introduce the notation

$$\begin{aligned} \Lambda (T) :=\Vert \widehat{N}\Vert _{H^N} + \Vert X\Vert _{H^N} + \Vert \partial _T N \Vert _{H^{N-1}} + \Vert \partial _T X \Vert _{H^{N-1}} + |\tau | \Vert \rho \Vert _{H^{N-2}} + \tau ^2 \Vert u \Vert _{H^{N-1}}^2. \end{aligned}$$

Note that, under the bootstrap assumptions (4.1), $\Lambda (T) \lesssim \varepsilon e^{-T}$.

We state some immediate consequences of the bootstrap assumptions regarding $u^0$ which we use without further comment. Using Lemma 2.3, we have

4.2 $$\begin{aligned} \begin{aligned} \Vert \hat{u}^0\Vert _{H^{N-1}}&\lesssim \varepsilon , \\ \Vert \nabla \hat{u}^0\Vert _{H^{N-2}}&\lesssim \Vert \nabla N \Vert _{H^{N-2}} + \tau ^2 \Vert u^a \nabla u^a \Vert _{H^{N-2}} + \text {h.o.t} \lesssim \Lambda (T).\end{aligned}\end{aligned}$$

Note the first estimate does not pick up any $\mu -$loss. By the Sobolev embedding $H^2 \hookrightarrow L^\infty$, $\Vert \hat{u}^0\Vert _{L^\infty }= \Vert u^0-1/3\Vert _{L^\infty }\le 1/10$ and thus

$$\begin{aligned} \Vert u^0\Vert _{L^\infty }\lesssim 1, \quad \Vert u^0\Vert _{L^\infty }^{-1} \lesssim 1. \end{aligned}$$

The next Lemma concerning the dust matter components is indicative of the good behaviour that, as we discussed in Sect. 1.2.4, we roughly expect as the speed of sound is reduced.

#### Lemma 4.4

(Estimates on matter components) We have

$$\begin{aligned}\begin{aligned} |\tau |\big (\Vert E\Vert _{H^{N-2}} + \Vert \eta \Vert _{H^{N-2}}+ \Vert S\Vert _{H^{N-2}} \big )&\lesssim \Lambda (T),\\ |\tau |^2 \Vert \jmath \Vert _{H^{N-2}}\lesssim \varepsilon e^{(-1+\mu )T} \Lambda (T), \quad |\tau |^3 \Vert \underline{T}\Vert _{H^{N-2}}&\lesssim \Lambda (T)^2.\end{aligned}\end{aligned}$$

#### Proof

The estimates are immediate by distributing derivatives across the terms written in Definition 2.6 and using Lemma 3.6. $\square$

#### Lemma 4.5

(Geometric coercivity estimate) Let $s\in \mathbb {Z}$ such that $1\le s \le N-1$. There is a $\delta >0$ and a constant $C>0$ such that for $(g,\Sigma )\in B_{\delta }((\gamma ,0))$ the following inequality holds

$$\begin{aligned} \Vert g-\gamma \Vert _{H^{s}}^2 + \Vert \Sigma \Vert _{H^{s-1}}^2 \le C E^g_{s}. \end{aligned}$$

#### Proof

The proof of this lemma follows verbatim from [1, Lemma 19], which itself follows from [3, Lemma 7.2], and the eigenvalue estimates referred to in Proposition 3.3. $\square$

The next Lemma is actually only used once in our entire argument. Its significance lies in the fact that it allows us to convert the quadratic derivative term $(\nabla V)^2$ appearing in the $H^1$ Sobolev norm into just one second-order derivative as $V \cdot \mathcal {L}_{g, \gamma }V$, which will more naturally be controlled by $E^g_{\partial _{\textbf{u}}, N-1}$.

#### Lemma 4.6

Let *V* be a symmetric (0, 2)-tensor on *M*. Then,

$$\begin{aligned} \Vert V\Vert _{H^1} \lesssim \Vert V\Vert _{L^2} + (V, \mathcal {L}_{g, \gamma }V)_{L^2_{g,\gamma }}^{1/2}. \end{aligned}$$

#### Proof

We integrate by parts, use the closeness between the *g* and $\gamma$ metrics and the boundedness of the $\text {Riem}[\gamma ]$ components:

$$\begin{aligned}\begin{aligned} \Vert V\Vert _{H^1}^2&= \Vert V\Vert _{L^2}^2 + \int _M g^{ab} g^{ij} g^{kl} \nabla _a V_{ik} \nabla _b V_{jl} \, \mu _g \\  &\le \Vert V\Vert _{L^2}^2 +\Big | \int _M g^{ab} g^{ij} g^{kl} V_{ik} \nabla _a \nabla _b V_{jl} \, \mu _g \Big | \\  &\le \Vert V\Vert _{L^2}^2 + \Big | \int _M \langle V,\mathcal {L}_{g, \gamma }V \rangle _\gamma \, \mu _g + 2 \int _M \langle V, \text{ Riem }[\gamma ]\circ V \rangle _\gamma \, \mu _g \Big | \\  &\lesssim \Vert V\Vert _{L^2}^2 + (V, \mathcal {L}_{g, \gamma }V)_{L^2_{g,\gamma }}.\end{aligned}\end{aligned}$$

$$\square $$

## Preliminary Estimates

This section is concerned with deriving several preliminary estimates that are required for our later energy inequalities. There are estimates on commutator terms (Sects. 5.1, 5.4), estimates on matter terms (Sect. 5.3) and also estimates on geometric variables (Sects. 5.2, 5.5). We also present an integration by parts Lemma 5.3, in particular (5.6c), which later plays an important role in removing various critical terms that arise during the energy estimates.

### First Commutator Estimates

In this subsection we let *V* be an arbitrary symmetric (0, 2)-tensor and $\phi$ a scalar, unless otherwise specified. We begin with the identity

5.1 $$\begin{aligned} [\partial _T, \nabla _a]V_{i j} = (-\partial _T \Gamma ^c_{ai})V_{c j} + (-\partial _T \Gamma ^c_{aj})V_{i c}. \end{aligned}$$

The terms $\partial _T \Gamma [g]$ can be estimated (see [1, (10.12)]), for $0 \le k \le N-2$, by:

5.2 $$\begin{aligned} \Vert \partial _T \Gamma (g)\Vert _{H^k} \lesssim \Vert \Sigma \Vert _{H^{k+1}} + \Vert X\Vert _{H^{k+2}} + \Vert \widehat{N}\Vert _{H^{k+1}}. \end{aligned}$$

We also have the following commutator identities:

5.3 $$\begin{aligned} [\partial _T,\nabla _{a_1}\cdots \nabla _{a_k}]\phi&= [\partial _T,\nabla _{a_1}]\nabla _{a_2}\cdots \nabla _{a_k} \phi + \nabla _{a_1} [\partial _T,\nabla _{a_2}]\nabla _{a_3}\cdots \nabla _{a_k} \phi \nonumber \\&\quad +\ldots + \nabla _{a_1}\cdots \nabla _{a_{k-2}} [\partial _T,\nabla _{a_{k-1}}] \nabla _{a_k} \phi + \nabla _{a_1}\cdots \nabla _{a_{k-1}} [\partial _T,\nabla _{a_{k}}] \phi , \end{aligned}$$

5.4 $$\begin{aligned} [\nabla _i,\nabla _{a_1}\cdots \nabla _{a_k}]\phi&= [\nabla _i,\nabla _{a_1}]\nabla _{a_2}\cdots \nabla _{a_k} \phi + \nabla _{a_1} [\nabla _i,\nabla _{a_2}]\nabla _{a_3}\cdots \nabla _{a_k} \phi \nonumber \\&\quad +\ldots + \nabla _{a_1}\cdots \nabla _{a_{k-2}} [\nabla _i,\nabla _{a_{k-1}}] \nabla _{a_k} \phi + \nabla _{a_1}\cdots \nabla _{a_{k-1}} [\nabla _i,\nabla _{a_{k}}] \phi . \end{aligned}$$

Note the last terms in each of (5.3) and (5.4) will in fact vanish since the metric *g* is torsion free.

In the next part of this subsection we state an important estimate, given in (5.5), that allows us to turn background $\hat{\nabla }$ derivatives into dynamical $\nabla$ ones.

#### Definition 5.1

(Difference tensor $\Upsilon$) Recalling that $\nabla$ and $\hat{\nabla }$ are the Levi-Civita symbols of *g* and $\gamma$ respectively, we define $\Upsilon$ a (1,2)-tensor by

$$\begin{aligned} \Upsilon ^a_{bc} :=\Gamma ^a_{bc}[g]-\Gamma ^a_{bc}[\gamma ]. \end{aligned}$$

Let *V* be a vector and *P* a one-form. Then we have

$$\begin{aligned} \nabla _a V^{i}= \hat{\nabla }_a V^i + \Upsilon ^i_{ja}V^{j}, \qquad \nabla _a P_i= \hat{\nabla }_a P_i - \Upsilon ^j_{ia}P_j. \end{aligned}$$

We will often schematically write tensorial contractions using $*$. For example,

$$\begin{aligned} \nabla _a V^{i}= \hat{\nabla }_a V^i + \Upsilon ^i_{ja}V^{j}, \quad \text { becomes } \quad \nabla V = \hat{\nabla }V + \Upsilon *V. \end{aligned}$$

In local coordinates the components of the $\Upsilon$ tensor are given by

$$\begin{aligned}\begin{aligned} \Upsilon ^a_{bc}&=-\frac{1}{2} \gamma ^{ai}\left( \nabla _b \gamma _{ci} + \nabla _c \gamma _{ai} - \nabla _i \gamma _{bc} \right) =\frac{1}{2} \gamma ^{ai}\left( \nabla _b h_{ci} + \nabla _c h_{ai} - \nabla _i h_{bc} \right) .\end{aligned}\end{aligned}$$

#### Lemma 5.2

For $0\le k \le N-2$, we have

$$\begin{aligned}\begin{aligned} \Vert [\hat{\nabla }, \nabla ] V\Vert _{H^k}&\lesssim \Vert V\Vert _{H^k} + \varepsilon e^{-\lambda T}\Vert V\Vert _{H^{k+1}}.\end{aligned} \end{aligned}$$

#### Proof

First we see that from Definition 5.1, for all $0 \le k \le N-1$,

5.5 $$\begin{aligned} \Vert \Upsilon \Vert _{H^k} \lesssim \Vert g-\gamma \Vert _{H^{k+1}}. \end{aligned}$$

Thus we compute,

$$\begin{aligned}\begin{aligned} [\hat{\nabla }_c, \nabla _a] V_{i j}&= [\hat{\nabla }_c, \hat{\nabla }_a] V_{i j}+ \Upsilon ^b_{ca}\hat{\nabla }_b V_{i j} - \big ((\hat{\nabla }_c \Upsilon ^b_{ai})V_{b j} + (\hat{\nabla }_c \Upsilon ^b_{aj}) V_{i b}\big ).\end{aligned} \end{aligned}$$

Using the boundedness of the $\text {Riem}[\gamma ]$ components, the required estimate then follows. $\square$

We end this subsection with some very useful estimates that come from integration by parts.

#### Lemma 5.3

(Integration by parts) Let *T* be an arbitrary vectorfield and *V*, *P* arbitrary symmetric (0, 2)-tensors, on *M*. Then

5.6a $$\begin{aligned} (\mathcal {L}_{g, \gamma }V, P)_{L^2(g, \gamma )} = \int _M \langle g^{ab} \hat{\nabla }_a V, \hat{\nabla }_b P \rangle _\gamma \mu _g -2 \int _M \langle \text {Riem}[\gamma ]_{\bullet a\bullet b} V^{ab}, P\rangle _\gamma \mu _g . \nonumber \\ \end{aligned}$$

and

5.6b $$\begin{aligned} \big | (T^a \hat{\nabla }_a V, P)_{L^2_{g,\gamma }} \big |&\lesssim \Vert T \Vert _{H^3} \Vert V \Vert _{L^2} \Vert P\Vert _{H^1}, \end{aligned}$$

5.6c $$\begin{aligned} \big | (T^a \hat{\nabla }_a V, V)_{L^2_{g,\gamma }} \big |&\lesssim \Vert T \Vert _{H^3} \Vert V \Vert _{L^2}^2. \end{aligned}$$

#### Proof

The proof of (5.6a) follows by using the gauge condition $H^a = 0$. To show (5.6b), recall that the Jacobi identity implies that

$$\begin{aligned} \hat{\nabla }_a \sqrt{\det g} = \frac{1}{2} \sqrt{\det g} \cdot g^{ij}(\hat{\nabla }_a g_{ij}). \end{aligned}$$

Using this we find that

$$\begin{aligned} (T^a \hat{\nabla }_a V, P)_{L^2_{g,\gamma }}&= \int _M \gamma ^{ij}\gamma ^{kl} T^a (\hat{\nabla }_a V_{ik}) P_{jl} \, \sqrt{\det g} \\&= -\int _M \gamma ^{ij}\gamma ^{kl} \hat{\nabla }_a \left( T^a P_{jl} \frac{\sqrt{\det g}}{\sqrt{\det \gamma }}\right) V_{ik} \sqrt{\det \gamma } \\  &= - (V, T^a \hat{\nabla }_a P)_{L^2_{g,\gamma }} - ((\hat{\nabla }_a T^a) V, P)_{L^2_{g,\gamma }}\\&\quad - \tfrac{1}{2} (V, (T^a g^{bc}\hat{\nabla }_a g_{bc}) P)_{L^2_{g,\gamma }}. \end{aligned}$$

Thus,

$$\begin{aligned} \big | (T^a \hat{\nabla }_a V, P)_{L^2_{g,\gamma }} \big |&= \big | - (V, T^a \hat{\nabla }_a P)_{L^2_{g,\gamma }} - ((\hat{\nabla }_a T^a) V, P)_{L^2_{g,\gamma }} \\&\quad - \tfrac{1}{2} (V, (T^a g^{bc}\hat{\nabla }_a g_{bc}) P)_{L^2_{g,\gamma }} \big | \\  &\lesssim \big ( \Vert T^a \Vert _{H^3} + \Vert T^a \Vert _{H^2} \Vert g-\gamma \Vert _{H^3} \big ) \Vert V \Vert _{L^2} \Vert P\Vert _{H^1}. \end{aligned}$$

Crucially, in the symmetric case, we can bring one term over to the left hand side to show that

$$\begin{aligned} \begin{aligned} \big | (T^a \hat{\nabla }_a V, V)_{L^2_{g,\gamma }} \big |&= \big | - \tfrac{1}{2} ((\hat{\nabla }_a T^a) V, V)_{L^2_{g,\gamma }} - \tfrac{1}{4} (V, (T^a g^{bc}\hat{\nabla }_a g_{bc}) V)_{L^2_{g,\gamma }} \big | \\  &\lesssim \big ( \Vert T^a \Vert _{H^3} + \Vert T^a \Vert _{H^2} \Vert g-\gamma \Vert _{H^3} \big ) \Vert V \Vert _{L^2}^2.\end{aligned} \end{aligned}$$

$$\square $$

### First Estimates on Geometric Components

We now establish some of our first estimates on the ADM variables *N*, *X* and the geometric variables $g, \Sigma$.

#### Lemma 5.4

(Dust derivatives of lapse and shift) For $2\le k \le N-1$,

$$\begin{aligned} \begin{aligned} \Vert \partial _{\textbf{u}}N \Vert _{H^k} + \Vert \partial _{\textbf{u}}X \Vert _{H^k} \lesssim \Lambda (T).\end{aligned} \end{aligned}$$

#### Proof

Since the lapse is a scalar, $\partial _{\textbf{u}}N = u^0 \partial _T N - \tau u^c \nabla _c N$. For $\partial _{\textbf{u}}X^a$ we use (5.5). We find that

$$\begin{aligned}\begin{aligned} \Vert \partial _{\textbf{u}}N \Vert _{H^k} + \Vert \partial _{\textbf{u}}X \Vert _{H^k} \lesssim \Vert \partial _T N \Vert _{H^k} + \Vert \partial _T X \Vert _{H^k} + \varepsilon e^{(-1+\mu ) T} \Lambda (T).\end{aligned} \end{aligned}$$

$$\square $$

#### Lemma 5.5

(Estimates on geometric source terms) We have

$$\begin{aligned}\begin{aligned} \Vert F_h \Vert _{H^k}&\lesssim \Vert \widehat{N}\Vert _{H^k} + \Vert X\Vert _{H^{k+1}}^2 + \Vert g-\gamma \Vert _{H^{k+1}}^2,\\&\quad 2 \le k \le N-1, \\ \Vert F_v\Vert _{H^k}&\lesssim \Vert \widehat{N}\Vert _{H^{k+2}} + |\tau | \Vert \rho \Vert _{H^k} + \Vert X\Vert _{H^{k+1}}^2 \\  &\quad + \Vert g -\gamma \Vert _{H^{k+1}}^2 + \Vert \Sigma \Vert _{H^{k}}^2,\\&\quad 2 \le k \le N-2,\end{aligned} \end{aligned}$$

and thus

$$\begin{aligned} \Vert F_h \Vert _{H^{N-1}} + \Vert F_v \Vert _{H^{N-2}} \lesssim \Lambda (T) + \Vert g-\gamma \Vert _{H^N}^2 + \Vert \Sigma \Vert _{H^{N-1}}^2. \end{aligned}$$

#### Proof

Using the product estimate of Lemma 3.6 and (5.5) we obtain, for $2 \le k \le N-1$,

$$\begin{aligned}\begin{aligned} \Vert F_h\Vert _{H^k}&\lesssim \Vert \widehat{N}\Vert _{H^k} + \Vert h \Vert _{H^k} \Vert \nabla X + \Upsilon *X\Vert _{H^{k}} \\&\lesssim \Vert \widehat{N}\Vert _{H^k} + \Vert g-\gamma \Vert _{H^k} \Vert X\Vert _{H^{k+1}} + \Vert g-\gamma \Vert _{H^{k+1}}^2 \Vert X\Vert _{H^{k}}.\end{aligned} \end{aligned}$$

Using, in addition, Lemma 4.4, we see, for $2 \le k \le N-2$,

$$\begin{aligned} \begin{aligned} \Vert F_v \Vert _{H^k}&\lesssim \Vert \widehat{N}\Vert _{H^{k+2}} + \Vert \Sigma \Vert _{H^k}^2 + |\tau | \Vert S_{ij} \Vert _{H^k} + \Vert \Sigma \Vert _{H^k} \Vert \hat{\nabla }X\Vert _{H^{k}} .\end{aligned} \end{aligned}$$

$$\square $$

We can now combine the geometric source term estimates from the previous lemma with the equations of motion given in (2.8).

#### Lemma 5.6

(Time derivatives of *g* and $\Sigma$) The following estimates hold:

$$\begin{aligned}\begin{aligned} \Vert \partial _T g\Vert _{H^k}&\lesssim \Vert \Sigma \Vert _{H^{k}} + \Vert g-\gamma \Vert _{H^{k+1}} +\Vert \widehat{N}\Vert _{H^k} + \Vert X\Vert _{H^{k+1}} ,&2\le k \le N-1, \\ \Vert \partial _T \Sigma \Vert _{H^k}&\lesssim \Vert \Sigma \Vert _{H^{k+1}} + \Vert g-\gamma \Vert _{H^{k+2}} +\Vert \widehat{N}\Vert _{H^{k+2}}\\&\quad + \Vert X\Vert _{H^{k+1}} + |\tau | \Vert \rho \Vert _{H^k},&2\le k \le N-2.\end{aligned} \end{aligned}$$

#### Proof

Using Lemma 5.5, (2.8) and (5.5) we find

$$\begin{aligned}\begin{aligned} \Vert \partial _T h \Vert _{H^k}&\lesssim \Vert \Sigma \Vert _{H^{k}} + \Vert X(\nabla h + \Upsilon *h)\Vert _{H^{k}} + \Vert F_h \Vert _{H^k} \\&\lesssim \Vert \Sigma \Vert _{H^{k}} +\Vert \widehat{N}\Vert _{H^k} + \Vert X\Vert _{H^{k+1}}^2 + \Vert g-\gamma \Vert _{H^{k+1}}^2\,,\end{aligned} \end{aligned}$$

and, using additionally Lemma 3.7,

$$\begin{aligned} \begin{aligned} \Vert \partial _T v\Vert _{H^k}&\lesssim \Vert \Sigma \Vert _{H^{k}} +\Vert \mathcal {L}_{g, \gamma }h\Vert _{H^{k}}+ \Vert X(\nabla v + \Upsilon *v)\Vert _{H^{k}} + \Vert F_v \Vert _{H^k} \\  &\lesssim \Vert \Sigma \Vert _{H^{k}} + \Vert g-\gamma \Vert _{H^{k+2}} +\Vert \widehat{N}\Vert _{H^{k+2}}\\&\quad + \Vert X\Vert _{H^{k+1}}^2 + \Vert \Sigma \Vert _{H^{k+1}}^2 + |\tau | \Vert \rho \Vert _{H^k}.\end{aligned} \end{aligned}$$

$$\square $$

#### Corollary 5.7

(Dust derivatives of *g* and $\Sigma$) The following estimates hold:

$$\begin{aligned} \begin{aligned} \Vert \partial _{\textbf{u}}g\Vert _{H^k}&\lesssim \Vert \Sigma \Vert _{H^{k}} + \Vert g-\gamma \Vert _{H^{k+1}} +\Lambda (T) ,&2\le k \le N-1, \\ \Vert \partial _{\textbf{u}}\Sigma \Vert _{H^k}&\lesssim \Vert \Sigma \Vert _{H^{k+1}} + \Vert g-\gamma \Vert _{H^{k+2}} +\Lambda (T),&2\le k \le N-2.\end{aligned} \end{aligned}$$

#### Proof

Writing

$$\begin{aligned} \partial _{\textbf{u}}V_{ab} = u^0 \partial _T V_{ab}- \tau u^c \nabla _c V_{ab}- \tau u^c\Upsilon ^d_{ca} V_{db} - \tau u^c\Upsilon ^d_{cb} V_{ad} \end{aligned}$$

and using Lemma 5.6 gives the estimates. $\square$

#### Remark 5.8

It is unsurprising that for the geometric variables $g, \Sigma$, the fluid derivative estimates in Corollary 5.7 do not gain us any improved information compared to the time derivative estimates in Lemma 5.6. This will of course change when we consider instead estimates on certain fluid matter variables which are naturally more compatible with the fluid derivative operator $\partial _{\textbf{u}}$.

### First Estimates on Fluid Components

In this subsection we establish further estimates on the various matter variables. Note that we need to estimate both matter components coming from contractions with the stress energy tensor (see Definition 2.6) and the fluid source terms terms appearing in the equations of motion (see Definition 2.7).

#### Lemma 5.9

(Fluid source term estimates) We have, for some $\nu >0$,

$$\begin{aligned}\begin{aligned} \Vert F_\rho \Vert _{H^{N-2}}&\lesssim |\tau | \Vert u\Vert _{H^{N-1}} + \Vert \Sigma \Vert _{H^{N-2}}+ \Lambda (T), \\ \Vert F_{u^0} \Vert _{H^{N-1}}&\lesssim \Vert \Sigma \Vert _{H^{N-1}}^2 + \Lambda (T), \\ \Vert F_{u^j} \Vert _{H^{N-1}}&\lesssim |\tau |^{-1} \Lambda (T) + \varepsilon ^2 e^{-\nu T}.\end{aligned} \end{aligned}$$

#### Proof

For $2\le k \le N-2$ we distribute derivatives across the terms given in Definitions 2.4 and 2.7. This yields

$$\begin{aligned}\begin{aligned} \Vert F_\rho \Vert _{H^k}&\lesssim |\tau | \Vert \nabla u^j \Vert _{H^{k}} + \Vert u^0\Vert _{L^\infty } \Vert \Gamma ^i_i\Vert _{H^k} + |\tau | \Vert \Gamma _j\Vert _{H^k}\Vert u \Vert _{H^k}\\&\quad + |\tau |\Vert \Gamma _{ik} \Vert _{H^k}\Vert X\Vert _{H^k}\Vert u \Vert _{H^k} + \tau ^2 \Vert u\Vert _{H^k}^2 \Vert \Gamma _{kj}\Vert _{H^{k}} \\&\lesssim |\tau | \Vert u\Vert _{H^{k+1}} + \Vert \Sigma \Vert _{H^k}+ \Vert X\Vert _{H^{k+1}}.\end{aligned} \end{aligned}$$

Similarly, for $$2\le k \le N-1$$,

$$\begin{aligned} \begin{aligned} \Vert F_{u^0} \Vert _{H^k}&\lesssim \Vert \Gamma _R\Vert _{H^k}+ |\tau |\Vert u\Vert _{H^k} \Vert \Gamma _j \Vert _{H^k} + \tau ^2 \Vert u\Vert _{H^k}^2 \Vert \Gamma _{jk} \Vert _{H^k} \\&\lesssim \Vert \partial _T N \Vert _{H^k} + \Vert \Sigma \Vert _{H^k}^2+ \Vert \widehat{N}\Vert _{H^{k+1}}^2+ \Vert X\Vert _{H^{k}}^2 + \tau ^2 \Vert u\Vert _{H^k}^2.\end{aligned} \end{aligned}$$

Finally, for $$2\le k \le N-1$$,

$$\begin{aligned} \begin{aligned} \Vert F_{u^j} \Vert _{H^k}&\lesssim |\tau |^{-1} \Vert \overset{\circ }{\Gamma } ^j\Vert _{H^k}+ \Vert u \Vert _{H^k}^2 \big ( \Vert \Gamma [g]-\Gamma [\gamma ]\Vert _{H^k}\\&\quad + |\tau |\Vert X\Vert _{H^k} \Vert \Gamma _{ki}\Vert _{H^k}\big )\\&\quad + \Vert u\Vert _{H^k} \Vert \Gamma ^i_j \Vert _{H^k} \\&\lesssim |\tau |^{-1} \big (\Vert \partial _T X \Vert _{H^k} + \Vert X\Vert _{H^{k+1}} + \Vert \widehat{N}\Vert _{H^{k+1}}^2 \\&\quad + \Vert \Sigma \Vert _{H^k}^2 + \Vert X\Vert _{H^k} \Vert \partial _T N \Vert _{H^k} \big ) \\&\quad + \Vert u \Vert _{H^k}^2 \big ( \Vert g-\gamma \Vert _{H^{k+1}} + |\tau | \Vert X\Vert _{H^k}\big ) \\&\quad + \Vert u\Vert _{H^k} \big ( \Vert \Sigma \Vert _{H^k} + \Vert \widehat{N}\Vert _{H^{k}} + \Vert X\Vert _{H^{k+1}} \big ) \\&\lesssim |\tau |^{-1} \Lambda (T) + \varepsilon ^2 e^{(1-2\lambda ) T} + \varepsilon ^2 e^{(-\lambda +2\mu )T}.\end{aligned} \end{aligned}$$

It is convenient also to note that at lower order we can apply Lemma 4.5 to find

5.7 $$\begin{aligned} \begin{aligned} |\tau | \Vert F_{u^j} \Vert _{H^{N-2}}&\lesssim E^g_{N-2} + \Lambda (T) + |\tau |\Vert u\Vert _{H^{N-2}}^2 (E^g_{N-1})^{1/2}.\end{aligned} \end{aligned}$$

$$\square $$

In the next lemma we provide estimates for fluid derivatives of certain matter components appearing in Definition 2.6. The weights in $\tau$ are included for convenience since these expressions appear later on in the energy estimates.

#### Lemma 5.10

(Dust derivatives of matter components) We have

$$\begin{aligned}\begin{aligned} |\tau | \Vert \partial _{\textbf{u}}\eta \Vert _{H^{N-2}} + |\tau |\Vert \partial _{\textbf{u}}S \Vert _{H^{N-2}}&\lesssim \varepsilon e^{\max \{-1+\mu ,-\lambda \} T}\Lambda (T) + \Lambda (T)^2,\\ |\tau |^2 \Vert \partial _{\textbf{u}}\jmath \Vert _{H^{N-2}}&\lesssim \Lambda (T)^2 + \varepsilon ^2 \Lambda (T) e^{(-\lambda +\mu )T}.\end{aligned} \end{aligned}$$

#### Proof

Note that (2.7d) can be rewritten as

$$\begin{aligned}\begin{aligned} \partial _{\textbf{u}}u^j&= \tau u^a u^c\Upsilon ^j_{ac} + \tau ^{-1}(u^0)^2 N\nabla ^j N+F_{u^j}, \qquad \partial _{\textbf{u}}u^0 =F_{u^0} , \qquad \partial _{\textbf{u}}\rho = \rho F_\rho .\end{aligned} \end{aligned}$$

Using Definition 2.6 we compute

$$\begin{aligned} \partial _{\textbf{u}}\eta&= \rho F_\rho \left( (u^0)^2 N^2 + g_{ab}(X^a u^0 + \tau u^a)(X^b u^0 + \tau u^b)\right) + 2 \rho (u^0)^2 N \partial _{\textbf{u}}N \\  &\quad + \rho (\partial _{\textbf{u}}g_{ab}) (X^a u^0 + \tau u^a)(X^b u^0 + \tau u^b) + 2 \rho g_{ab} u^0(X^b u^0 + \tau u^b) (\partial _{\textbf{u}}X^a) \\  &\quad +F_{u^0} \left( 2\rho u^0 N^2 + 2\rho g_{ab} X^a(X^b u^0 + \tau u^b) \right) + 2\rho g_{ab} u^0 u^a (X^b u^0 + \tau u^b) (\partial _T \tau ) \\  &\quad + 2\rho g_{ab} \tau (X^b u^0 + \tau u^b)\left( \tau u^a \Upsilon ^j_{ac} u^c + \tau ^{-1}(u^0)^2 N\nabla ^j N+F_{u^j}\right) . \end{aligned}$$

Thus, using Lemma 5.4, Corollary 5.7 and the matter estimates from Lemma 5.9, we obtain

$$\begin{aligned} |\tau |\Vert \partial _{\textbf{u}}\eta \Vert _{H^{N-2}}&\lesssim |\tau |\Vert \rho \Vert _{H^{N-2}}\Vert F_\rho \Vert _{H^{N-2}} + |\tau |\Vert \rho \Vert _{H^{N-2}}\Vert \partial _{\textbf{u}}N\Vert _{H^{N-2}}\\&\quad s +|\tau |\Vert \rho \Vert _{H^{N-2}} \Vert F_{u^0}\Vert _{H^{N-2}} + \text {h.o.t. } \\&\lesssim \varepsilon e^{\max \{-1+\mu ,-\lambda \}T}\Lambda (T) + \Lambda (T)^2. \end{aligned}$$

Again using (2.6) we compute

$$\begin{aligned} \partial _{\textbf{u}}\jmath ^a&= (\rho F_\rho ) N u^0 u^a + (\partial _{\textbf{u}}N) \rho u^0 u^a + (F_{u^0}) \rho N u^a + (\tau \Upsilon ^a_{bc}u^b u^c\\&\quad + \tau ^{-1}(u^0)^2 N\nabla ^a N+F_{u^a}) \rho N u^0. \end{aligned}$$

So, by the geometry estimates in Lemma 5.4 and 5.6, together with the fluid source term estimates in Lemma 5.9, we obtain

$$\begin{aligned} \begin{aligned} \tau ^2 \Vert \partial _{\textbf{u}}\jmath ^a\Vert _{H^{N-2}}&\lesssim |\tau | \Vert \rho \Vert _{H^{N-2}} \Vert \widehat{N}\Vert _{H^{N-1}} + |\tau |^2 \Vert \rho \Vert _{H^{N-2}}\Vert F_{u^a} \Vert _{H^{N-2}} + \text {h.o.t. } \\  &\lesssim \varepsilon ^2 \Lambda (T) e^{(-\lambda +\mu )T} + \Lambda (T)^2 .\end{aligned} \end{aligned}$$

Finally, from (2.6), we find that

$$\begin{aligned}\begin{aligned} \partial _{\textbf{u}}S_{ab}&= (\partial _{\textbf{u}}\rho )\big ( ( u^0 X_a+ \tau u_a )(u^0 X_b+ \tau u_b) +\tfrac{1}{2} g_{ab}\big ) + \tfrac{1}{2}\rho (\partial _{\textbf{u}}g_{ab}) +\text {h.o.t.}, \end{aligned} \end{aligned}$$

also

$$\begin{aligned}\begin{aligned} |\tau |\Vert \partial _{\textbf{u}}S\Vert _{H^{N-2}}&\lesssim |\tau | \Vert \rho \Vert _{H^{N-2}}\Vert F_\rho \Vert _{H^{N-2}} \lesssim \varepsilon e^{\max \{-1+\mu ,-\lambda \} T}\Lambda (T) + \Lambda (T)^2.\end{aligned} \end{aligned}$$

$$\square $$

#### Remark 5.11

The significance of using dust derivatives is made clear by look at the higher regularity appearing in Lemma 5.10 compared to the following Lemma 5.12 which only concerns time derivatives. In Lemma 5.10, we directly computed the $\partial _{\textbf{u}}$ derivatives using the equations of motion, instead of doing a rough estimate by expanding $\partial _{\textbf{u}}\sim \partial _T + \tau u^c *\nabla$.

#### Lemma 5.12

(Time derivatives of matter components) We have,

$$\begin{aligned}\begin{aligned} |\tau |\Vert \partial _T\eta \Vert _{H^{N-3}}&\lesssim \varepsilon e^{(-1+\mu )T} \Lambda (T) + \Lambda (T)^2, \\ \Lambda (T) \Vert \partial _T \jmath \Vert _{H^{N-3}}&\lesssim \varepsilon ^2 \Lambda (T) +\varepsilon ^4 e^{-(1+\nu )T}, \\ |\tau | \Vert \partial _TS \Vert _{H^{N-3}}&\lesssim \Lambda (T).\end{aligned} \end{aligned}$$

#### Proof

We calculate $\partial _T \eta$ explicitly from (2.6) as

$$\begin{aligned}\begin{aligned} \partial _T \eta&= \partial _T E+ \big (X_a X^a (u^0)^2 + 2\tau X_b u^b u^0 + \tau ^2 g_{ab} u^a u^b \big )\partial _T \rho \\&\quad + \big ( 2\rho X^a (u^0)^2 + 2\tau \rho u^a u^0\big )\partial _T X_a \\&\quad + \big ( 2u^0 \rho X_a X^a + 2 \tau \rho X_b u^b \big )\partial _T u^0 + \big ( 2\tau \rho X_b u^0 + 2 \tau ^2 \rho u_b\big )\partial _T u^b\\&\quad + \big ( 2\rho X_b u^b u^0+2 \tau \rho u_a u^a\big )\partial _T \tau .\end{aligned}\end{aligned}$$

Using $\partial _T\tau = -\tau$, we obtain

5.8 $$\begin{aligned} \begin{aligned} |\tau | \Vert \partial _T \eta \Vert _{H^{N-3}}&\lesssim |\tau | \Vert \partial _T E\Vert _{H^{N-3}} + \Lambda (T) |\tau | \Vert \partial _T\rho \Vert _{H^{N-3}}\\&\quad + \varepsilon e^{(-1+\mu )T} \Lambda (T)\Vert \partial _T X \Vert _{H^{N-3}} + \Lambda (T)^2 \Vert \partial _T u^0 \Vert _{H^{N-3}}\\&\quad + \varepsilon e^{(-1+\mu )T} \Lambda (T)\Vert \tau \partial _T u^b \Vert _{H^{N-3}} + \Lambda (T)^2.\end{aligned}\end{aligned}$$

To estimate $\partial _T E$ we use the rescaled continuity equation [1, Eq 10.16]:

$$\begin{aligned} \partial _T E= (3-N)E- X^a \nabla _a E+ \tau N^{-1} \nabla _a (N^2 j^a) - \tau ^2 {\tfrac{N}{3}} g_{ab} \underline{T}^{ab} - \tau ^2 N \Sigma _{ab} \underline{T}^{ab}. \end{aligned}$$

By Lemma 4.4, we obtain

$$\begin{aligned}\begin{aligned} |\tau |\Vert \partial _T E\Vert _{H^{N-3}}&\lesssim |\tau |\Vert E\Vert _{H^{N-2}}\big ( \Vert \widehat{N}\Vert _{H^{N-3}} + \Vert X\Vert _{H^{N-3}}\big ) + |\tau |^2 \Vert \jmath \Vert _{H^{N-2}}\\&\quad +|\tau |^3 \Vert \underline{T}\Vert _{H^{N-3}} \\&\lesssim \varepsilon e^{(-1+\mu )T}\Lambda (T) + \Lambda (T)^2.\end{aligned}\end{aligned}$$

To estimate $\partial _T \rho , \partial _T u^0, \partial _T u^a$ we use the equations of motion (2.7d) together with Lemma 5.9. We find,

5.9a $$\begin{aligned} \begin{aligned} |\tau |\Vert \partial _T \rho \Vert _{H^{N-3}}&\lesssim |\tau |\Vert \rho \Vert _{H^{N-3}} \Vert F_\rho \Vert _{H^{N-3}}+ |\tau |^2 \Vert u\Vert _{H^{N-3}} \Vert \rho \Vert _{H^{N-2}} \\  &\lesssim \varepsilon e^{\max \{-1+\mu ,-\lambda \}T} \Lambda (T) + \Lambda (T)^2,\end{aligned}\end{aligned}$$

and

5.9b $$\begin{aligned} \begin{aligned} \Vert \partial _T u^0\Vert _{H^{N-2}}&\lesssim \Vert F_{u^0} \Vert _{H^{N-2}}+ |\tau | \Vert u\Vert _{H^{N-2}} \Vert \nabla u^0\Vert _{H^{N-2}}\\&\lesssim \Lambda (T) + \Vert \Sigma \Vert _{H^{N-2}}^2, \\ |\tau |\Vert \partial _T u^a\Vert _{H^{N-2}}&\lesssim |\tau |\Vert F_{u^a} \Vert _{H^{N-2}}+ \Vert \widehat{N}\Vert _{H^{N-1}} + \tau ^2 \Vert u\Vert _{H^{N-1}}^2\\&\lesssim \Lambda (T) + \varepsilon ^2 e^{-(1+\nu )T}.\end{aligned}\end{aligned}$$

Putting all these estimates into (5.8) gives, for $2\le k \le N-3$,

$$\begin{aligned}\begin{aligned} |\tau |\Vert \partial _T \eta \Vert _{H^{k}}&\lesssim \varepsilon e^{(-1+\mu )T} \Lambda (T) + \Lambda (T)^2.\end{aligned}\end{aligned}$$

Next, and again using (2.6), we compute

$$\begin{aligned}\begin{aligned} \partial _T \jmath ^a&= (\partial _T \rho ) N u^0 u^a + (\partial _T N) \rho u^0 u^a + (\partial _T u^0) \rho N u^a + (\partial _T u^a) \rho N u^0,\end{aligned}\end{aligned}$$

So that

$$\begin{aligned} \begin{aligned} \Lambda (T)\Vert \partial _T \jmath ^a\Vert _{H^{N-3}}&\lesssim \varepsilon |\tau |\Vert u \Vert _{H^{N-3}}\Vert \partial _T \rho \Vert _{H^{N-3}} + \\  &\quad \varepsilon |\tau |\Vert \rho \Vert _{H^{N-3}}\Big (\Vert \partial _T N\Vert _{H^{N-3}} \Vert u \Vert _{H^{N-3}} + \Vert \partial _T u^0\Vert _{H^{N-3}} \Vert u \Vert _{H^{N-3}}\\  &\quad + \Vert \partial _T u^a \Vert _{H^{N-3}} \Big ) \\  &\lesssim \varepsilon ^2 \Lambda (T) +\varepsilon ^4 e^{-(1+\nu )T}. \end{aligned} \end{aligned}$$

Finally, from (2.6) we calculate (written schematically)

$$\begin{aligned}\begin{aligned} \partial _T S&= (\partial _T\rho )\big ( ( u^0 X^a+ \tau u^a )^2 + \tfrac{1}{2} g\big ) + \tfrac{1}{2}\rho (\partial _Tg) + \rho \partial _T g \cdot ( u^0 X^c+ \tau u^c )^2 \\  &\quad + \rho \partial _T( u^0 X^c+ \tau u^c)\cdot (u^0 X^d+ \tau u^d).\end{aligned}\end{aligned}$$

Using Lemma 5.6 and (5.9) we have

$$\begin{aligned}\begin{aligned} |\tau | \Vert \partial _T S\Vert _{H^{N-3}}&\lesssim |\tau |(\Vert \rho \Vert _{H^{N-3}} \Vert F_\rho \Vert _{H^{N-3}}+ |\tau |\Vert u\Vert _{H^{N-3}} \Vert \rho \Vert _{H^{N-2}})\\&\quad + |\tau |\Vert \rho \Vert _{H^{N-3}} \times \big ( \text {h.o.t.}\big ) \\  &\lesssim |\tau |\Vert \rho \Vert _{H^{N-2}} .\end{aligned}\end{aligned}$$

$$\square $$

### Second Commutator Estimates

We are now in a position to compute various commutator estimates which are required in the later energy estimates. We let *V* be a symmetric (0, 2)-tensor on *M* unless otherwise specified. The first lemma looks at the commutator between the dust derivative $\partial _{\textbf{u}}$ with other first-order differential operators.

#### Lemma 5.13

For $0\le k \le N-2$, we have

$$\begin{aligned}\begin{aligned} \Vert [\partial _{\textbf{u}}, \partial _T] V\Vert _{H^k}&\lesssim \varepsilon e^{(-1+\mu ) T} \Vert V\Vert _{H^{k+1}} + \varepsilon e^{(-1+\mu )T}\Vert \partial _T V\Vert _{H^k}, \\ \Vert [\partial _{\textbf{u}}, \hat{\nabla }] V \Vert _{H^k}&\lesssim \varepsilon e^{(-1+\mu ) T} \Vert V\Vert _{H^{k+1}} +\Lambda (T)\Vert \partial _T V \Vert _{H^k} , \\ \Vert [\partial _{\textbf{u}}, \nabla ] V \Vert _{H^{k}}&\lesssim \varepsilon e^{\max \{-1+\mu , -\lambda \} T} \Vert V\Vert _{H^{k+1}} +\Lambda (T)\Vert \partial _T V\Vert _{H^{k}}.\end{aligned} \end{aligned}$$

#### Proof

A computation gives

5.10 $$\begin{aligned} \begin{aligned} [\partial _{\textbf{u}}, \partial _T] V_{i j}&= -\tau u^a \hat{\nabla }_a V_{i j} + \tau \partial _T u^a \cdot \hat{\nabla }_a V_{i j} - \partial _T \hat{u}^0\cdot \partial _T V_{i j} ,\\ [\partial _{\textbf{u}}, \hat{\nabla }_b] V_{i j}&= -\tau u^a [\hat{\nabla }_a, \hat{\nabla }_b]V_{i j} -\hat{\nabla }_b \hat{u}^0\cdot \partial _T V_{i j} + \tau \hat{\nabla }_b u^a \cdot \hat{\nabla }_a V_{ij},\\ [\partial _{\textbf{u}}, \nabla _b] V_{i j}&= u^0[\partial _T, \nabla _b]V_{i j}-\tau u^c[\hat{\nabla }_c,\nabla _b]V_{i j}-\nabla _b\hat{u}^0\cdot \partial _T V_{i j} + \tau \nabla _b u^c\cdot \hat{\nabla }_c V_{i j}.\end{aligned} \end{aligned}$$

Thus, by (4.2) and (5.9), if $2\le k \le N-2$,

$$\begin{aligned}\begin{aligned} \Vert [\partial _{\textbf{u}}, \partial _T] V\Vert _{H^k}&\lesssim |\tau | \big (\Vert u \Vert _{H^k} +\Vert \partial _T u^a \Vert _{H^k} \big )\Vert \nabla V + \Upsilon *V\Vert _{H^{k}} + \Vert \partial _T u^0\Vert _{H^k} \Vert \partial _T V\Vert _{H^k} \\  &\lesssim \varepsilon e^{(-1+\mu ) T}\big ( \Vert V\Vert _{H^{k+1}} + \Vert g-\gamma \Vert _{H^{k+1}}\Vert V\Vert _{H^{k}}\big ) + \varepsilon e^{(-1+\mu )T}\Vert \partial _T V\Vert _{H^k}.\end{aligned} \end{aligned}$$

The estimates for $k=0,1$ follow in the same way.

The other two estimates follow in a similar way. Note that for $[\partial _{\textbf{u}}, \nabla$] we use Lemma 5.2, and Eqs. (5.1), (5.2) and (5.9). $\square$

The next lemma in this subsection investigates the commutator between the second-order operator $\mathcal {L}_{g, \gamma }$ and other first-order operators.

#### Lemma 5.14

The following estimates hold:

$$\begin{aligned}\begin{aligned} \Vert [\partial _{\textbf{u}}, \mathcal {L}_{g, \gamma }] V\Vert _{H^k}&\lesssim \varepsilon e^{\max \{-1+\mu ,-\lambda \} T}\Vert V \Vert _{H^{k+2}}\\&\quad + \Lambda (T)\Vert \partial _T V\Vert _{H^{k+1}}, \qquad&0\le k \le N-3, \\ \Vert [\hat{\nabla }_m,\mathcal {L}_{g, \gamma }]V \Vert _{H^{k}}&\lesssim \varepsilon e^{-\lambda T} \Vert V \Vert _{H^{k+2}}+ \Vert V\Vert _{H^{k}}, \qquad&0\le k \le N-2, \\ \Vert [\partial _T, \mathcal {L}_{g, \gamma }] V\Vert _{H^k}&\lesssim \varepsilon e^{-\lambda T} \Vert V \Vert _{H^{k+2}},\qquad&0\le k \le N-2.\end{aligned} \end{aligned}$$

Also, for $k,s\in \mathbb {Z}$ such that $0 \le k \le N-2$, $s \ge 1$ and $2(s-1)+k \le N-1$, we have

$$\begin{aligned}\begin{aligned} \Vert [\partial _T, \mathcal {L}_{g, \gamma }^s] V\Vert _{H^k}&\lesssim \varepsilon e^{-\lambda T}\Vert V \Vert _{H^{k+2+2(s-1)}} .\end{aligned}\end{aligned}$$

#### Proof

A calculation yields

5.11 $$\begin{aligned} {[}\partial _{\textbf{u}}, \mathcal {L}_{g, \gamma }] V_{ij}&= -\big (\partial _{\textbf{u}}g^{ab}\big )\hat{\nabla }_a \hat{\nabla }_b V_{ij}+\hat{\Delta }_{g, \gamma }\hat{u}^0\cdot \partial _T V_{ij}+2g^{ab} \hat{\nabla }_a \hat{u}^0\hat{\nabla }_b \partial _T V_{ij} \nonumber \\&\quad +\tau u^c g^{ab} \big (\text {Riem}[\gamma ]^k _{bac}\hat{\nabla }_k V_{ij} +4 \text {Riem}[\gamma ]^k _{(i|ac} \hat{\nabla }_b V_{k|j)}\nonumber \\&\quad + 2\hat{\nabla }_a \text {Riem}[\gamma ]^k _{(i|bc} \cdot V_{k|j)}\big ) -2\tau g^{ab} \hat{\nabla }_a u^c \hat{\nabla }_b\hat{\nabla }_c V_{ij}\nonumber \\&\quad - \tau \hat{\Delta }_{g, \gamma }u^c \cdot \hat{\nabla }_c V_{ij} + 2\tau u^c \hat{\nabla }_c \text {Riem}[\gamma ]_{iajb}\cdot V^{ab}. \end{aligned}$$

It is useful to write this schematically, using that $\text {Riem}[\gamma ]$ and its derivatives are bounded by constants

$$\begin{aligned}\begin{aligned} [\partial _{\textbf{u}}, \mathcal {L}_{g, \gamma }] V&= (\partial _{\textbf{u}}g^{-1}) *\hat{\nabla }^2 V + \hat{\nabla }^2 \hat{u}^0*\partial _T V+ \hat{\nabla }u^0 *\hat{\nabla }\partial _T V\\&\quad +\tau \big ( u^c *(V + \hat{\nabla }V ) + \hat{\nabla }u^c *\hat{\nabla }^2 V + \hat{\nabla }^2 u^c *\hat{\nabla }V \big ).\end{aligned}\end{aligned}$$

If $2\le k \le N-3$ then by elliptic regularity of Lemma 3.7 and the commutator estimates in Lemma 5.2,

5.12 $$\begin{aligned} \begin{aligned} \Vert [\partial _{\textbf{u}}, \mathcal {L}_{g, \gamma }] V\Vert _{H^k}&\lesssim \Vert \partial _{\textbf{u}}g\Vert _{H^k}\Vert V \Vert _{H^{k+2}} + \Vert \hat{u}^0\Vert _{H^{k+2}}\Vert \partial _T V\Vert _{H^{k+1}} \\&\quad + |\tau | \Vert u\Vert _{H^{k+2}}\Vert V\Vert _{H^{k+2}}.\end{aligned} \end{aligned}$$

The conclusion then holds by (4.2) and by estimating $\partial _{\textbf{u}}g$ using Corollary 5.7 . The estimates when $k=0,1$ follow in a similar way.

Next we compute the identity

5.13 $$\begin{aligned} \begin{aligned} [\mathcal {L}_{g, \gamma }, \hat{\nabla }_m]V_{ij}&= \hat{\nabla }_m g^{ab} \cdot \hat{\nabla }_a\hat{\nabla }_b V_{ij} - g^{ab} [\hat{\nabla }_a\hat{\nabla }_b, \hat{\nabla }_m]V_{ij},\end{aligned}\end{aligned}$$

where we are thinking of the *m* index as not being free (i.e. contracted with a factor of the shift $X^m$). Since the commutator involving only $\hat{\nabla }$ will just generate background Riemann curvature components the required estimate follows straightforwardly. We note also that (5.13) and the elliptic regularity of Lemma 3.7 imply, for $s \in \mathbb {Z}$ such that $1\le s \le N/2$,

5.14 $$\begin{aligned} \begin{aligned} \Vert [\mathcal {L}_{g, \gamma }^s, \hat{\nabla }_m]V \Vert _{L^2}&\lesssim \Vert [\mathcal {L}_{g, \gamma }, \hat{\nabla }]\mathcal {L}_{g, \gamma }^{s-1} V\Vert _{L^2} + \cdots + \Vert [\mathcal {L}_{g, \gamma },\hat{\nabla }]V\Vert _{H^{2(s-1)}} \\  &\lesssim \Vert g-\gamma \Vert _{H^{\max \{3, 2s-1\}}}\big (\Vert V \Vert _{H^{2s}} + \Vert g-\gamma \Vert _{H^{2s}}\Vert V\Vert _{H^{2s-1}}\big )\\&\quad + \Vert V\Vert _{H^{2(s-1)}} \\  &\lesssim \varepsilon e^{-\lambda T} \Vert V \Vert _{H^{2s}} + \Vert V\Vert _{H^{2(s-1)}}.\end{aligned} \nonumber \\ \end{aligned}$$

Finally we compute

$$\begin{aligned}\begin{aligned} [\partial _T, \mathcal {L}_{g, \gamma }]V_{ij}&= - [\partial _T, \hat{\Delta }_{g, \gamma }]V_{ij} = -(\partial _T g^{ab}) \hat{\nabla }_a \hat{\nabla }_b V_{ij}.\end{aligned}\end{aligned}$$

Using Lemma 5.6 to estimate $\partial _T g$ this gives, for $2\le k \le N-2$,

$$\begin{aligned} \Vert [\partial _T, \mathcal {L}_{g, \gamma }] V\Vert _{H^k}&\lesssim \Vert \partial _T h\Vert _{H^k} \big (\Vert \nabla (\nabla V +\Upsilon *V)\Vert _{H^{k}} + \Vert \Upsilon (\nabla V+\Upsilon *V) \Vert _{H^{k}} \big )\\&\lesssim \varepsilon e^{-\lambda T} \Vert V \Vert _{H^{k+2}}. \end{aligned}$$

A similar argument holds for the cases $k=0,1$. At higher order, we obtain the identity

5.15 $$\begin{aligned} \begin{aligned} [\partial _T, \mathcal {L}_{g, \gamma }^s] V_{ij}&= -\sum _{1\le i \le s} \mathcal {L}_{g, \gamma }^{i-1} (\partial _T g^{a_i b_i})\cdot \hat{\nabla }_{a_i}\hat{\nabla }_{b_i} \big ( \mathcal {L}_{g, \gamma }^{s-i} (V_{ij})\big ). \end{aligned} \end{aligned}$$

This can be estimated, for $2\le k \le N-2$, by

5.16 $$\begin{aligned} \begin{aligned} \Vert [\partial _T, \mathcal {L}_{g, \gamma }^s] V\Vert _{H^k}&\lesssim \Vert \partial _T h\Vert _{H^{2(s-1)+k}} \big (\Vert V \Vert _{H^{k+2+2(s-1)}} \\&\quad + \Vert g -\gamma \Vert _{H^{k+2}}\Vert V\Vert _{H^{k+1+2(s-1)}}\big ). \end{aligned}\end{aligned}$$

The cases $k=0,1$ are treated in a similar way and the conclusion follows from Lemma 5.6. $\square$

The next corollary extends the commutator estimates of the previous lemma to higher-orders of $\mathcal {L}_{g, \gamma }^s$, $s \in \mathbb {Z}_{\ge 1}$. Note that the $L^2$ estimate that appears in the statement will be typically applied with $V=h$, while the lower-order $H^1$ estimate will be primarily used later on with $V = \Sigma$.

#### Corollary 5.15

We have

$$\begin{aligned}\begin{aligned} \Vert [ \partial _{\textbf{u}}, \mathcal {L}_{g, \gamma }^s] V \Vert _{L^2}&\lesssim \varepsilon e^{\max \{-1+\mu ,-\lambda \} T}\Vert V\Vert _{H^{2s}}\\&\quad + \Lambda (T)\Vert \partial _T V\Vert _{H^{2s-1}} ,&\quad 1\le s \le \ell , \\ \Vert [ \partial _{\textbf{u}}, \mathcal {L}_{g, \gamma }^{s-1}] V \Vert _{H^1}&\lesssim \varepsilon e^{\max \{-1+\mu ,-\lambda \} T}\Vert V\Vert _{H^{2s-1}}\\&\quad + \Lambda (T)\Vert \partial _T V\Vert _{H^{2s-2}} ,&\quad 2\le s \le \ell .\end{aligned}\end{aligned}$$

#### Proof

By Lemma 5.14,

$$\begin{aligned}\begin{aligned} \Vert [ \partial _{\textbf{u}}, \mathcal {L}_{g, \gamma }^s] V \Vert _{L^2}&\lesssim \Vert [ \partial _{\textbf{u}}, \mathcal {L}_{g, \gamma }] \mathcal {L}_{g, \gamma }^{s-1}V \Vert _{L^2} + \Vert [ \partial _{\textbf{u}}, \mathcal {L}_{g, \gamma }] \mathcal {L}_{g, \gamma }^{s-2}V \Vert _{H^2}\\&\quad + \cdots + \Vert [ \partial _{\textbf{u}}, \mathcal {L}_{g, \gamma }] V \Vert _{H^{2(s-1)}} \\&\lesssim \varepsilon e^{\max \{-1+\mu ,-\lambda \} T}\Vert V\Vert _{H^{2s}} +\Lambda (T)\Vert \partial _T V\Vert _{H^{2s-1}}\\&\quad + \Lambda (T) \sum _{p=0}^{s-2}\Vert [\partial _T, \mathcal {L}_{g, \gamma }^{s-1-p}]V\Vert _{H^{1+2p}}.\end{aligned}\end{aligned}$$

The conclusion then easily follows and the second estimate follows in the same way. $\square$

#### Corollary 5.16

$$\begin{aligned}\begin{aligned} \Vert \partial _{\textbf{u}}\mathcal {L}_{g, \gamma }^{\ell -1}(\Sigma )\Vert _{L^2}&\lesssim (E^g_{N-1})^{1/2} +\Lambda (T),\\ \Vert \partial _{\textbf{u}}\mathcal {L}_{g, \gamma }^{\ell -1}(\Sigma )\Vert _{H^1}&\lesssim \Vert \Sigma \Vert _{H^{N-1}}+ \Vert g-\gamma \Vert _{H^{N}} + \Lambda (T).\end{aligned} \end{aligned}$$

#### Proof

By the $\partial _{\textbf{u}}\Sigma , \partial _T \Sigma$ estimates of Lemma 5.6, Corollary 5.7, and the previous commutator estimate of corollary 5.15

$$\begin{aligned} \Vert \partial _{\textbf{u}}\mathcal {L}_{g, \gamma }^{\ell -1}(\Sigma )\Vert _{L^2}&\lesssim \Vert \partial _{\textbf{u}}\Sigma \Vert _{H^{N-3}} +\Vert [\partial _{\textbf{u}},\mathcal {L}_{g, \gamma }^{\ell -1}](\Sigma )\Vert _{L^2}\\&\lesssim \Vert \Sigma \Vert _{H^{N-2}}+ \Vert g-\gamma \Vert _{H^{N-1}} +\Lambda (T). \end{aligned}$$

The conclusion then follows by the coercive estimate of Lemma 4.5. Similarly,

5.17 $$\begin{aligned} \begin{aligned} \Vert \partial _{\textbf{u}}\mathcal {L}_{g, \gamma }^{\ell -1} \Sigma \Vert _{H^1}&\lesssim \Vert \Sigma \Vert _{H^{N-1}}+ \Vert g-\gamma \Vert _{H^{N}} + \Lambda (T) + \varepsilon e^{\max \{-1+\mu ,-\lambda \} T}\Vert \Sigma \Vert _{H^{N-2}} \\  &\quad + \Lambda (T)\Vert \partial _T \Sigma \Vert _{H^{N-3}} \\&\lesssim \Vert \Sigma \Vert _{H^{N-1}}+ \Vert g-\gamma \Vert _{H^{N}} + \Lambda (T).\end{aligned}\end{aligned}$$

$$\square $$

#### Remark 5.17

Frequently in our energy estimates we will need to study the term $\Vert \partial _{\textbf{u}}\mathcal {L}_{g, \gamma }^{\ell -1}(\Sigma )\Vert _{H^1}$ appearing in the previous Corollary. However, by looking at the estimate derived in Corollary 5.16, we see that we cannot apply Lemma 4.5 to the top-order Sobolev norms $\Vert \Sigma \Vert _{H^{N-1}}$ and $\Vert g-\gamma \Vert _{H^{N}}$. To estimate these terms by the geometric energy $E^g_{tot}$ we will instead need to use the auxiliary elliptic estimates established in Sect. 6.

We conclude this subsection with a commutator estimate that plays an important role in the proof of the lapse estimate appearing in Proposition 7.3.

#### Lemma 5.18

Let $\Delta :=g^{ab}\nabla _a\nabla _b$. Then for $0\le k \le N-3$,

$$\begin{aligned}\begin{aligned} \Vert [\Delta , \partial _{\textbf{u}}]V\Vert _{H^k}&\lesssim \Lambda (T) \Vert \partial _T V\Vert _{H^{k+1}} + \varepsilon e^{\max \{-1+\mu ,-\lambda \} T}\Vert V\Vert _{H^{k+2}}.\end{aligned}\end{aligned}$$

#### Proof

We compute

$$\begin{aligned}\begin{aligned} [\Delta , \partial _{\textbf{u}}]V_{ij}&= \Delta \hat{u}^0\cdot \partial _T V_{ij}+2\nabla ^a\hat{u}^0\nabla _a(\partial _T V_{ij}) - u^0 \partial _T g^{ab} \cdot \nabla _a\nabla _v V_{ij}\\&\quad + u^0 g^{ab}[\nabla _a\nabla _b, \partial _T]V_{ij} + \tau \big ( -\Delta u^c \cdot \hat{\nabla }_c V_{ij} - 2 \nabla _a u^c \nabla ^a \hat{\nabla }_c V_{ij}+ u^c\hat{\nabla }_c g^{ab}\\  &\quad \cdot \nabla _a \nabla _b V_{ij} \big ) + \tau u^c g^{ab} [\hat{\nabla }_c, \nabla _a \nabla _b]V_{ij}.\end{aligned}\end{aligned}$$

Let $2\le k \le N-3$. From Lemma 5.2 and (5.3) we find

$$\begin{aligned}\begin{aligned} \Vert [\Delta , \partial _{\textbf{u}}]V\Vert _{H^k}&\lesssim \Vert \nabla \hat{u}^0\Vert _{H^{k+1}}\Vert \partial _T V\Vert _{H^{k+1}} + \Vert \partial _T h \Vert _{H^{k}} \Vert V\Vert _{H^{k+2}} \\  &\quad + \Vert \partial _T \Gamma \Vert _{H^{k+1}}\Vert V\Vert _{H^{k+1}} + |\tau | \Vert u \Vert _{H^{k+2}}\Vert V\Vert _{H^{k+2}}.\end{aligned}\end{aligned}$$

The cases $k=0,1$ follow in the same way. The conclusion follows using Lemma 5.6, (4.2) and (5.2). $\square$

### Second Geometric Components Estimates

We now reach the final subsection of Sect. 5. The first lemma is an analogue of Lemma 4.5 for our top-order $\partial _{\textbf{u}}-$*boosted* geometric energy. The proof follows those in [1, Lemma 19] and [3, Lemma 7.2].

#### Lemma 5.19

Let $s\in \mathbb {Z}$ such that $1\le s \le \ell$. There is a $\delta >0$ and a constant $C>0$ such that for $(g,\Sigma )\in B_{\delta }((\gamma ,0))$ the inequality

$$\begin{aligned}\begin{aligned} (\partial _{\textbf{u}}\mathcal {L}_{g, \gamma }^s h, \partial _{\textbf{u}}\mathcal {L}_{g, \gamma }^s h)_{L^2_{g,\gamma }} + |(\partial _{\textbf{u}}\mathcal {L}_{g, \gamma }^s v, \partial _{\textbf{u}}\mathcal {L}_{g, \gamma }^{s-1}v)_{L^2_{g,\gamma }}|&\le C E^g_{\partial _{\textbf{u}}, 2\ell } \end{aligned} \end{aligned}$$

holds. Furthermore $$E^g_{\partial _{\textbf{u}}, 2\ell } \ge 0$$.

#### Proof

Recall $E^g_{\partial _{\textbf{u}}, 2\ell }$ from Definition 3.11. We first note that $E^g_{\partial _{\textbf{u}}, 2\ell }|_{(h,v)=(\gamma ,0)} =0$. Next, we see that $(\gamma , 0)$ is a critical point of $E^g_{\partial _{\textbf{u}}, 2\ell }$ since the first derivative vanishes. Considering then the second derivative of this energy at $(\gamma ,0)$, we see that the Hessian takes the form

$$\begin{aligned}\begin{aligned} D^2&(\mathcal {E}^g_{\partial _{\textbf{u}}, 2s}+c_E \Gamma ^g_{\partial _{\textbf{u}}, 2s})((h,k),(h,k)) \\&= 9 (\partial _{\textbf{u}}\mathcal {L}_{\gamma ,\gamma }^s h, \partial _{\textbf{u}}\mathcal {L}_{\gamma ,\gamma }^s h)_{L^2_{g,\gamma }}+ (\partial _{\textbf{u}}\mathcal {L}_{\gamma ,\gamma }^s k, \partial _{\textbf{u}}\mathcal {L}_{\gamma ,\gamma }^{s-1} k)_{L^2_{g,\gamma }}\\&\quad + c_E(\partial _{\textbf{u}}\mathcal {L}_{\gamma ,\gamma }^{s-1} k, \partial _{\textbf{u}}\mathcal {L}_{\gamma ,\gamma }^s h)_{L^2_{g,\gamma }}. \end{aligned} \end{aligned}$$

We claim that the Hessian is non-negative. By expanding in terms of the eigentensors of $\mathcal {L}_{\gamma , \gamma }$, we are left with terms of the type

$$\begin{aligned}\begin{aligned} \lambda ^{2s-1} \Big ( 9 \lambda (\partial _{\textbf{u}}P_\lambda h, \partial _{\textbf{u}}P_\lambda h)_{L^2_{g,\gamma }}+ (\partial _{\textbf{u}}P_\lambda k, \partial _{\textbf{u}}P_\lambda k)_{L^2_{g,\gamma }} + c_E(\partial _{\textbf{u}}P_\lambda k, \partial _{\textbf{u}}P_\lambda h)_{L^2_{g,\gamma }}\Big ),\end{aligned}\end{aligned}$$

where $P_\lambda$ denotes the projection operator onto the $\lambda$-eigenspace. The choice of $c_E$ ensures that the bracketed term is non-negative for the smallest eigenvalue $\lambda _0$, which in turn implies non-negativity for all eigenvalues. Thus we find

$$\begin{aligned}\begin{aligned} D^2 (\mathcal {E}^g_{\partial _{\textbf{u}}, 2s}+c_E \Gamma ^g_{\partial _{\textbf{u}}, 2s})((h,k),(h,k))\ge 0.\end{aligned}\end{aligned}$$

From this it follows that there is a constant $C = C(\lambda _0, \gamma )>0$ such that

$$\begin{aligned}\begin{aligned} \sum _{s=1}^\ell (\partial _{\textbf{u}}\mathcal {L}_{g, \gamma }^s h, \partial _{\textbf{u}}\mathcal {L}_{g, \gamma }^s h)_{L^2_{g,\gamma }} + |(\partial _{\textbf{u}}\mathcal {L}_{g, \gamma }^s k, \partial _{\textbf{u}}\mathcal {L}_{g, \gamma }^{s-1}k)_{L^2_{g,\gamma }}|&\le C E^g_{\partial _{\textbf{u}}, 2\ell }.\end{aligned}\end{aligned}$$

$$\square $$

The next lemma provides estimates for the Sobolev norms of $\partial _{\textbf{u}}h, \partial _{\textbf{u}}\Sigma$ in terms of the geometric energy $E^g_{tot}$. This is natural given that we have constructed the functional $E^g_{tot}$ to precisely control such Sobolev norms.

#### Lemma 5.20

(Dust derivatives of geometric variables at high-regularity) We have,

$$\begin{aligned}\begin{aligned} \Vert \partial _{\textbf{u}}h \Vert _{H^{N-1}}^2&\lesssim {E^g_{\partial _{\textbf{u}},N-1}}+\varepsilon ^2 e^{2\max \{-1+\mu ,-\lambda \} T}E^g_{N-1} + \Lambda (T)^4, \\ \Vert \partial _{\textbf{u}}\Sigma \Vert _{H^{N-2}}^2&\lesssim {E^g_{\partial _{\textbf{u}},N-1}}+ E^g_{N-1} + \varepsilon e^{\max \{-1+\mu ,-\lambda \} T}\\&\quad \times \big (\Vert \Sigma \Vert _{H^{N-1}}^2 + \Vert g-\gamma \Vert _{H^N}^2\big ) + \Lambda (T)^2.\end{aligned}\end{aligned}$$

#### Remark 5.21

Similar to Remark 5.17, we cannot apply Lemma 4.5 to the top-order Sobolev norms $\Vert \Sigma \Vert _{H^{N-1}}^2$ and $\Vert g-\gamma \Vert _{H^{N}}^2$. To estimate these terms by the geometric energy $E^g_{tot}$ we will instead need to use the auxiliary elliptic estimates of Sect. 6. It is also crucial in later analysis in Sect. 6 that these top-order norms above appear on the right hand side in Lemma 5.20 with a smallness factor of $\varepsilon$.

#### Proof

(Proof of Lemma 5.20) Recall that $N :=2\ell +1$. By the elliptic regularity of Lemma 3.7 and Lemma 5.19

$$\begin{aligned}\begin{aligned} \Vert \partial _{\textbf{u}}h \Vert _{H^{2\ell }}^2\lesssim \Vert \mathcal {L}_{g, \gamma }^\ell \partial _{\textbf{u}}h \Vert _{L^2}^2 \lesssim {E^g_{\partial _{\textbf{u}},N-1}}+ \Vert [ \partial _{\textbf{u}}, \mathcal {L}_{g, \gamma }^\ell ] h \Vert _{L^2}^2.\end{aligned}\end{aligned}$$

We control the commutator term using Corollary 5.15, finding

5.18 $$\begin{aligned} \begin{aligned} \Vert [ \partial _{\textbf{u}}, \mathcal {L}_{g, \gamma }^\ell ] h \Vert _{L^2}&\lesssim \varepsilon e^{\max \{-1+\mu ,-\lambda \} T}\Vert g-\gamma \Vert _{H^{N-1}} +\Lambda (T) \Vert \partial _T h\Vert _{H^{N-2}} \\&\lesssim \varepsilon e^{\max \{-1+\mu ,-\lambda \} T}(E^g_{N-1})^{1/2} + \Lambda (T)^2,\end{aligned}\end{aligned}$$

where in the final line we used Lemma 5.6 and the coercive estimate of Lemma 4.5.

Next, by elliptic regularity and Lemma 4.6, we have

5.19 $$\begin{aligned} \begin{aligned} \Vert \partial _{\textbf{u}}\Sigma \Vert _{H^{2\ell -1}}^2&\lesssim \Vert \mathcal {L}_{g, \gamma }^{\ell -1} \partial _{\textbf{u}}\Sigma \Vert _{H^1}^2 \\  &\lesssim \Vert \partial _{\textbf{u}}\mathcal {L}_{g, \gamma }^{\ell -1} \Sigma \Vert _{L^2} ^2 + \Vert [ \partial _{\textbf{u}}, \mathcal {L}_{g, \gamma }^{\ell -1}] \Sigma \Vert _{L^2}^2 + (\mathcal {L}_{g, \gamma }^{\ell -1} \partial _{\textbf{u}}\Sigma , \mathcal {L}_{g, \gamma }^\ell \partial _{\textbf{u}}\Sigma )_{L^2_{g,\gamma }}. \end{aligned}\nonumber \\ \end{aligned}$$

The first term on the RHS of (5.19) is treated by Corollary 5.16. For the commutator term in (5.19), we use Lemma 5.6 and Corollary 5.15 to find

5.20 $$\begin{aligned} \begin{aligned}&\Vert [ \partial _{\textbf{u}}, \mathcal {L}_{g, \gamma }^{\ell -1}] \Sigma \Vert _{L^2} \\&\quad \lesssim \varepsilon e^{\max \{-1+\mu ,-\lambda \} T}\Vert \Sigma \Vert _{H^{N-3}} + \Lambda (T) \Vert \partial _T \Sigma \Vert _{H^{N-4}} \\  &\quad \lesssim \varepsilon e^{\max \{-1+\mu ,-\lambda \} T}(E^g_{N-2})^{1/2} + \Lambda (T)^2.\end{aligned}\end{aligned}$$

Considering next the final term in (5.19), we write it as

$$\begin{aligned}&(\mathcal {L}_{g, \gamma }^{\ell -1} \partial _{\textbf{u}}\Sigma , \mathcal {L}_{g, \gamma }^\ell \partial _{\textbf{u}}\Sigma )_{L^2_{g,\gamma }} \\&\quad = (\partial _{\textbf{u}}\mathcal {L}_{g, \gamma }^{\ell -1} \Sigma , \partial _{\textbf{u}}\mathcal {L}_{g, \gamma }^\ell \Sigma )_{L^2_{g,\gamma }} + ([\mathcal {L}_{g, \gamma }^{\ell -1}, \partial _{\textbf{u}}] \Sigma , \partial _{\textbf{u}}\mathcal {L}_{g, \gamma }^\ell \Sigma )_{L^2_{g,\gamma }} \\  &\qquad + (\partial _{\textbf{u}}\mathcal {L}_{g, \gamma }^{\ell -1} \Sigma , [\partial _{\textbf{u}},\mathcal {L}_{g, \gamma }^\ell ]\Sigma )_{L^2_{g,\gamma }} + ([\mathcal {L}_{g, \gamma }^{\ell -1}, \partial _{\textbf{u}}] \Sigma , [\partial _{\textbf{u}}, \mathcal {L}_{g, \gamma }^\ell ]\Sigma )_{L^2_{g,\gamma }} \\&\quad =: E_1 + E_2 + E_3 + E_4. \end{aligned}$$

From Lemma 5.19, $|E_1| \lesssim {E^g_{\partial _{\textbf{u}},N-1}}$. To estimate $E_2$ we need to integrate by parts one of the derivatives appearing in $\partial _{\textbf{u}}\mathcal {L}_{g, \gamma }^\ell \Sigma$. Using (5.6a) we find

$$\begin{aligned} E_2&= (g^{ab} \hat{\nabla }_a [\mathcal {L}_{g, \gamma }^{\ell -1}, \partial _{\textbf{u}}] \Sigma , \hat{\nabla }_b \partial _{\textbf{u}}\mathcal {L}_{g, \gamma }^{\ell -1} \Sigma )_{L^2_{g,\gamma }} \\&\quad - 2 ([\mathcal {L}_{g, \gamma }^{\ell -1}, \partial _{\textbf{u}}] \Sigma , \text {Riem}[\gamma ]\circ \partial _{\textbf{u}}\mathcal {L}_{g, \gamma }^{\ell -1} \Sigma )_{L^2_{g,\gamma }} \\  &\quad + ([\mathcal {L}_{g, \gamma }^{\ell -1}, \partial _{\textbf{u}}] \Sigma , [\partial _{\textbf{u}}, \mathcal {L}_{g, \gamma }] \mathcal {L}_{g, \gamma }^{\ell -1} \Sigma )_{L^2_{g,\gamma }} .\end{aligned}$$

By the commutator estimates of Lemma 5.14, and Lemma 5.6, we have

5.21 $$\begin{aligned} \begin{aligned} \Vert [\partial _{\textbf{u}},\mathcal {L}_{g, \gamma }]\mathcal {L}_{g, \gamma }^{\ell -1}\Sigma \Vert _{L^2}&\lesssim \varepsilon e^{\max \{-1+\mu ,-\lambda \} T}\Vert \Sigma \Vert _{H^{N-1}}+ \Lambda (T)\Vert \partial _T \Sigma \Vert _{H^{N-2}} \\  &\lesssim \varepsilon e^{\max \{-1+\mu ,-\lambda \} T}\big (\Vert \Sigma \Vert _{H^{N-1}} + \Vert g-\gamma \Vert _{H^N}\big ) + \Lambda (T)^2.\end{aligned}\nonumber \\ \end{aligned}$$

Using this, together with (5.20) and Corollary 5.16, gives

$$\begin{aligned}\begin{aligned} \big |E_2 \big |&\lesssim \Vert [\partial _{\textbf{u}}, \mathcal {L}_{g, \gamma }^{\ell -1}] \Sigma \Vert _{H^1} \Big (\Vert \partial _{\textbf{u}}\mathcal {L}_{g, \gamma }^{\ell -1}\Sigma \Vert _{H^1} + \Vert [\partial _{\textbf{u}},\mathcal {L}_{g, \gamma }]\mathcal {L}_{g, \gamma }^{\ell -1}\Sigma \Vert _{L^2}\Big ) \\&\lesssim \varepsilon e^{\max \{-1+\mu ,-\lambda \} T}\big (\Vert \Sigma \Vert _{H^{N-1}}^2 + \Vert g-\gamma \Vert _{H^N}^2\big ) + \Lambda (T)^2 .\end{aligned}\end{aligned}$$

The terms $E_3, E_4$ are similarly estimated and inserting all these estimates into (5.19) gives the required result. $\square$

We end this subsection with two Lemmas concerning the geometric source terms $F_h$ and $F_v$.

#### Lemma 5.22

(Time derivative of geometric source terms) We have

$$\begin{aligned}\begin{aligned} \Vert \partial _T F_h \Vert _{H^{N-2}} + \Vert \partial _T F_v \Vert _{H^{N-3}}&\lesssim E^g_{N-1}+ \Lambda (T).\end{aligned} \end{aligned}$$

#### Proof

Let $2\le k \le N-2$. Taking a time derivative of $F_h$ as given in Definition 2.8 we see that

$$\begin{aligned}\begin{aligned} \Vert \partial _T F_h\Vert _{H^k}&\lesssim \Vert \partial _T N \Vert _{H^k} + \big (\Vert \widehat{N}\Vert _{H^k} + \Vert X\Vert _{H^{k+1}} + \Vert g-\gamma \Vert _{H^{k+1}} \Vert X \Vert _{H^k} \big ) \Vert \partial _T h \Vert _{H^k} \\  &+\Vert g-\gamma \Vert _{H^k} \big ( \Vert \partial _T X \Vert _{H^{k+1}} + \Vert g-\gamma \Vert _{H^{k+1}}\Vert \partial _T X \Vert _{H^{k}}\big ).\end{aligned}\end{aligned}$$

The first estimate then follows by applying Lemma 5.6.

Next, let $2\le k \le N-3$. We take a time derivative of $F_v$ which gives

$$\begin{aligned}\begin{aligned} \partial _T F_v&= \partial _T \nabla _i \nabla _j N + \partial _T N \cdot \big ( 2 \Sigma _{ic} \Sigma ^c_j -\tfrac{1}{3} g_{ij}- \Sigma _{ij} + \tau S_{ij}\big ) + 2 N \partial _T(\Sigma _{ic} \Sigma ^c_j)\\&\quad -\tfrac{1}{3}\widehat{N}\partial _T g_{ij} - \widehat{N}\partial _T \Sigma _{ij} - \tau N S_{ij} + \tau N \partial _T S_{ij}\\&\quad - \partial _T\big ( v_{im}\hat{\nabla }_j X^m + v_{mj}\hat{\nabla }_i X^m\big ).\end{aligned}\end{aligned}$$

For the first term we use the commutator identity from (5.3) and recall that the lapse is a scalar. We obtain

$$\begin{aligned}\begin{aligned} \Vert \partial _T F_v \Vert _{H^k}&\lesssim \Vert \partial _T N\Vert _{H^{k+2}} + \Vert [\partial _T, \nabla ]\nabla N\Vert _{H^k} + |\tau | \big ( \Vert S\Vert _{H^k} + \Vert \partial _T S\Vert _{H^k} \big ) \\  &\quad + \Vert \Sigma \Vert _{H^k}^2 + \Vert \partial _T \Sigma \Vert _{H^k}^2 + \Vert \partial _T g\Vert _{H^k}^2 + \Lambda (T)^2.\end{aligned}\end{aligned}$$

The conclusion then follows by Lemma 5.6 and the matter estimates in Lemma 4.4 and Lemma 5.12. $\square$

#### Corollary 5.23

(Dust derivative of geometric source terms) We have

$$\begin{aligned}\begin{aligned} \Vert \partial _{\textbf{u}}F_h \Vert _{H^{N-2}} + \Vert \partial _{\textbf{u}}F_v\Vert _{H^{N-3}}&\lesssim E^g_{N-1} + \varepsilon e^{(-1+\mu )T}\big ( \Vert g-\gamma \Vert _{H^N}^2 + \Vert \Sigma \Vert _{H^{N-1}}^2 \big )+ \Lambda (T), \\ \Vert \partial _{\textbf{u}}F_h \Vert _{H^{2}}&\lesssim E^g_{N-1} + \Lambda (T).\end{aligned}\end{aligned}$$

#### Proof

The estimates follow by expanding out $\partial _{\textbf{u}}= u^0 \partial _T - \tau u^c \hat{\nabla }_c$ and using the $F_h, F_v$ estimates in Lemma 5.5 and the $\partial _T F_h, \partial _T F_v$ estimates from Lemma 5.22. $\square$

#### Remark 5.24

Since we are dealing with geometric variables in the above corollary, and not matter variables, we roughly estimated the dust derivatives as $\partial _{\textbf{u}}\sim \partial _T + \tau u^c *\nabla$. Note that doing so introduced the top-order Sobolev norms $\Vert \Sigma \Vert _{H^{N-1}}^2$ and $\Vert g-\gamma \Vert _{H^{N}}^2$. Crucially for later analysis in Corollary 6.4, however, is that these top-order norms appear with a coefficient of $\varepsilon$.

## Elliptic Estimate

In this section we prove an auxiliary elliptic estimate which allows us to control the top-order Sobolev norms of *g* and $\Sigma$ in terms of the geometric energy functional $E^g_{tot}$. Recall only the lower-order Sobolev norms are controlled using Lemma 4.5, and so a new idea is indeed needed to cover the top-order of regularity. We also remind the reader that the geometric energy $E^g_{tot}$ will eventually fulfill a strong decay estimate enabled by the inclusion of certain correction terms.

The main result of the section, Corollary 6.4, achieves the goal of the previous paragraph. To prove this corollary, we take the first-order equations of motion for $\partial _T h, \partial _T \Sigma$ appearing in (2.8) and convert them into second-order equations involving a perturbed wave operator $\mathcal {W}$ and the $\partial _{\textbf{u}}$ derivatives. We find, very schematically, that

6.1 $$\begin{aligned} \mathcal {L}_{g, \gamma }h \sim \mathcal {W}(h) + \partial _{\textbf{u}}\partial _T (h) + \tau u^a \hat{\nabla }_a\partial _{\textbf{u}}h. \end{aligned}$$

By elliptic regularity for $\mathcal {L}_{g, \gamma }$, we can then prove an estimate on $\Vert g-\gamma \Vert _{H^{N}} \sim \Vert \mathcal {L}_{g, \gamma }h \Vert _{H^{N-2}}$ by estimating the RHS of (6.1), see Proposition 6.3. A similar idea holds also for $\Sigma$. We note that this idea, albeit for a different gauge, was first introduced by Hadžić and Speck in [16].

### Definition 6.1

(Operators $\mathcal {W},\mathcal {H}$) Define the operators

$$\begin{aligned}\begin{aligned} \mathcal {W}&:=\partial _T\partial _T-X^aX^b\hat{\nabla }_a \hat{\nabla }_b + N^2 \mathcal {L}_{g, \gamma }, \\ \mathcal {H}&:=N^2 \mathcal {L}_{g, \gamma }- X^aX^b\hat{\nabla }_a \hat{\nabla }_b - \tau ^2 \frac{u^a u^b}{(u^0)^2} \hat{\nabla }_b \hat{\nabla }_a ,\end{aligned}\end{aligned}$$

which act on symmetric (0, 2)-tensors.

Due to the sign convention on $\mathcal {L}_{g, \gamma }$, see Definition 3.2, one can think of $\mathcal {W}$ as being a kind of perturbed wave operator.

### Lemma 6.2

(Wave equations for *h*, *v*) The differential Eq. (2.8) for $h=g-\gamma$ and $v = 6 \Sigma$ imply

$$\begin{aligned} \mathcal {W}(h)= F_1, \qquad \mathcal {W}(v)= F_2, \end{aligned}$$

where,

$$\begin{aligned}\begin{aligned} \Vert F_1 \Vert _{H^{N-2}}^2 + \Vert F_2 \Vert _{H^{N-3}}^2&\lesssim E^g_{N-1} + \varepsilon e^{-2\lambda T}\big (\Vert g-\gamma \Vert _{H^N}^2 + \Vert \Sigma \Vert _{H^{N-1}}^2\big ) + \Lambda (T)^2.\end{aligned}\end{aligned}$$

### Proof

Rearranging (2.8) as $v = w^{-1}(\partial _T h+X^m\hat{\nabla }_m h-F_h)$ and substituting this into (2.8) gives

$$\begin{aligned}\begin{aligned} -w^{-1}&(\partial _T w )v + w^{-1} (\partial _T^2 h +\partial _T(X^m\hat{\nabla }_m h)-\partial _T F_h)\\&= -2v - 9 w\mathcal {L}_{g, \gamma }h - X^m \hat{\nabla }_m v +6F_v.\end{aligned}\end{aligned}$$

Using again (2.8) we note that

$$\begin{aligned} \partial _T (X^a \hat{\nabla }_a h)&= -X^a X^b\hat{\nabla }_a\hat{\nabla }_b h -X^a\hat{\nabla }_a X^b \cdot \hat{\nabla }_b h+\partial _T X^a\cdot \hat{\nabla }_a h\\&\quad +X^m\hat{\nabla }_m (wv+F_h). \end{aligned}$$

Rearranging terms (recall $9w^2 = N^2$) we find

$$\begin{aligned} \mathcal {W}(h) = F_1&:=X^a\hat{\nabla }_a X^b \cdot \hat{\nabla }_b h-\partial _T X^m\cdot \hat{\nabla }_m h -X^m\hat{\nabla }_m (wv+F_h)\\&\qquad +\partial _T F_h +v \partial _T w-2wv - wX^m\hat{\nabla }_m v +6wF_v. \end{aligned}$$

Using Lemma 5.5 and Lemma 5.22 we see that

$$\begin{aligned}\begin{aligned} \Vert F_1 \Vert _{H^{N-2}}&\lesssim \Vert \Sigma \Vert _{H^{N-2}} + \Vert \partial _T F_h\Vert _{H^{N-2}} + \Vert F_v \Vert _{H^{N-2}} + \Lambda (T) \\&\lesssim E^g_{N-1} + \Vert g-\gamma \Vert _{H^N}^2 + \Vert \Sigma \Vert _{H^{N-1}}^2 + \Lambda (T).\end{aligned}\end{aligned}$$

Although the top-order terms involving $g-\gamma$ and $\Sigma$ here look worrying, when squaring the estimate we can then apply the bootstraps to gain the crucial factor of $\varepsilon$.

To derive the equation for *v* we take the $\partial _T$ derivative of (2.8):

$$\begin{aligned} \partial _T^2 v&= -2 \partial _T v - 9\partial _T w \cdot \mathcal {L}_{g, \gamma }h - 9w \mathcal {L}_{g, \gamma }(\partial _T h) - 9w[\partial _T, \mathcal {L}_{g, \gamma }]h - \partial _T X^m \cdot \hat{\nabla }_m v\\&\quad - X^m \hat{\nabla }_m (\partial _T v) + 6 \partial _T F_v. \end{aligned}$$

Substituting in the equation of motion (2.8) where needed, and expanding as $\mathcal {L}_{g, \gamma }(wv) = w\mathcal {L}_{g, \gamma }v-v\hat{\Delta }_{g, \gamma }w-2g^{ab}\hat{\nabla }_a w \hat{\nabla }_b v$, we obtain

$$\begin{aligned} \mathcal {W}(v)&= F_2 :=4v+18 w \mathcal {L}_{g, \gamma }h + 2X^m \hat{\nabla }_m v-12 F_v-9\partial _T w \cdot \mathcal {L}_{g, \gamma }h\\&\quad +9w v\hat{\Delta }_{g, \gamma }w + 18 w g^{ab} \hat{\nabla }_a w \hat{\nabla }_b v +9w\mathcal {L}_{g, \gamma }(X^m\hat{\nabla }_m h)\\&\quad - 9w\mathcal {L}_{g, \gamma }F_h -9w[\partial _T, \mathcal {L}_{g, \gamma }] h +6\partial _T F_v - \partial _T X^m \cdot \hat{\nabla }_m v+2X^m \hat{\nabla }_m v \\  &\quad +9X^m\hat{\nabla }_m(w\mathcal {L}_{g, \gamma }h) + X^a \hat{\nabla }_a X^m\cdot \hat{\nabla }_m v - 6 X^m \hat{\nabla }_m F_v.\end{aligned}$$

Using the $F_h, F_v$ estimates in Lemma 5.5 and Lemma 5.22, together with the commutator estimate in Lemma 5.14, we find

$$\begin{aligned}\begin{aligned} \Vert F_2 \Vert _{H^{N-3}}&\lesssim \Vert \Sigma \Vert _{H^{N-3}} + \Vert g-\gamma \Vert _{H^{N-1}} + \Vert [\partial _T, \mathcal {L}_{g, \gamma }] h\Vert _{H^{N-3}} + \Vert F_v \Vert _{H^{N-2}} \\  &\quad + \Vert \partial _T F_v \Vert _{H^{N-3}} + \Vert F_h \Vert _{H^{N-1}} + \Lambda (T) \\&\lesssim (E^g_{N-1})^{1/2} +\varepsilon e^{-\lambda T} \Vert g-\gamma \Vert _{H^{N-1}} + \Vert g-\gamma \Vert _{H^{N}}^2\\&\quad + \Vert \Sigma \Vert _{H^{N-1}}^2 + E^g_{N-1} + \Lambda (T).\end{aligned}\end{aligned}$$

Note that in the above we also used (5.5) to estimate a term of the form

$$\begin{aligned} \Vert \hat{\nabla }h\Vert _{H^{N-1}} \lesssim \Vert h \Vert _{H^{N}} + \Vert \Upsilon \Vert _{H^{N-1}}\Vert h \Vert _{H^{N-1}} \lesssim \Vert g-\gamma \Vert _{H^{N}} . \end{aligned}$$

$$\square $$

### Proposition 6.3

(Elliptic estimate using $\mathcal {W}, \partial _{\textbf{u}}$ operators) Let *V* be an arbitrary (0, 2)-tensor and $0\le k\le N-2$. Then,

$$\begin{aligned}\begin{aligned} \Vert V\Vert _{H^{k+2}}&\lesssim \Vert \mathcal {W}(V) \Vert _{H^{k}}+ \Vert \partial _{\textbf{u}}\partial _T (V) \Vert _{H^{k}} + |\tau | \Vert u^a \partial _{\textbf{u}}\hat{\nabla }_a(V) \Vert _{H^{k}}.\end{aligned}\end{aligned}$$

### Proof

From (3.9) we have

$$\begin{aligned} \partial _TV = (u^0)^{-1}\Big (\partial _{\textbf{u}}(V) + \tau u^a \hat{\nabla }_a V \Big ), \end{aligned}$$

and thus

$$\begin{aligned}&\partial _T\partial _TV = (u^0)^{-1}\Big (\partial _{\textbf{u}}( \partial _T V) + \tau u^a \hat{\nabla }_a \partial _T V\Big ), \\&\partial _T \hat{\nabla }_b V = (u^0)^{-1}\Big (\partial _{\textbf{u}}(\hat{\nabla }_b V) + \tau u^a \hat{\nabla }_a \hat{\nabla }_b V \Big ). \end{aligned}$$

Recalling $\hat{u}^0= u^0 - 1/3$, we find

6.2 $$\begin{aligned} \begin{aligned} \mathcal {W}(V)&= \partial _T\partial _T V - X^aX^b\hat{\nabla }_a \hat{\nabla }_b V + N^2 \mathcal {L}_{g, \gamma }V \\&= (u^0)^{-1}\Big (\partial _{\textbf{u}}(\partial _T V) + \tau u^a \partial _T \hat{\nabla }_a V\Big ) - X^aX^b\hat{\nabla }_a \hat{\nabla }_b V + N^2 \mathcal {L}_{g, \gamma }V \\&= (u^0)^{-1}\partial _{\textbf{u}}(\partial _T V) + \frac{\tau u^a}{u^0} \frac{1}{u^0}\left( \partial _{\textbf{u}}(\hat{\nabla }_a V) + \tau u^b \hat{\nabla }_b \hat{\nabla }_a V \right) \\&\quad - X^aX^b\hat{\nabla }_a \hat{\nabla }_b V + N^2 \mathcal {L}_{g, \gamma }V \\&= (u^0)^{-1}\partial _{\textbf{u}}(\partial _T V) + \tau u^a (u^0)^{-2}\partial _{\textbf{u}}(\hat{\nabla }_a V) + \mathcal {H}(V).\end{aligned}\end{aligned}$$

We view $N^{-2}\mathcal {H}$ as a perturbation off the elliptic operator $\mathcal {L}_{g, \gamma }$. For $0\le k\le N-2$,

$$\begin{aligned}\begin{aligned} \Vert (N^{-2}\mathcal {H} - \mathcal {L}_{g, \gamma })V\Vert _{H^k}&\lesssim \Vert X^aX^b\hat{\nabla }_a \hat{\nabla }_b V\Vert _{H^k} + \tau ^2 \big \Vert \frac{u^a u^b}{(u^0)^2} \hat{\nabla }_b \hat{\nabla }_a V\big \Vert _{H^k} \\  &\lesssim \varepsilon ^2 e^{(-2+2\mu )T}\big ( \Vert V\Vert _{H^{k+2}}+\Vert \Upsilon \Vert _{H^{k+1}}\Vert V\Vert _{H^{k+1}}\big ) \\  &\lesssim \varepsilon ^2 e^{(-2+2\mu )T}\Vert \mathcal {L}_{g, \gamma }V\Vert _{H^{k}}.\end{aligned}\end{aligned}$$

Suppose now $\mathcal {H}(V) =0$. By definition of $\mathcal {H}$, and elliptic regularity of $\mathcal {L}_{g, \gamma }$, this implies

$$\begin{aligned}\begin{aligned} \Vert N^2 \mathcal {L}_{g, \gamma }(V) \Vert _{L^2}&= \big \Vert X^aX^b\hat{\nabla }_a \hat{\nabla }_b V+\tau ^2 (u^0)^{-2}u^a u^b \hat{\nabla }_b \hat{\nabla }_a V\big \Vert _{L^2} \\  &\lesssim \varepsilon ^2 e^{2(-1+\mu )T} \Vert V\Vert _{H^2} \\  &\le C (C_1)^{-1} \varepsilon ^2 e^{2(-1+\mu )T} \Vert \mathcal {L}_{g, \gamma }V\Vert _{L^2},\end{aligned}\end{aligned}$$

for $C>0$ some constant and $C_1>0$ as in Lemma 3.7. We also have a lower bound

$$\begin{aligned} C' \Vert \mathcal {L}_{g, \gamma }(V) \Vert _{L^2} \le \Vert N^2 \mathcal {L}_{g, \gamma }(V) \Vert _{L^2}. \end{aligned}$$

for another constant $C'>0$. Choosing $\varepsilon$ sufficiently small so that $C (C_1)^{-1} \varepsilon ^2 e^{2(-1+\mu )T} < \varepsilon$, we see these two inequalities imply

$$\begin{aligned} C' \Vert \mathcal {L}_{g, \gamma }(V) \Vert _{L^2} < \varepsilon \Vert \mathcal {L}_{g, \gamma }(V) \Vert _{L^2} . \end{aligned}$$

For $\varepsilon$ sufficiently small this implies $\Vert \mathcal {L}_{g, \gamma }(V)\Vert _{L^2} = 0$ and so $V \in \ker \mathcal {L}_{g, \gamma }$. However, $\ker {\mathcal {L}_{g, \gamma }}=0$, and so for small data $\mathcal {H}$ also has trivial kernel and thus we obtain

$$\begin{aligned} \Vert V\Vert _{H^{k+2}} \lesssim \Vert N^{-2} \mathcal {H}(V)\Vert _{H^k} \lesssim \Vert V\Vert _{H^{k+2}} . \end{aligned}$$

Putting this together with (6.2) we find

$$\begin{aligned}\begin{aligned} \Vert V\Vert _{H^{k+2}}&\lesssim \Vert N^{-2} \mathcal {W}(V) \Vert _{H^{k}}+ \Vert (u^0)^{-1}N^{-2} \partial _{\textbf{u}}\partial _T (V) \Vert _{H^{k}}\\&\quad + |\tau | \Vert (u^0)^{-2}u^a N^{-2} \partial _{\textbf{u}}\hat{\nabla }_a (V) \Vert _{H^{k}}.\end{aligned}\end{aligned}$$

$$\square $$

We can now bring together the previous results and estimate the top-order Sobolev norms of $g, \Sigma$ in terms of our geometric energy functionals ${E^g_{\partial _{\textbf{u}},N-1}}$ and $E^g_{N-1}$.

### Corollary 6.4

We have,

$$\begin{aligned}\begin{aligned} \Vert g-\gamma \Vert _{H^N}^2 +\Vert \Sigma \Vert _{H^{N-1}}^2&\lesssim {E^g_{\partial _{\textbf{u}},N-1}}+ E^g_{N-1} + \Lambda (T)^2.\end{aligned} \end{aligned}$$

### Proof

From Proposition 6.3,

$$\begin{aligned}\begin{aligned} \Vert g-\gamma \Vert _{H^N}^2&\lesssim \Vert \mathcal {W}(h) \Vert _{H^{N-2}}^2+ \Vert \partial _{\textbf{u}}\partial _T (h) \Vert _{H^{N-2}}^2 + |\tau |^2 \Vert u^a \partial _{\textbf{u}}\hat{\nabla }_a (h) \Vert _{H^{N-2}}^2.\end{aligned} \end{aligned}$$

The first term here is treated using Lemma 6.2. For the second term, we begin by using the commutator estimates in Lemma 5.13, and the $\partial _T h$ and $\partial _{\textbf{u}}h$ estimates in Lemma 5.6 and Lemma 5.20 respectively, to find

6.3 $$\begin{aligned} \begin{aligned} \Vert \partial _{\textbf{u}}\hat{\nabla }(h) \Vert _{H^{N-2}}^2&\lesssim \Vert \partial _{\textbf{u}}h\Vert _{H^{N-1}}^2 + \varepsilon ^2 e^{(-2+2\mu ) T} \Vert h\Vert _{H^{N-1}}^2 +\Lambda (T)^2\Vert \partial _T h \Vert _{H^{N-2}} ^2 \\&\lesssim {E^g_{\partial _{\textbf{u}},N-1}}+\varepsilon ^2 e^{2\max \{-1+\mu ,-\lambda \} T}E^g_{N-1} + \Lambda (T)^3,\end{aligned} \end{aligned}$$

Next, using the expression for $\partial _T h$ given in (2.8), together with Lemma 5.4, Corollary 6.5, Lemma 5.20 and Corollary 5.23, we find

$$\begin{aligned}\begin{aligned}&\Vert \partial _{\textbf{u}}\partial _T (h) \Vert _{H^{N-2}}^2 \\&\quad \lesssim \Vert \partial _{\textbf{u}}\Sigma \Vert _{H^{N-2}}^2 + \Vert \Sigma \Vert _{H^{N-2}}^2 \Vert \partial _{\textbf{u}}N \Vert _{H^{N-2}}^2 + \Vert \partial _{\textbf{u}}X \Vert _{H^{N-2}}^2\Vert \hat{\nabla }h \Vert _{H^{N-2}}^2 \\&\quad \quad + \Vert X\Vert _{H^{N-2}}^2\Vert \partial _{\textbf{u}}\hat{\nabla }h \Vert _{H^{N-2}}^2 + \Vert \partial _{\textbf{u}}F_h \Vert _{H^{N-2}}^2. \\&\quad \lesssim {E^g_{\partial _{\textbf{u}},N-1}}+ E^g_{N-1} + \varepsilon e^{\max \{-1+\mu ,-\lambda \} T}\big (\Vert \Sigma \Vert _{H^{N-1}}^2 + \Vert g-\gamma \Vert _{H^N}^2\big ) + \Lambda (T)^2.\end{aligned}\end{aligned}$$

Using again (6.3) we find

$$\begin{aligned}\begin{aligned}&|\tau |^2 \Vert u^a \partial _{\textbf{u}}\hat{\nabla }(h) \Vert _{H^{N-2}}^2\\&\quad \lesssim |\tau |^2 \Vert u \Vert _{H^{N-2}}^2 \big ({E^g_{\partial _{\textbf{u}},N-1}}+\varepsilon ^2 e^{\max \{-2+2\mu ,-2\lambda \} T}E^g_{N-1} + \Lambda (T)^3\big ) \\  &\quad \lesssim {E^g_{\partial _{\textbf{u}},N-1}}+ E^g_{N-1} + \Lambda (T)^4.\end{aligned}\end{aligned}$$

Putting this all together,

6.4 $$\begin{aligned} \begin{aligned} \Vert g-\gamma \Vert _{H^N}^2&\lesssim {E^g_{\partial _{\textbf{u}},N-1}}+ E^g_{N-1} + \varepsilon \big (\Vert \Sigma \Vert _{H^{N-1}}^2 + \Vert g-\gamma \Vert _{H^N}^2\big ) + \Lambda (T)^2.\end{aligned}\nonumber \\ \end{aligned}$$

We follow the same steps for the $\Sigma$ estimate. From Proposition 6.3,

$$\begin{aligned}\begin{aligned} \Vert \Sigma \Vert _{H^{N-1}}^2&\lesssim \Vert \mathcal {W}(v) \Vert _{H^{N-3}}^2+ \Vert \partial _{\textbf{u}}\partial _T (v) \Vert _{H^{N-3}}^2 + |\tau |^2 \Vert u^a \partial _{\textbf{u}}\hat{\nabla }_a (\Sigma ) \Vert _{H^{N-3}}^2.\end{aligned} \end{aligned}$$

The first term here is treated using Lemma 6.2. For the second term, we begin by using Lemma 5.6, Lemma 5.20 and the commutator estimates in Lemma 5.13 to show that

6.5 $$\begin{aligned} \begin{aligned} \Vert \partial _{\textbf{u}}\hat{\nabla }(\Sigma ) \Vert _{H^{N-3}}^2&\lesssim \Vert \partial _{\textbf{u}}\Sigma \Vert _{H^{N-2}}^2 + \varepsilon ^2 e^{(-2+2\mu ) T} \Vert \Sigma \Vert _{H^{N-2}}^2 +\Lambda (T)^2 \Vert \partial _T \Sigma \Vert _{H^{N-3}}^2 \\&\lesssim {E^g_{\partial _{\textbf{u}},N-1}}+ E^g_{N-1} + \varepsilon e^{\max \{-1+\mu ,-\lambda \} T}\\&\quad \big (\Vert \Sigma \Vert _{H^{N-1}}^2 + \Vert g-\gamma \Vert _{H^N}^2\big ) + \Lambda (T)^2.\end{aligned} \end{aligned}$$

Next, by Lemma 5.6, Lemma 5.20 and the commutator estimate of Lemma 5.14, we note

$$\begin{aligned}\begin{aligned} \Vert \partial _{{\textbf {u}}}\mathcal {L}_{g, \gamma }h\Vert _{H^{N-3}}^2&\lesssim \Vert \partial _{{\textbf {u}}}h\Vert _{H^{N-1}}^2 + \Vert [ \partial _{{\textbf {u}}},\mathcal {L}_{g, \gamma }] h\Vert _{H^{N-3}}^2 \\  &\lesssim {E^g_{\partial _{{\textbf {u}}},N-1}}+\varepsilon ^2 e^{\max \{-2+2\mu ,-2\lambda \} T}E^g_{N-1} \\  &\quad +\Lambda (T)^2 \big (\Vert \Sigma \Vert _{H^{N-1}}^2 + \Vert g-\gamma \Vert _{H^N}^2\big )\\  &\quad + \Lambda (T)^4. \end{aligned}\end{aligned}$$

We can now use the expression for $\partial _T v$ given in (2.8) and bring together these previous estimates:

$$\begin{aligned}\begin{aligned} \Vert \partial _{\textbf{u}}\partial _T (v) \Vert _{H^{N-3}}^2&\lesssim \Vert \partial _{\textbf{u}}\Sigma \Vert _{H^{N-3}}^2 + \Vert \mathcal {L}_{g, \gamma }h \Vert _{H^{N-3}}^2 \Vert \partial _{\textbf{u}}N \Vert _{H^{N-3}}^2 + \Vert \partial _{\textbf{u}}\mathcal {L}_{g, \gamma }h\Vert _{H^{N-3}}^2 \\  &\quad + \Vert \partial _{\textbf{u}}X \Vert _{H^{N-3}}^2\Vert \hat{\nabla }\Sigma \Vert _{H^{N-3}}^2 + \Vert X\Vert _{H^{N-3}}^2\Vert \partial _{\textbf{u}}\hat{\nabla }\Sigma \Vert _{H^{N-3}}^2 + \Vert \partial _{\textbf{u}}F_v \Vert _{H^{N-3}}^2 \\&\lesssim \Vert \partial _{\textbf{u}}\Sigma \Vert _{H^{N-3}}^2 + \Vert \partial _{\textbf{u}}\mathcal {L}_{g, \gamma }h\Vert _{H^{N-3}}^2 + \Vert \partial _{\textbf{u}}F_v \Vert _{H^{N-3}}^2 + \Lambda (T)^2 \\&\lesssim {E^g_{\partial _{\textbf{u}},N-1}}+\varepsilon e^{\max \{-1+\mu ,-\lambda \} T}\big (\Vert \Sigma \Vert _{H^{N-1}}^2 + \Vert g-\gamma \Vert _{H^N}^2\big ) + \Lambda (T)^2.\end{aligned} \end{aligned}$$

In the above we used Lemma 5.4, Corollary 6.5, Lemma 5.23 and Lemma 5.20. Using again (6.5) and Lemma 5.20, we find

$$\begin{aligned} \begin{aligned} |\tau |^2 \Vert u^a \partial _{{\textbf {u}}}\hat{\nabla }(\Sigma ) \Vert _{H^{N-3}}^2&\lesssim {E^g_{\partial _{{\textbf {u}}},N-1}}+ E^g_{N-1} + \varepsilon e^{\max \{-1+\mu ,-\lambda \} T}\Lambda (T)\times \\  &\quad \big (\Vert \Sigma \Vert _{H^{N-1}}^2 + \Vert g-\gamma \Vert _{H^N}^2\big ) +\Lambda (T)^3. \end{aligned} \end{aligned}$$

Finally, putting this all together gives

6.6 $$\begin{aligned} \begin{aligned} \Vert \Sigma \Vert _{H^{N-1}}^2&\lesssim {E^g_{\partial _{\textbf{u}},N-1}}+ E^g_{N-1} + \varepsilon \big (\Vert \Sigma \Vert _{H^{N-1}}^2 + \Vert g-\gamma \Vert _{H^N}^2\big ) + \Lambda (T)^2.\end{aligned} \end{aligned}$$

The conclusion then follows by adding the estimates (6.4) and (6.6) together and taking $\varepsilon$ sufficiently small so that we can absorb the $\varepsilon (\Vert \Sigma \Vert _{H^{N-1}}^2 + \Vert g-\gamma \Vert _{H^N}^2)$ term onto the left hand side. $\square$

We now provide new estimates on dust derivatives acting on our variables $N, X, g, \Sigma$ and, for the latter two variables, apply Corollary 6.4.

### Corollary 6.5

For *I* a multi-index,

$$\begin{aligned}\begin{aligned}&\sum _{|I|\le N-1}\Vert \partial _{\textbf{u}}\nabla ^I X\Vert _{L^2}+ \Vert \partial _{\textbf{u}}\nabla ^I \widehat{N}\Vert _{L^2} \lesssim \Lambda (T), \\&\sum _{|I|\le N-1}\Vert \partial _{\textbf{u}}\nabla ^I (g-\gamma )\Vert _{L^2} + \sum _{|I|\le N-2}\Vert \partial _{\textbf{u}}\nabla ^I \Sigma \Vert _{L^2}\\&\quad \lesssim ({E^g_{\partial _{\textbf{u}},N-1}})^{1/2} + (E^g_{N-1})^{1/2}+ \Lambda (T).\end{aligned}\end{aligned}$$

The same estimates hold with $\nabla$ replaced by $\hat{\nabla }$.

### Proof

By Lemma 5.20 and Corollary 6.4

$$\begin{aligned}\begin{aligned} \Vert \partial _{\textbf{u}}h \Vert _{H^{N-1}} + \Vert \partial _{\textbf{u}}\Sigma \Vert _{H^{N-2}}&\lesssim ({E^g_{\partial _{\textbf{u}},N-1}})^{1/2} + (E^g_{N-1})^{1/2} + \Lambda (T).\end{aligned} \end{aligned}$$

Next, let $\nabla ^{I} = \nabla _{a_1} \ldots \nabla _{a_{k}}$ where $k:=|I|\le N-1$ and let *V* be a (0, 2)-tensor on *M*. Then, by (5.1), (5.2) and Lemma 5.13,

$$\begin{aligned}\begin{aligned} \Vert [ \partial _{\textbf{u}}, \nabla ^I] V \Vert _{L^2}&\lesssim \Vert [ \partial _{\textbf{u}}, \nabla _{a_1}] \nabla _{a_2} \ldots \nabla _{a_k} V \Vert _{L^2} + \cdots + \Vert [ \partial _{\textbf{u}}, \nabla ] V \Vert _{H^{k-1}} \\  &\lesssim \varepsilon e^{\max \{-1+\mu ,-\lambda \}T} \Vert V\Vert _{H^{k}} + \Lambda (T)\Vert \partial _T V\Vert _{H^{k-1}}\\&\quad + \Lambda (T) \Vert \partial _T \Gamma (g) \Vert _{H^{N-3}} \Vert V \Vert _{H^{k-2}}. \end{aligned}\end{aligned}$$

This implies

$$\begin{aligned}\begin{aligned} \Vert \partial _{\textbf{u}}\nabla ^I V \Vert _{L^2} \lesssim \Vert \partial _{\textbf{u}}V \Vert _{H^k} + \varepsilon e^{\max \{-1+\mu ,-\lambda \} T}\Vert V\Vert _{H^{k}} + \Lambda (T)\Vert \partial _T V\Vert _{H^{k-1}} .\end{aligned}\end{aligned}$$

A simple check shows that when considering the appropriate commutator expression (see (5.10)) on the scalar lapse we will pick up $\Vert \widehat{N}\Vert _{H^k}$ terms and not $\Vert N \Vert _{H^k}$. The result then follow, using Lemma 5.4 and Lemma 5.6. $\square$

### Lemma 6.6

Let *V* be a (0, 2)-tensor on *M* and let $p\in \{1,2\}$. Then

$$\begin{aligned}\begin{aligned} \Vert \partial _{\textbf{u}}V\Vert _{H^p}&\lesssim \sum _{|I|\le p} \Vert \partial _{\textbf{u}}\nabla ^I V \Vert _{L^2} + \varepsilon e^{\max \{-1+\mu ,-\lambda \} T}\Vert V\Vert _{H^{1}} +\Lambda (T)\Vert \partial _T V\Vert _{H^{p-1}}.\end{aligned} \end{aligned}$$

### Proof

A straight forward application of commutator identities. $\square$

## Lapse and Shift Estimates

In this section we first establish the basic estimates on the lapse, shift and their time derivatives using elliptic estimates and general formula presented in [1]. The most exciting results lie in the novel top-order lapse and shift estimates given in Sect. 7.1.

### Lemma 7.1

For $$3\le k \le N$$,

$$\begin{aligned} \begin{aligned} \Vert \widehat{N}\Vert _{H^k}&\lesssim |\tau | \Vert \rho \Vert _{H^{k-2}} + \varepsilon ^2 e^{-2\lambda T},\\ \Vert X \Vert _{H^k}&\lesssim |\tau | \Vert \rho \Vert _{H^{k-3}} + \varepsilon ^2 e^{\max \{-2\lambda , -2+\mu \}T}.\end{aligned} \end{aligned}$$

### Proof

Recall from (2.7b) the lapse equation of motion

$$\begin{aligned} (\Delta - \tfrac{1}{3})N = N(|\Sigma |_g^2 + \tau \eta ) - 1. \end{aligned}$$

By elliptic regularity for the standard Laplacian $\Delta$ this implies

$$\begin{aligned}\begin{aligned} \Vert \widehat{N}\Vert _{H^k}&\le C \big ( \Vert \Sigma \Vert ^2_{H^{k-2}} + |\tau | \Vert \eta \Vert _{H^{k-2}} \big ),\end{aligned} \end{aligned}$$

and the desired result follows by Lemma 4.4.

Similarly, from the shift equation of motion (2.7b), we find that

$$\begin{aligned}\begin{aligned} \Vert X \Vert _{H^k}&\le C \big ( \Vert \Sigma \Vert _{H^{k-2}}^2 + \Vert g-\gamma \Vert _{H^{k-1}}^2 + |\tau | \Vert \eta \Vert _{H^{k-3}} + \tau ^2 \Vert N \jmath \Vert _{H^{k-2}} \big ),\end{aligned}\end{aligned}$$

and the desired result follows by Lemma 4.4. $\square$

### Lemma 7.2

For $$4\le k \le N-1$$,

$$\begin{aligned}\begin{aligned} \Vert \partial _T N \Vert _{H^k} + \Vert \partial _T X \Vert _{H^k}&\lesssim \Vert \widehat{N}\Vert _{H^k}+\Vert X\Vert _{H^k} + |\tau |\Vert \rho \Vert _{H^{k-2}} + \varepsilon ^2 e^{\max \{-2\lambda , -2+\mu \}T}.\end{aligned}\end{aligned}$$

### Proof

Let $4\le k \le N-1$. Following [1], we have

$$\begin{aligned}\begin{aligned} \Vert \partial _T N \Vert _{H^k}&\lesssim \Vert \widehat{N}\Vert _{H^k}+\Vert X\Vert _{H^k} + \Vert \Sigma \Vert _{H^{k-1}}^2 + \Vert g-\gamma \Vert _{H^k}^2\\&\quad + |\tau | \big (\Vert S \Vert _{H^{k-2}} + \Vert \eta \Vert _{H^{k-2}}+\Vert \partial _T \eta \Vert _{H^{k-2}}\big ) \\  &\lesssim \Vert \widehat{N}\Vert _{H^k}+\Vert X\Vert _{H^k} + |\tau |\Vert \rho \Vert _{H^{k-2}}+ \varepsilon ^2 e^{\max \{-2\lambda , -2+\mu \}T},\end{aligned}\end{aligned}$$

where we used Lemma 4.4 and Lemma 5.12.

Again, using a general expression given in [1, §7], we have

$$\begin{aligned}\begin{aligned} \Vert \partial _T X \Vert _{H^k}&\lesssim \Vert \widehat{N}\Vert _{H^k}+ \Vert X\Vert _{H^{k}}+ \Vert \Sigma \Vert _{H^{k}}^2 +\Vert g-\gamma \Vert _{H^k} ^2 \\&\quad + \tau \big (\Vert S \Vert _{H^{k-2}} + \Vert \eta \Vert _{H^{k-2}}+\Vert \partial _T \eta \Vert _{H^{k-2}}\big )+ \tau ^2 \Vert \jmath \Vert _{H^{k-1}} + \tau ^3 \Vert \underline{T} \Vert _{H^{k-1}} \\&\lesssim \Vert \widehat{N}\Vert _{H^k}+ \Vert X\Vert _{H^{k}} + |\tau | \Vert \rho \Vert _{H^{k-2}} +\varepsilon ^2 e^{\max \{-2\lambda , -2+\mu \}T},\end{aligned}\end{aligned}$$

where we again used Lemma 4.4 and Lemma 5.12. $\square$

### Additional Top-order Lapse and Shift Estimates

In this section we establish important auxiliary estimates in two propositions for the lapse and shift variables. The first proposition is used to estimate $\partial _{\textbf{u}}\nabla _i \nabla _j N$. This somewhat unusual expression comes from a dust derivative acting on the first term in $F_v$ (see Definition 2.8), and appears later on in the energy estimates for $E^g_{tot}$.

#### Proposition 7.3

(Top-order auxiliary lapse estimate) We have

$$\begin{aligned} \big (\Delta -\tfrac{1}{3}\big )\partial _{\textbf{u}}\nabla _i \nabla _j N =\mathcal {F}_{N,\textbf{u}} \end{aligned}$$

where,

$$\begin{aligned} \Vert \mathcal {F}_{N,\textbf{u}}\Vert _{H^{N-4}} \lesssim \Lambda (T) + {E^g_{\partial _{\textbf{u}},N-1}}+ E^g_{N-1}. \end{aligned}$$

#### Proof

Start by commuting the lapse Eq. (2.7b) with $\partial _{\textbf{u}}\nabla _i \nabla _j$:

$$\begin{aligned} \begin{aligned} \big (\Delta -\tfrac{1}{3}\big )\partial _{\textbf{u}}\nabla _i \nabla _j N&= [\Delta ,\partial _{\textbf{u}}]\nabla _i\nabla _j N + \partial _{\textbf{u}}[\Delta , \nabla _i\nabla _j]N + \partial _{\textbf{u}}\nabla _i \nabla _j \Big ( N(|\Sigma |_g^2 - \tau \eta )\Big )\\&=: L_1 + L_2 +L_3=: \mathcal {F}_{N,\textbf{u}}.\end{aligned} \end{aligned}$$

We investigate each of the terms in $$\mathcal {F}_{N,\textbf{u}}$$ separately. The first term $$L_1 $$ we estimate using (5.2), (5.3) and Lemma 5.18

$$\begin{aligned}\begin{aligned} \Vert L_1\Vert _{H^{N-4}}&\lesssim \Lambda (T)\Vert \partial _T \nabla _i\nabla _j N\Vert _{H^{N-3}} + \varepsilon e^{\max \{-1+\mu ,-\lambda \} T}\Vert \nabla _i\nabla _j N\Vert _{H^{N-2}} \\  &\lesssim \Lambda (T)\Big ( \Vert \partial _T N\Vert _{H^{N-1}} + \Vert [\partial _T, \nabla _i]\nabla _j N\Vert _{H^{N-3}} \Big ) \\  &\quad + \varepsilon e^{\max \{-1+\mu ,-\lambda \} T}\Vert \widehat{N}\Vert _{H^{N}} \\  &\lesssim \Lambda (T)^2 + \varepsilon e^{\max \{-1+\mu ,-\lambda \} T}\Lambda (T).\end{aligned}\end{aligned}$$

For the next term, $L_2$, we first compute, for $\phi$ a scalar,

$$\begin{aligned}\begin{aligned} [\Delta , \nabla _i] \phi&= 2g^{ab}\nabla _{[a}\nabla _{i]}\nabla _b \phi = -\text {Ric}^c _i \nabla _c \phi , \\ [\Delta ,\nabla _i]\nabla _j\phi&= \text {Ric}^c _i\nabla _c \nabla _j\phi - (\nabla ^a \text {Riem}^c _{jai})\nabla _c\phi - 2\text {Riem}^c _{jai}\nabla ^a\nabla _c\phi .\end{aligned} \end{aligned}$$

Thus

$$\begin{aligned}\begin{aligned} L_2&=\partial _{\textbf{u}}([\Delta ,\nabla _i]\nabla _j N) + \partial _{\textbf{u}}\nabla _i([\Delta ,\nabla _j N) \\  &= -\Big ( (\partial _{\textbf{u}}\nabla ^a \text {Riem}^c _{jai})+(\partial _{\textbf{u}}\nabla _i \text {Ric}^c _j) \Big )\nabla _c N - \Big (\nabla ^a \text {Riem}^c _{jai}+\nabla _i \text {Ric}^c _j \Big ) \partial _{\textbf{u}}\nabla _c N \\  &\quad +\Big ( (\partial _{\textbf{u}}\text {Ric}^c _i)\nabla _c\nabla _j N -(\partial _{\textbf{u}}\text {Ric}^c _{j} )\nabla _i\nabla _c N - 2 (\partial _{\textbf{u}}\text {Riem}^c _{jai})\nabla ^a\nabla _c N \Big ) \\  &\quad +\Big ( \text {Ric}^c _i(\partial _{\textbf{u}}\nabla _c\nabla _j N ) - \text {Ric}^c _{j} (\partial _{\textbf{u}}\nabla _i\nabla _c N ) - 2\text {Riem}^c _{jai}(\partial _{\textbf{u}}\nabla ^a\nabla _c N) \Big ) \\&=: -(L_{21})-(L_{22})+(L_{23})+(L_{24}) \end{aligned}. \end{aligned}$$

The second and last terms here are easy to estimate using Lemma 5.4 and Lemma 5.13 (note also that the expressions (5.1) and (5.10) simplify when calculated for a scalar)

$$\begin{aligned} \begin{aligned} \Vert&|L_{22}|+| L_{24}|\Vert _{H^{N-4}} \\&\lesssim \Vert \text {Riem}\Vert _{H^{N-3}}\Big ( \Vert \partial _{\textbf{u}}N\Vert _{H^{N-4}} + \Vert [\partial _{\textbf{u}}, \nabla ]N\Vert _{H^{N-3}} + \Vert [\partial _{\textbf{u}}, \nabla ]\nabla N\Vert _{H^{N-4}}\Big ) \\&\lesssim \Vert \text {Riem}\Vert _{H^{N-3}} \Big ( \Lambda (T) + \Lambda (T) \Vert \partial _T N\Vert _{H^{N-3}} + \varepsilon e^{\max \{-1+\mu ,-\lambda \} T}\Vert N\Vert _{H^{N-2}} \Big ) \\&\lesssim \Lambda (T).\end{aligned} \end{aligned}$$

For the other two terms, $L_{21}$ and $L_{23}$, we schematically write

$$\begin{aligned}\begin{aligned} \partial _{\textbf{u}}\text {Riem}&= u^0 \partial _T (\partial \Gamma + \Gamma \Gamma ) - \tau u^c \hat{\nabla }_c \text {Riem}\\&= u^0 (\partial \partial _T \Gamma + \Gamma \partial _T \Gamma ) - \tau u^c \nabla _c \text {Riem}+ \tau u^c *\Upsilon *\text {Riem}, \\ \partial _{\textbf{u}}\nabla \text {Riem}&= \nabla \partial _{\textbf{u}}\text {Riem}+ [\partial _{\textbf{u}}, \nabla ]\text {Riem},\end{aligned} \end{aligned}$$

and thus

$$\begin{aligned} \begin{aligned}&\Vert |L_{21}|+ |L_{23}|\Vert _{H^{N-4}} \\  &\quad \lesssim \Vert \widehat{N}\Vert _{H^{N-2}} \Big (\Vert \partial _T \Gamma \Vert _{H^{N-2}} + \Vert \partial _T \Gamma \Vert _{H^{N-3}}\Vert \Gamma \Vert _{H^{N-3}} + \Vert \partial _T \Gamma \Vert _{H^{N-4}}\Vert \text{ Riem }\Vert _{H^{N-4}} \\  &\qquad + \varepsilon e^{\max \{-1+\mu ,-\lambda \} T}\Vert \text{ Riem }\Vert _{H^{N-2}} + \varepsilon \Vert \partial _T \text{ Riem }\Vert _{H^{N-4}} \Big ) \\  &\quad \lesssim \varepsilon e^{-\lambda T} \Vert \widehat{N}\Vert _{H^{N-2}} + \varepsilon e^{\max \{-1+\mu ,-\lambda \} T}\Vert \widehat{N}\Vert _{H^{N-2}} \Vert \text{ Riem }\Vert _{H^{N-2}} \lesssim \Lambda (T).\end{aligned} \end{aligned}$$

Finally we compute

$$\begin{aligned}\begin{aligned} L_3&= \nabla _i\nabla _j\partial _{\textbf{u}}\big ( N(|\Sigma |_g^2 - \tau \eta )\big ) + \nabla _i [\partial _{\textbf{u}},\nabla _j]\big ( N(|\Sigma |_g^2 - \tau \eta )\big )\\&\quad + [\partial _{\textbf{u}},\nabla _i]\nabla _j \big ( N(|\Sigma |_g^2 - \tau \eta )\big )\\&=: L_{31}+L_{32}+L_{33} \end{aligned}\end{aligned}$$

By the matter estimates in Lemma 4.4 and Lemma 5.10, as well as the geometry estimates in Lemma 5.4 and Corollary 6.5, we find

$$\begin{aligned}\begin{aligned} \Vert L_{31}\Vert _{H^{N-4}}&\lesssim \Vert \partial _{\textbf{u}}N\Vert _{H^{N-2}} \big ( \Vert \Sigma \Vert _{H^{N-2}}^2+|\tau | \Vert \eta \Vert _{H^{N-2}}\big ) + \Vert \Sigma \Vert _{H^{N-2}}\Vert \partial _{\textbf{u}}\Sigma \Vert _{H^{N-2}} \\  &\quad + |\tau | \Vert \eta \Vert _{H^{N-2}}+ |\tau | \Vert \partial _{\textbf{u}}\eta \Vert _{H^{N-2}} \\  &\lesssim \Lambda (T) + \Vert \Sigma \Vert _{H^{N-2}}\big ( (E^g_{\partial _{\textbf{u}},N-1})^{1/2} + (E^g_{N-1})^{1/2}\big ).\end{aligned}\end{aligned}$$

Similarly by Lemma 4.4, Lemma 5.12, Corollary 5.7 and the commutator estimate of Lemma 5.13,

$$\begin{aligned}\begin{aligned} \Vert L_{32}\Vert _{H^{N-4}}&\lesssim \varepsilon e^{\max \{-1+\mu ,-\lambda \} T} \big ( \Vert \Sigma \Vert _{H^{N-2}} ^2 + |\tau |\Vert \eta \Vert _{H^{N-2}} \big )\\&\quad +\Lambda (T) \Vert \partial _T N\Vert _{H^{N-3}} \big ( \Vert \Sigma \Vert _{H^{N-3}}^2+ |\tau | \Vert \eta \Vert _{H^{N-3}}\big ) \\  &\quad + \Lambda (T) \Vert \Sigma \Vert _{H^{N-3}}\Vert \partial _T \Sigma \Vert _{H^{N-3}} + \Lambda (T) |\tau | \Vert \eta \Vert _{H^{N-3}}\\&\quad +\Lambda (T) |\tau |\Vert \partial _T \eta \Vert _{H^{N-3}} \\  &\lesssim \varepsilon e^{\max \{-1+\mu ,-\lambda \} T} E^g_{N-1} + \varepsilon e^{\max \{-1+\mu ,-\lambda \} T} \Lambda (T).\end{aligned}\end{aligned}$$

The final estimate follows in the same way:

$$\begin{aligned}\begin{aligned} \Vert L_{33}\Vert _{H^{N-4}}&\lesssim \varepsilon e^{\max \{-1+\mu ,-\lambda \} T} E^g_{N-1} + \varepsilon e^{\max \{-1+\mu ,-\lambda \} T} \Lambda (T).\end{aligned} \end{aligned}$$

Putting this all together we find that

$$\begin{aligned} \Vert \mathcal {F}_{N,\textbf{u}}\Vert _{H^{N-4}} \lesssim \Lambda (T) + \Vert \Sigma \Vert _{H^{N-2}}\big ( (E^g_{\partial _{\textbf{u}},N-1})^{1/2} + (E^g_{N-1})^{1/2}\big ), \end{aligned}$$

and so the conclusion follows by Lemma 4.5. $\square$

#### Remark 7.4

In our energy estimates later on we need to estimate a term of the type $\partial _{\textbf{u}}\hat{\nabla }^{N-2} F_v$ where $\hat{\nabla }^{N-2}$ indicates $N-2$ covariant derivatives and $F_v$ is given in Definition 2.8. We would like to commute the $\partial _{\textbf{u}}$ operator past these covariant derivatives. However, $F_v$ contains a $\nabla \nabla N$ term, and we cannot commute $\partial _{\textbf{u}}$ past $N-2$ copies of $\nabla$ as well as the extra two derivatives in $\nabla \nabla N$ since we only control $u^a$ in $H^{N-1}$. The previous proposition crucially allows us to avoid this issue. Note also that by commuting in the $\partial _{\textbf{u}}$ operator, instead of doing the rough expansion $\partial _{\textbf{u}}\sim \partial _T + \tau u^c *\nabla$, we gain an additional derivative in Corollary 7.5 compared to Corollary 5.23.

#### Corollary 7.5

For *I* a multi-index of order $|I|\le N-2$,

$$\begin{aligned}\begin{aligned} \Vert \partial _{\textbf{u}}\hat{\nabla }^I F_v\Vert _{L^2}&\lesssim \Lambda (T) + {E^g_{\partial _{\textbf{u}},N-1}}+E^g_{N-1}.\end{aligned} \end{aligned}$$

#### Proof

Write $\hat{\nabla }^{I} = \hat{\nabla }_{a_1} \ldots \hat{\nabla }_{a_{k}}$ where $k:=|I|\le N-2$. Using the definition of $F_v$ in Definition 2.8 and the estimates in Corollary 6.5, we see the most subtle terms (from the point of view of regularity) are $\nabla _i\nabla _j N$ and $N\tau S_{ij}$. For these terms we look at what happens when we commute in the $\partial _{\textbf{u}}$ operator using Lemma 5.13:

$$\begin{aligned}\begin{aligned}&\Vert [\partial _{\textbf{u}}, \hat{\nabla }^I] \nabla _i\nabla _j N \Vert _{L^2} \\&\quad \lesssim \Vert [ \partial _{\textbf{u}}, \hat{\nabla }_{a_1}] \hat{\nabla }_{a_2} \ldots \hat{\nabla }_{a_k} \nabla _i\nabla _j N \Vert _{L^2} + \cdots + \Vert [ \partial _{\textbf{u}}, \hat{\nabla }] \nabla _i\nabla _j N \Vert _{H^{k-1}} \\  &\quad \lesssim \varepsilon e^{(-1+\mu ) T} \Vert \widehat{N}\Vert _{H^{k+2}} +\Lambda (T)\Vert \partial _T N \Vert _{H^{k+1}} +\Lambda (T)\Vert [\partial _T ,\nabla _i\nabla _j] N \Vert _{H^{k-1}} .\end{aligned} \end{aligned}$$

Thus, by the commutator estimates (5.1) and (5.2), as well as Proposition 7.3,

$$\begin{aligned}\begin{aligned} \Vert \partial _{\textbf{u}}\hat{\nabla }^I \nabla _i\nabla _j N \Vert _{L^2}&\lesssim \Vert \partial _{\textbf{u}}\nabla _i\nabla _j N \Vert _{H^k} + \Vert [\partial _{\textbf{u}}, \hat{\nabla }^I] \nabla _i\nabla _j N \Vert _{L^2} \\  &\lesssim \Vert \mathcal {F}_{N, \textbf{u}} \Vert _{H^{k-2}} + \Lambda (T) \\  &\lesssim \Lambda (T) + \Vert \Sigma \Vert _{H^{N-2}}\big ( (E^g_{\partial _{\textbf{u}},N-1})^{1/2} + (E^g_{N-1})^{1/2}\big ).\end{aligned}\end{aligned}$$

Similarly, using the matter estimates in Lemma 4.4, Lemma 5.10 and Lemma 5.12, as well as $\partial _{\textbf{u}}N$ estimates in Lemma 5.4, we find

$$\begin{aligned}&\Vert \partial _{\textbf{u}}\hat{\nabla }^I (\tau N S_{ij})\Vert _{L^2_{g,\gamma }} \\&\quad \lesssim |\tau | \big (\Vert S\Vert _{H^{k}} + \Vert \partial _{\textbf{u}}S\Vert _{H^{k}} + \Vert \partial _{\textbf{u}}N \Vert _{H^k} \Vert S\Vert _{H^k} + \varepsilon e^{(-1+\mu ) T} \Vert S\Vert _{H^{k}} \\&\qquad +\Lambda (T)\Vert \partial _TS\Vert _{H^{k-1}} + \Lambda (T)\Vert S\Vert _{H^{k-1}} +\Lambda (T)\Vert \partial _T N\Vert _{H^{k-1}}\Vert S\Vert _{H^{k-1}} \big ) \\&\lesssim \Lambda (T).\end{aligned}$$

$$\square $$

In our energy estimates later on we need to estimate a term of the type $\partial _{\textbf{u}}\hat{\nabla }^{N-1} F_h$. Similar to the issue discussed in Remark 7.4, the problematic term here is the $\mathscr {L}_X g$ term appearing in $F_h$ (see Definition 2.8). The second proposition of this section estimates this problematic term. Since, however, the shift is not scalar-valued like the lapse, a replication of the ideas used in Proposition 7.3 ends up failing. Instead, our proof involves a remarkable combination of commutator estimates, the Bianchi identity and the Einstein equations in the CMCSH gauge.

#### Proposition 7.6

(Top-order estimate for $\mathscr {L}_X g$) We have

$$\begin{aligned}&\Big | \big (\partial _{\textbf{u}}\mathcal {L}_{g, \gamma }^{\ell -1}(\Delta \mathscr {L}_X g),\partial _{\textbf{u}}\mathcal {L}_{g, \gamma }^{\ell }(h)\big )_{L^2_{g,\gamma }} \Big | \\&\quad \lesssim \Lambda (T) {E^g_{\partial _{\textbf{u}},N-1}}+ \Lambda (T) E^g_{N-1} + \Lambda (T) ({E^g_{\partial _{\textbf{u}},N-1}})^{1/2}.\end{aligned}$$

#### Proof

Recall $2(\ell -1) = N-3$. We first define $\mathcal {F}^X_a$ by rewriting the shift Eq. (2.7b) as

$$\begin{aligned} \Delta X_a = - \text {Ric}^c _a X_c + \mathcal {F}^X_a. \end{aligned}$$

By contracting the Bianchi identity, one finds that

$$\begin{aligned} \nabla ^a\text {Riem}_{abcd} = \nabla _c \text {Ric}_{bd} - \nabla _d \text {Ric}_{bc}. \end{aligned}$$

Using this, and the fact that $\nabla$ is a torsion-free connection for the metric *g*, we can show that

$$\begin{aligned}\begin{aligned} \Delta (\mathscr {L}_X g)_{ab}&= \nabla _a \Delta X_b + \nabla _b \Delta X_a + [\Delta ,\nabla _a]X_b + [\Delta ,\nabla _b]X_a \\&= \nabla _a(-\text {Ric}^c _b) X_c+ \nabla _a\mathcal {F}^X_b + \nabla _b (-\text {Ric}^c _a) X_c+ \nabla _b \mathcal {F}^X_a \\  &\quad -g^{ij} \nabla _i(\text {Riem}^k _{bja})X_k -g^{ij} \nabla _i(\text {Riem}^k _{ajb})X_k + 2 E_{(ab)} \\&= -2 X^k \nabla _k \text {Ric}_{ab} + \nabla _a \mathcal {F}^X_b + \nabla _b \mathcal {F}^X_a + 2 E_{(ab)},\end{aligned}\end{aligned}$$

where we have introduced the error terms

$$\begin{aligned}\begin{aligned} E_{ij} :=&- \text {Ric}^c _j\nabla _i X_c - 2 \text {Riem}^k _{jci}\nabla ^c X_k + \text {Ric}_{ki}\nabla ^k X_j .\end{aligned}\end{aligned}$$

As first noted in [2], in the CMCSH gauge, we have

$$\begin{aligned} \text {Ric}_{ab} = -\frac{2}{9} g_{ab} -\frac{1}{2} \mathcal {L}_{g, \gamma }h_{ab} + J_{ab}, \end{aligned}$$

and thus

7.1 $$\begin{aligned} \begin{aligned} \Delta \mathscr {L}_X g_{ab} = X^k \nabla _k \mathcal {L}_{g, \gamma }h_{ab} -2 X^k \nabla _k J_{ab} + 2 \nabla _{(a} \mathcal {F}^X_{b)} + 2 E_{(ab)}.\end{aligned} \end{aligned}$$

We now need to estimate each term in the RHS of (7.1). The first term requires the most care. We begin with an identity, valid for *V* an arbitrary (0, 2)-tensor and $k \in \mathbb {Z}_{\ge 0}$,

7.2 $$\begin{aligned} \begin{aligned}&\partial _{\textbf{u}}\mathcal {L}_{g, \gamma }^{k}(X^m \hat{\nabla }_m V) \\&\quad = X^m\hat{\nabla }_m \partial _{\textbf{u}}\mathcal {L}_{g, \gamma }^{k} (V) + X^m\partial _{\textbf{u}}[\mathcal {L}_{g, \gamma }^k,\hat{\nabla }_m]V + X^m[\partial _{\textbf{u}}, \hat{\nabla }_m]\mathcal {L}_{g, \gamma }^k V \\&\qquad + \partial _{\textbf{u}}X^m\cdot \mathcal {L}_{g, \gamma }^{k} (\hat{\nabla }_m V) + \partial _{\textbf{u}}[\mathcal {L}_{g, \gamma }^k, X^m] \hat{\nabla }_m V.\end{aligned}\end{aligned}$$

Thus

7.3 $$\begin{aligned} \partial _{\textbf{u}}\mathcal {L}_{g, \gamma }^{\ell -1}(X^k \nabla _k \mathcal {L}_{g, \gamma }h_{ij}) = X^m\hat{\nabla }_m \partial _{\textbf{u}}\mathcal {L}_{g, \gamma }^{\ell }( h_{ij} ) + \mathcal {R}_{ij}^1 + \mathcal {R}_{ij}^2, \end{aligned}$$

where

$$\begin{aligned}\begin{aligned} \mathcal {R}_{ij}^1&:=\partial _{\textbf{u}}\mathcal {L}_{g, \gamma }^{\ell -1}(X^m \Upsilon ^k_{mi} \mathcal {L}_{g, \gamma }h_{kj}) + \partial _{\textbf{u}}\mathcal {L}_{g, \gamma }^{\ell -1}(X^m \Upsilon ^k_{mj} \mathcal {L}_{g, \gamma }h_{ki})\\&\quad + X^m\partial _{\textbf{u}}[\mathcal {L}_{g, \gamma }^{\ell -1},\hat{\nabla }_m]\mathcal {L}_{g, \gamma }h_{ij} \\  &\quad + \partial _{\textbf{u}}([\mathcal {L}_{g, \gamma }^{\ell -1}, X^m] \hat{\nabla }_m \mathcal {L}_{g, \gamma }h_{ij} ) , \\ \mathcal {R}_{ij}^2&:=X^m[\partial _{\textbf{u}}, \hat{\nabla }_m]\mathcal {L}_{g, \gamma }^{\ell } h_{ij} + \partial _{\textbf{u}}X^m\cdot \mathcal {L}_{g, \gamma }^{\ell -1} (\hat{\nabla }_m \mathcal {L}_{g, \gamma }h_{ij}).\end{aligned}\end{aligned}$$

We integrate by parts on the first term in (7.3) using (5.6b) and Lemma 5.19 to find

$$\begin{aligned}\begin{aligned} \left| \big (X^m\hat{\nabla }_m \partial _{\textbf{u}}\mathcal {L}_{g, \gamma }^{\ell }( h),\partial _{\textbf{u}}\mathcal {L}_{g, \gamma }^{\ell }(h)\big )_{L^2_{g,\gamma }} \right|&\lesssim \Vert X \Vert _{H^3} \Vert \partial _{\textbf{u}}\mathcal {L}_{g, \gamma }^\ell h \Vert _{L^2_{g,\gamma }}^2 \lesssim \Lambda (T) {E^g_{\partial _{\textbf{u}},N-1}}.\end{aligned}\end{aligned}$$

The remaining terms $\mathcal {R}_{ij}^{1,2}$ appearing in (7.3) are errors terms. Since we work at high regularity, we can control such error terms using the basic idea of taking low-derivative terms out in $L^\infty$. We briefly present this argument once for the last term in $\mathcal {R}_{ij}^{1}$:

7.4 $$\begin{aligned} \begin{aligned}&\Vert \partial _{\textbf{u}}([\mathcal {L}_{g, \gamma }^{\ell -1}, X^m] \hat{\nabla }_m \mathcal {L}_{g, \gamma }h_{ij})\Vert _{L^2_{g,\gamma }} \\&\quad \lesssim \sum _{\begin{array}{c} |I|+|J|\le 2(\ell -1) \\ |I|\ge 1 \end{array}} \Vert \partial _{\textbf{u}}(\hat{\nabla }^I X *\hat{\nabla }^J \hat{\nabla }\mathcal {L}_{g, \gamma }h )\Vert _{L^2_{g,\gamma }} \\&\quad \lesssim \sum _{\begin{array}{c} |I|+|J|\le N-3, \\ |I|\ge 1, |J| \ge 3 \end{array}} \Vert \partial _{\textbf{u}}(\hat{\nabla }^I X) \hat{\nabla }^J h \Vert _{L^2_{g,\gamma }} + \Vert \hat{\nabla }^I X \partial _{\textbf{u}}( \hat{\nabla }^J h )\Vert _{L^2_{g,\gamma }}. \end{aligned}\end{aligned}$$

We are now faced with four terms depending on where the derivatives sit. Two of these have high derivatives on the shift *X*, and so by Sobolev embedding these are controlled by

$$\begin{aligned}\begin{aligned} \sum _{\begin{array}{c} 1 \le |I|\le N-3, \\ 3 \le |J| \le (N-3)/2 \end{array}} \big (\Vert \partial _{\textbf{u}}\hat{\nabla }^I X \Vert _{L^2} \Vert \hat{\nabla }^J h \Vert _{H^2} + \Vert \hat{\nabla }^I X \Vert _{L^2} \Vert \partial _{\textbf{u}}\hat{\nabla }^J h \Vert _{H^2} \big ).\end{aligned}\end{aligned}$$

We use Lemma 6.6 to exchange the $H^2$ norm with the $\partial _{\textbf{u}}$ derivative, and then all terms are then controlled using Corollary 6.5 and Lemma 5.6 provided $\frac{N-3}{2}+2\le N-1$. The other two terms, where the high derivatives hit the metric, are similarly controlled by:

$$\begin{aligned}\begin{aligned} \sum _{\begin{array}{c} 3 \le |J|\le N-1, \\ 1 \le |I| \le (N-3)/2 \end{array}} \big (\Vert \partial _{\textbf{u}}\hat{\nabla }^I X \Vert _{H^2} \Vert \hat{\nabla }^J h \Vert _{L^2} + \Vert \hat{\nabla }^I X \Vert _{H^2} \Vert \partial _{\textbf{u}}\hat{\nabla }^J h \Vert _{L^2} \big ) \end{aligned}\end{aligned}$$

and once again all these terms are controlled using Lemma 5.6, Corollary 6.5 and Lemma 6.6 provided $\frac{N-3}{2}+2\le N-1$.

Carrying on this way, and using the commutator estimates contained in Lemma 5.14, together with Corollary 6.5, we find

$$\begin{aligned}\begin{aligned} \Big |\big (\mathcal {R}^1,\partial _{\textbf{u}}\mathcal {L}_{g, \gamma }^{\ell }(h)\big )_{L^2_{g,\gamma }} \Big | \lesssim \Lambda (T) {E^g_{\partial _{\textbf{u}},N-1}}+ \Lambda (T) E^g_{N-1} + \Lambda (T)^2 ({E^g_{\partial _{\textbf{u}},N-1}})^{1/2}.\end{aligned}\end{aligned}$$

For the other error term, $\mathcal {R}^2$, we use Lemma 5.6, Lemma 5.14, Corollary 6.4 and Corollary 6.5, to find

$$\begin{aligned}\begin{aligned}&\Big |\big (\mathcal {R}^2,\partial _{\textbf{u}}\mathcal {L}_{g, \gamma }^{\ell }(h)\big )_{L^2_{g,\gamma }} \Big | \\&\quad \lesssim \Vert X\Vert _{H^2} \Vert \partial _{\textbf{u}}\mathcal {L}_{g, \gamma }^{\ell }(h)\Vert _{L^2_{g,\gamma }} \big ( \varepsilon e^{(-1+\mu ) T} \Vert g-\gamma \Vert _{H^{N}} +\Lambda (T) \Vert \partial _T h \Vert _{H^{N-1}} \big ) \\  &\qquad + \Vert \partial _{\textbf{u}}X\Vert _{H^2} \Vert g-\gamma \Vert _{H^{N}} \Vert \partial _{\textbf{u}}\mathcal {L}_{g, \gamma }^\ell h \Vert _{L^2_{g,\gamma }} \\&\quad \lesssim \Lambda (T)\Big ( ({E^g_{\partial _{\textbf{u}},N-1}})^{1/2} + (E^g_{N-1})^{1/2} + \Lambda (T)\Big ) ({E^g_{\partial _{\textbf{u}},N-1}})^{1/2}. \end{aligned}\end{aligned}$$

The second term in the RHS of (7.1) is estimated by the same expression as for $\mathcal {R}^1$. For the third term in the RHS of (7.1), we use the definition of $\mathcal {F}^X$ given in (2.7b):

$$\begin{aligned}&\partial _{\textbf{u}}\mathcal {L}_{g, \gamma }^{\ell -1}(\nabla _j \mathcal {F}^X_i) \\&\quad = \partial _{\textbf{u}}\mathcal {L}_{g, \gamma }^{\ell -1}\nabla _j \left( 2 \hat{\nabla }_c N \Sigma ^c_i - \hat{\nabla }_i \widehat{N}+ 2 N \tau ^2 g_{ai}\jmath ^a - g_{ai}(2N \Sigma ^{bc} - \nabla ^b X^c)\Upsilon ^a_{bc} \right) . \end{aligned}$$

The second term in the large brackets here ($\hat{\nabla }_i \widehat{N}$) decays the slowest, while from a regularity point of view the most subtle term is the matter term ($\jmath ^a$). For this latter term, we commute in the $\partial _{\textbf{u}}$ operator and use the matter estimates of Lemma 4.4, Lemma 5.10 and Lemma 5.12 together with the commutator estimates of Lemma 5.13 and Corollary 5.15:

$$\begin{aligned}\begin{aligned}&\Vert \partial _{\textbf{u}}\mathcal {L}_{g, \gamma }^{\ell -1}\nabla _j (\tau ^2 g_{ai}\jmath ^a)\Vert _{L^2} \\&\quad \lesssim \tau ^2 \big (\Vert \partial _{\textbf{u}}\jmath ^a\Vert _{H^{N-2}} + \Vert [\partial _{\textbf{u}}, \mathcal {L}_{g, \gamma }^{\ell -1}]\hat{\nabla }_j \jmath ^a \Vert _{L^2} +\Vert [\partial _{\textbf{u}},\nabla _j] \jmath ^a\Vert _{H^{N-3}}\big ) \\&\quad \lesssim \tau ^2 \big (\Vert \partial _{\textbf{u}}\jmath ^a\Vert _{H^{N-2}} + \varepsilon e^{\max \{-1+\mu ,-\lambda \} T} \Vert \jmath \Vert _{H^{N-2}} +\Lambda (T)\Vert \partial _T \jmath \Vert _{H^{N-3}}\big ) \\&\quad \lesssim \varepsilon ^2 e^{(-\lambda +\mu )T} \Lambda (T) + \Lambda (T)^2 + \varepsilon ^4 e^{-(3+\delta )T}.\end{aligned}\end{aligned}$$

All together, and using Lemma 5.19, we find

$$\begin{aligned}&\Big |\big (2 \partial _{\textbf{u}}\mathcal {L}_{g, \gamma }^{\ell -1}\nabla _{(a} \mathcal {F}^X_{b)},\partial _{\textbf{u}}\mathcal {L}_{g, \gamma }^{\ell }(h_{ab})\big )_{L^2_{g,\gamma }} \Big |\\  &\quad \lesssim \Lambda (T) {E^g_{\partial _{\textbf{u}},N-1}}+ \Lambda (T) E^g_{N-1} \\  &\qquad + \Big (\Lambda (T)^2 + \varepsilon ^2 e^{(-\lambda +\mu )T} \Lambda (T) + \varepsilon ^4 e^{-(3+\delta )T} \Big ) ({E^g_{\partial _{\textbf{u}},N-1}})^{1/2}.\end{aligned}$$

Finally, we have

$$\begin{aligned}\begin{aligned} \Vert \partial _{\textbf{u}}\mathcal {L}_{g, \gamma }^{\ell -1} E\Vert _{L^2}&\lesssim \sum _{\begin{array}{c} |I|+|J|\le N-2 \\ |J|\ge 1 \end{array}} \Vert \partial _{\textbf{u}}(\hat{\nabla }^I {{\,\textrm{Riem}\,}}*\hat{\nabla }^J X )\Vert _{L^2_{g,\gamma }} \lesssim \Lambda (T),\end{aligned}\end{aligned}$$

where the Riemann terms can be estimated using arguments as in the proof of Proposition 7.3. $\square$

## Geometric Energy Estimates

In this section we establish energy estimates for the geometric energy functionals. We first prove estimates for the time-derivatives of the lower-order energy functional ${E^g}_{N-1}$, and then for the top-order energy functional $\mathcal {E}^g_{\partial _{\textbf{u}},N-1}$. Recall $\alpha , c_E$ and $E^g_{tot}$ are given in Definitions 3.8 and 3.10.

### Proposition 8.1

There exists a constant $C>0$ such that

$$\begin{aligned} \partial _T {E^g}_{N-1} \le - 2 \alpha {E^g}_{N-1} + C \Lambda (T) ({E^g}_{N-1})^{1/2} + C({E^g}_{N-1})^{3/2}. \end{aligned}$$

### Proof

Let $0 \le k \le N-1$. Using an estimate from [1, Lemma 20] together with Lemma 4.4, we find

$$\begin{aligned}\begin{aligned} \partial _T {E^g}_k&\le - 2 \alpha {E^g}_k + 6 ({E^g}_k)^{1/2} |\tau | \Vert N S \Vert _{H^{k-1}} + C({E^g}_k)^{3/2}\\&\quad + C({E^g}_k)^{1/2} \big ( |\tau | \Vert \eta \Vert _{H^{k-1}} + \tau ^2 \Vert N j \Vert _{H^{k-2}} \big ) \\  &\le - 2 \alpha {E^g}_k + C({E^g}_k)^{1/2} |\tau | \Vert \rho \Vert _{H^{k-1}} + C({E^g}_k)^{3/2} + C \Lambda (T) ({E^g}_k)^{1/2} .\end{aligned}\end{aligned}$$

$$\square $$

We now turn to the time evolution of the top-order $\partial _{\textbf{u}}-$*boosted* geometric energy. The main result is stated in Theorem 8.3. For ease of presentation however, the proof relies on the subsequent estimates given in Propositions 8.4, 8.5, 8.7, 8.8, and 8.10.

### Remark 8.2

The key auxiliary estimates of the previous section, Corollary 7.5 and Proposition 7.6, are applied in Proposition 8.5 and Proposition 8.4 respectively. The important integration by parts identity (5.6c) in Lemma 5.3, where one term is brought back onto the LHS, is applied in Proposition 8.8 (see (8.10)).

### Theorem 8.3

There exists a constant $C>0$ such that

$$\begin{aligned}\begin{aligned} \partial _T \mathcal {E}^g_{\partial _{\textbf{u}},N-1}&\le -2\alpha \mathcal {E}^g_{\partial _{\textbf{u}},N-1} + C\big ( |\tau | \Vert u\Vert _{H^{N-1}}+ \Lambda (T) \big )E^g_{tot}+ C\Lambda (T) (E^g_{tot})^{1/2} \\  &\quad + C(E^g_{tot})^{3/2} + C\Lambda (T)^2.\end{aligned} \end{aligned}$$

### Proof

Recalling Definition 3.11, the energy ${E^g_{\partial _{\textbf{u}},N-1}}$ consists of a sum over lower-order energies. The top-order is the most subtle, so we focus on this and merely remark that the estimates for the lower-orders follow in the same (or possibly easier) way. The top-order energy consists of two parts, an $\mathcal {E}^g_{\partial _{\textbf{u}},N-1}$ term and a $c_E \Gamma ^g_{\partial _{\textbf{u}},N-1}$ term. We treat these separately for the moment.

Using (3.9)-(3.2) and integration by parts, we find

8.1 $$\begin{aligned} \begin{aligned} \partial _T \mathcal {E}^g_{\partial _{\textbf{u}},2\ell }(T)&\le \big ( \Vert \widehat{N}\Vert _{L^\infty }+ \Vert \nabla X\Vert _{L^\infty }\big )\mathcal {E}^g_{\partial _{\textbf{u}},2\ell }(T)\\&\quad +\Big [9\big (\partial _{\textbf{u}}\mathcal {L}_{g, \gamma }^{\ell }(\partial _T h),\partial _{\textbf{u}}\mathcal {L}_{g, \gamma }^{\ell }(h)\big )_{L^2_{g,\gamma }} \\  &\quad + \tfrac{1}{2}\big ( \partial _{\textbf{u}}\mathcal {L}_{g, \gamma }^{\ell } \partial _T v ,\partial _{\textbf{u}}\mathcal {L}_{g, \gamma }^{\ell -1} v \big )_{L^2_{g,\gamma }}\\&\quad + \tfrac{1}{2}\big ( \partial _{\textbf{u}}\mathcal {L}_{g, \gamma }^{\ell } v ,\partial _{\textbf{u}}\mathcal {L}_{g, \gamma }^{\ell -1} \partial _T v \big )_{L^2_{g,\gamma }} \Big ] + G_c,\end{aligned} \end{aligned}$$

where we define

$$\begin{aligned}\begin{aligned} G_{c}&:=9\big ([\partial _{\textbf{u}}\mathcal {L}_{g, \gamma }^{\ell }, \partial _T](h),\partial _{\textbf{u}}\mathcal {L}_{g, \gamma }^{\ell }(h)\big )_{L^2_{g,\gamma }} \\  &\quad +\tfrac{1}{2}\big ( \partial _{\textbf{u}}\mathcal {L}_{g, \gamma }^{\ell } (v) ,[\partial _{\textbf{u}}\mathcal {L}_{g, \gamma }^{\ell -1},\partial _T] (v)\big )_{L^2_{g,\gamma }} + \tfrac{1}{2}\big ([\partial _{\textbf{u}}\mathcal {L}_{g, \gamma }^{\ell },\partial _T]( v) ,\partial _{\textbf{u}}\mathcal {L}_{g, \gamma }^{\ell -1} (v) \big )_{L^2_{g,\gamma }}.\end{aligned} \end{aligned}$$

The terms $G_c$ are error terms, and so our main focus is on the terms appearing (8.1) in the square bracket. Using the equations of motion (2.8) and self-adjointness of $\mathcal {L}_{g, \gamma }$, these become

$$\begin{aligned}\begin{aligned} 9\big (\partial _{\textbf{u}}\mathcal {L}_{g, \gamma }^{\ell }(\partial _T h),\,&\partial _{\textbf{u}}\mathcal {L}_{g, \gamma }^{\ell }(h)\big )_{L^2_{g,\gamma }} + \tfrac{1}{2}\big (\partial _{\textbf{u}}\mathcal {L}_{g, \gamma }^{\ell } (\partial _T v) ,\partial _{\textbf{u}}\mathcal {L}_{g, \gamma }^{\ell -1} (v) \big )_{L^2_{g,\gamma }}\\&\quad + \tfrac{1}{2}\big (\partial _{\textbf{u}}\mathcal {L}_{g, \gamma }^{\ell } (v),\partial _{\textbf{u}}\mathcal {L}_{g, \gamma }^{\ell -1} (\partial _T v) \big )_{L^2_{g,\gamma }} \\  &= -2\big ( \partial _{\textbf{u}}\mathcal {L}_{g, \gamma }^{\ell }(v),\partial _{\textbf{u}}\mathcal {L}_{g, \gamma }^{\ell -1}(v)\big )_{L^2_{g,\gamma }} + G_1 + G_2 + G_3 + G_4,\end{aligned}\end{aligned}$$

where

$$\begin{aligned} G_1&:=9\big (\partial _{\textbf{u}}\mathcal {L}_{g, \gamma }^{\ell }(2\widehat{N}g - \mathscr {L}_X g),\partial _{\textbf{u}}\mathcal {L}_{g, \gamma }^{\ell }(h)\big )_{L^2_{g,\gamma }}, \\ G_2&:=3\big (\partial _{\textbf{u}}\mathcal {L}_{g, \gamma }^\ell (F_v),\partial _{\textbf{u}}\mathcal {L}_{g, \gamma }^{\ell -1}(v)\big )_{L^2_{g,\gamma }} +3 \big (\partial _{\textbf{u}}\mathcal {L}_{g, \gamma }^\ell (v),\partial _{\textbf{u}}\mathcal {L}_{g, \gamma }^{\ell -1}(F_v)\big )_{L^2_{g,\gamma }}, \\ G_3&:=9\Big [ \big (\partial _{\textbf{u}}\mathcal {L}_{g, \gamma }^{\ell }(wv),\partial _{\textbf{u}}\mathcal {L}_{g, \gamma }^{\ell }(h)\big )_{L^2_{g,\gamma }} \\  &\quad - \tfrac{9}{2} \Big [\big (\partial _{\textbf{u}}\mathcal {L}_{g, \gamma }^{\ell }(w\mathcal {L}_{g, \gamma }h),\partial _{\textbf{u}}\mathcal {L}_{g, \gamma }^{\ell -1}(v)\big )_{L^2_{g,\gamma }} +\big (\partial _{\textbf{u}}\mathcal {L}_{g, \gamma }^{\ell }(v),\partial _{\textbf{u}}\mathcal {L}_{g, \gamma }^{\ell -1}(w\mathcal {L}_{g, \gamma }h)\big )_{L^2_{g,\gamma }}\Big ], \\ G_4&:=\tfrac{1}{2} \big (\partial _{\textbf{u}}\mathcal {L}_{g, \gamma }^{\ell }(X^m \hat{\nabla }_m v),\partial _{\textbf{u}}\mathcal {L}_{g, \gamma }^{\ell -1}(v)\big )_{L^2_{g,\gamma }} + \tfrac{1}{2} \big (\partial _{\textbf{u}}\mathcal {L}_{g, \gamma }^{\ell }(v),\partial _{\textbf{u}}\mathcal {L}_{g, \gamma }^{\ell -1}(X^m \hat{\nabla }_m v)\big )_{L^2_{g,\gamma }}. \end{aligned}$$

Similarly using (3.9)-(3.2) and integration by parts, we find

8.2 $$\begin{aligned} \begin{aligned} \partial _T \Gamma ^g_{\partial _{{\textbf {u}}},2\ell }(T)&\le \varepsilon e^{-T} \Gamma ^g_{\partial _{{\textbf {u}}},2\ell }(T) +\big (\partial _{{\textbf {u}}}\mathcal {L}_{g, \gamma }^{\ell -1}(\partial _T v),\partial _{{\textbf {u}}}\mathcal {L}_{g, \gamma }^{\ell }(h)\big )_{L^2_{g,\gamma }}\\  &+ \big ( \partial _{{\textbf {u}}}\mathcal {L}_{g, \gamma }^{\ell -1} v ,\partial _{{\textbf {u}}}\mathcal {L}_{g, \gamma }^{\ell } \partial _T h\big )_{L^2_{g,\gamma }} + G_{cc} \,,\end{aligned}\end{aligned}$$

where we define

$$\begin{aligned}\begin{aligned} G_{cc}&:=\big ([\partial _T, \partial _{\textbf{u}}\mathcal {L}_{g, \gamma }^{\ell -1}](v),\partial _{\textbf{u}}\mathcal {L}_{g, \gamma }^{\ell }(h)\big )_{L^2_{g,\gamma }} +\big ( \partial _{\textbf{u}}\mathcal {L}_{g, \gamma }^{\ell -1} (v) ,[\partial _T,\partial _{\textbf{u}}\mathcal {L}_{g, \gamma }^{\ell }] (h)\big )_{L^2_{g,\gamma }}.\end{aligned}\end{aligned}$$

Using the equations of motion (2.8)

$$\begin{aligned}\begin{aligned}\big (&\partial _{{\textbf {u}}}\mathcal {L}_{g, \gamma }^{\ell -1}(\partial _T v),\partial _{{\textbf {u}}}\mathcal {L}_{g, \gamma }^{\ell }(h)\big )_{L^2_{g,\gamma }} + \big ( \partial _{{\textbf {u}}}\mathcal {L}_{g, \gamma }^{\ell -1} v ,\partial _{{\textbf {u}}}\mathcal {L}_{g, \gamma }^{\ell } \partial _T h\big )_{L^2_{g,\gamma }} \\  &= -2\big ( \partial _{{\textbf {u}}}\mathcal {L}_{g, \gamma }^{\ell -1}(v),\partial _{{\textbf {u}}}\mathcal {L}_{g, \gamma }^{\ell }(h)\big )_{L^2_{g,\gamma }} - 9\big ( \partial _{{\textbf {u}}}\mathcal {L}_{g, \gamma }^{\ell }(h),\partial _{{\textbf {u}}}\mathcal {L}_{g, \gamma }^{\ell }(h)\big )_{L^2_{g,\gamma }}\\  &\quad + \big ( \partial _{{\textbf {u}}}\mathcal {L}_{g, \gamma }^{\ell -1}(v),\partial _{{\textbf {u}}}\mathcal {L}_{g, \gamma }^{\ell }(v)\big )_{L^2_{g,\gamma }} \\  &\quad + G_5 + G_6 + G_7 + G_8 + G_9,\end{aligned}\end{aligned}$$

where we have defined

$$\begin{aligned} G_5&:=-9\big (\partial _{\textbf{u}}\mathcal {L}_{g, \gamma }^{\ell -1}(\widehat{N}\mathcal {L}_{g, \gamma }h),\partial _{\textbf{u}}\mathcal {L}_{g, \gamma }^{\ell }(h)\big )_{L^2_{g,\gamma }}, \quad&G_6&:=\big ( \partial _{\textbf{u}}\mathcal {L}_{g, \gamma }^{\ell -1}(v),\partial _{\textbf{u}}\mathcal {L}_{g, \gamma }^{\ell }(\widehat{N}v)\big )_{L^2_{g,\gamma }}, \\ G_7&:=\big (\partial _{\textbf{u}}\mathcal {L}_{g, \gamma }^{\ell -1}(v),\partial _{\textbf{u}}\mathcal {L}_{g, \gamma }^{\ell }(2\widehat{N}g - \mathscr {L}_X g)\big )_{L^2_{g,\gamma }}, \quad&G_8&:=- \big (\partial _{\textbf{u}}\mathcal {L}_{g, \gamma }^{\ell -1}(X^m \hat{\nabla }_m v),\partial _{\textbf{u}}\mathcal {L}_{g, \gamma }^{\ell }(h)\big )_{L^2_{g,\gamma }}, \\ G_9&:=6\big (\partial _{\textbf{u}}\mathcal {L}_{g, \gamma }^{\ell -1}(F_v),\partial _{\textbf{u}}\mathcal {L}_{g, \gamma }^{\ell }(h),\big )_{L^2_{g,\gamma }}. \end{aligned}$$

The errors terms are estimated in the following Sects. 8.1 and 8.2: $G_1$ and $G_7$ (Proposition 8.4), $G_2$ and $G_9$ (Proposition 8.5), $G_3$, $G_5$ and $G_6$ (Proposition 8.7), $G_4$ and $G_8$ (Proposition 8.8), $G_c$ and $G_{cc}$ (Proposition 8.10). Using these estimates, and adding (8.1) and $c_E \times$(8.2) together, yields the required inequality. $\square$

### Estimates on $\mathbf {G_1}$ to $\mathbf {G_9}$

In this section we prove estimates on the terms $G_1$ to $G_9$. We begin with a useful identity. Let ${\tilde{V}}_{ij},{\tilde{P}}_{ij}$ be symmetric (0, 2)-tensors on *M* and $k \in \mathbb {Z}_{\ge 1}$. Then, the integration by parts rule (5.6a) implies

8.3 $$\begin{aligned} \begin{aligned} \big (\partial _{\textbf{u}}&\mathcal {L}_{g, \gamma }^{k}({\tilde{V}} ),\partial _{\textbf{u}}\mathcal {L}_{g, \gamma }^{k-1}({\tilde{P}})\big )_{L^2_{g,\gamma }} \\  &= \big (g^{ab}\hat{\nabla }_a\partial _{\textbf{u}}\mathcal {L}_{g, \gamma }^{k-1}(\tilde{V}),\hat{\nabla }_b\partial _{\textbf{u}}\mathcal {L}_{g, \gamma }^{k-1}({\tilde{P}})\big )_{L^2_{g,\gamma }}\\&\quad - 2\big (\text {Riem}[\gamma ]\circ \partial _{\textbf{u}}\mathcal {L}_{g, \gamma }^{k-1}(\tilde{V}),\partial _{\textbf{u}}\mathcal {L}_{g, \gamma }^{k-1}({\tilde{P}})\big )_{L^2_{g,\gamma }} \\  &\quad +\big ([\partial _{\textbf{u}},\mathcal {L}_{g, \gamma }] \mathcal {L}_{g, \gamma }^{k-1}({\tilde{V}}),\partial _{\textbf{u}}\mathcal {L}_{g, \gamma }^{k-1}(\tilde{P})\big )_{L^2_{g,\gamma }}.\end{aligned}\end{aligned}$$

#### Proposition 8.4

We have,

$$\begin{aligned} |G_1|+|G_7|\lesssim \Lambda (T){E^g_{\partial _{\textbf{u}},N-1}}+ \Lambda (T) E^g_{N-1} + \Lambda (T) ({E^g_{\partial _{\textbf{u}},N-1}})^{1/2} + \Lambda (T)^2. \end{aligned}$$

#### Proof

The first term in $G_1$ is easily controlled using Lemma 5.19 and Corollary 6.5:

$$\begin{aligned}\begin{aligned} \big |\big (\partial _{\textbf{u}}\mathcal {L}_{g, \gamma }^{\ell }(2\widehat{N}g),\partial _{\textbf{u}}\mathcal {L}_{g, \gamma }^{\ell }(h)\big )_{L^2_{g,\gamma }} \big |&\lesssim \sum _{|I|\le N-1} \Vert \partial _{\textbf{u}}\hat{\nabla }^I \widehat{N}\Vert _{L^2_{g,\gamma }} \cdot \Vert \partial _{\textbf{u}}\mathcal {L}_{g, \gamma }^\ell h \Vert _{L^2_{g,\gamma }}\\&\lesssim \Lambda (T) ({E^g_{\partial _{\textbf{u}},N-1}})^{1/2}.\end{aligned}\end{aligned}$$

For the Lie derivative term in $G_1$, we rewrite it as

8.4 $$\begin{aligned} \partial _{\textbf{u}}\mathcal {L}_{g, \gamma }^{\ell }(\mathscr {L}_X g_{ij}) = \partial _{\textbf{u}}\mathcal {L}_{g, \gamma }^{\ell -1}(\Delta \mathscr {L}_X g_{ij} + \partial _{\textbf{u}}\mathcal {L}_{g, \gamma }^{\ell -1}\big ( \mathcal {L}_{g, \gamma }- \Delta \big ) \mathscr {L}_X g_{ij}). \end{aligned}$$

The first term on the RHS of (8.4) is precisely what is controlled using Proposition 7.6. For the second term, we schematically have

$$\begin{aligned}\begin{aligned} (\mathcal {L}_{g, \gamma }- \Delta ) \mathscr {L}_X g&= g^{-1}(\hat{\nabla }\hat{\nabla }- \nabla \nabla )\mathscr {L}_X g + \text {Riem}[\gamma ] *\mathscr {L}_X g \\  &= \nabla \Upsilon *\nabla X + \Upsilon *\nabla \nabla X + \Upsilon *\Upsilon *\nabla X + \nabla X.\end{aligned}\end{aligned}$$

Using the $\Upsilon$ estimate (5.5), we have

$$\begin{aligned}\begin{aligned}&\big |\big (\partial _{\textbf{u}}\mathcal {L}_{g, \gamma }^{\ell -1}((\mathcal {L}_{g, \gamma }-\Delta )\mathscr {L}_X g),\partial _{\textbf{u}}\mathcal {L}_{g, \gamma }^{\ell }(h)\big )_{L^2_{g,\gamma }}\big | \\&\quad \lesssim \sum _{|I|+|J| \le N-1} \Vert \partial _{\textbf{u}}( \hat{\nabla }^I X \hat{\nabla }^J h) \Vert _{L^2_{g,\gamma }} \cdot \Vert \partial _{\textbf{u}}\mathcal {L}_{g, \gamma }^\ell h \Vert _{L^2_{g,\gamma }} \\&\quad \lesssim \Lambda (T) \Big ( (E^g_{tot})^{1/2}+ \Lambda (T)\Big ) ({E^g_{\partial _{\textbf{u}},N-1}})^{1/2} + \Lambda (T) ({E^g_{\partial _{\textbf{u}},N-1}})^{1/2}.\end{aligned}\end{aligned}$$

where in the final estimate we used the same ideas as in (7.4), namely an application of Corollary 6.5, Lemma 6.6 and the fact that $2(\ell -1)=N-3$. The same arguments clearly apply to $G_7$ also. $\square$

#### Proposition 8.5

We have,

$$\begin{aligned}\begin{aligned} |G_2|+|G_9|\lesssim (E^g_{tot})^{3/2} +\Lambda (T) ({E^g_{\partial _{\textbf{u}},N-1}})^{1/2} +\Lambda (T)^2.\end{aligned}\end{aligned}$$

#### Proof

The two terms in $G_2$ are estimated in virtually the same way. We discuss how to estimate the second term, since it is slightly more difficult. We first use the identity (8.3) with $\tilde{V} = \Sigma$ and ${\tilde{P}} = F_v$, in order to transfer derivatives between terms:

$$\begin{aligned}\begin{aligned} \big (\partial _{{\textbf {u}}}&\mathcal {L}_{g, \gamma }^\ell (\Sigma ),\partial _{{\textbf {u}}}\mathcal {L}_{g, \gamma }^{\ell -1}(F_v)\big )_{L^2_{g,\gamma }} X\\  &= \big ( g^{ab} \hat{\nabla }_a \partial _{{\textbf {u}}}\mathcal {L}_{g, \gamma }^{\ell -1} \Sigma , \hat{\nabla }_b \partial _{{\textbf {u}}}\mathcal {L}_{g, \gamma }^{\ell -1}(F_v)\big )_{L^2_{g,\gamma }} \\  &\quad -2\big ( \text{ Riem }[\gamma ]\circ \partial _{{\textbf {u}}}\mathcal {L}_{g, \gamma }^{\ell -1}\Sigma , \partial _{{\textbf {u}}}\mathcal {L}_{g, \gamma }^{\ell -1} F_v \big )_{L^2_{g,\gamma }} \\  &\quad + \big ( [\partial _{{\textbf {u}}},\mathcal {L}_{g, \gamma }]\mathcal {L}_{g, \gamma }^{\ell -1} \Sigma , \partial _{{\textbf {u}}}\mathcal {L}_{g, \gamma }^{\ell -1}(F_v)\big )_{L^2_{g,\gamma }}.\end{aligned}\end{aligned}$$

We estimate each of these three expressions in turn. For the first expression, we use the various $F_v$ estimates from Lemma 5.5, Lemma 5.22 and Corollary 7.5, together with the commutator estimates from Lemma 5.14 and Lemma 6.6, to find

$$\begin{aligned}\begin{aligned} \Vert \partial _{\textbf{u}}\mathcal {L}_{g, \gamma }^{\ell -1}(F_v) \Vert _{H^1}&\lesssim \sum _{|I|\le 1} \Vert \partial _{\textbf{u}}\nabla ^I \mathcal {L}_{g, \gamma }^{\ell -1}(F_v) \Vert _{L^2} + \varepsilon e^{\max \{-1+\mu ,-\lambda \} T}\Vert F_v\Vert _{H^{N-2}} \\  &\quad +\Lambda (T) \Vert \partial _T F_v \Vert _{H^{N-3}} + \Lambda (T)\Vert [\partial _T, \mathcal {L}_{g, \gamma }^{\ell -1}](F_v)\Vert _{L^2} \\  &\lesssim \Lambda (T) + {E^g_{\partial _{\textbf{u}},N-1}}+E^g_{N-1} + \varepsilon e^{\max \{-1+\mu ,-\lambda \} T}\\&\quad \Big ( \Lambda (T) + \Vert g-\gamma \Vert _{H^N}^2 + \Vert \Sigma \Vert _{H^{N-1}}^2\Big ) \\  &\lesssim \Lambda (T) + {E^g_{\partial _{\textbf{u}},N-1}}+E^g_{N-1}.\end{aligned}\end{aligned}$$

Note in the final line above we used Corollary 6.4.

In addition, combining Corollary 5.16 with Corollary 6.4, we have

$$\begin{aligned}\begin{aligned} \Vert \partial _{\textbf{u}}\mathcal {L}_{g, \gamma }^{\ell -1}(\Sigma )\Vert _{H^1}&\lesssim \Vert \Sigma \Vert _{H^{N-1}}+ \Vert g-\gamma \Vert _{H^{N}} + \Lambda (T)\\&\lesssim ({E^g_{\partial _{\textbf{u}},N-1}})^{1/2} + (E^g_{N-1} )^{1/2} +\Lambda (T),\end{aligned}\end{aligned}$$

so we find that

$$\begin{aligned}\begin{aligned} \big |\big ( g^{ab} \hat{\nabla }_a \partial _{\textbf{u}}\mathcal {L}_{g, \gamma }^{\ell -1} \Sigma , \hat{\nabla }_b \partial _{\textbf{u}}\mathcal {L}_{g, \gamma }^{\ell -1}(F_v)\big )_{L^2_{g,\gamma }}\big | \lesssim (E^g_{tot})^{3/2}+\Lambda (T)^2.\end{aligned}\end{aligned}$$

In a similar way, using Corollary 5.16 and the coercive lower-order estimate from Lemma 4.5,

$$\begin{aligned}\begin{aligned} \big |\big ( \text {Riem}[\gamma ]\circ \partial _{\textbf{u}}\mathcal {L}_{g, \gamma }^{\ell -1}\Sigma , \partial _{\textbf{u}}\mathcal {L}_{g, \gamma }^{\ell -1} F_v \big )_{L^2_{g,\gamma }}\big |&\lesssim \Vert \partial _{\textbf{u}}\mathcal {L}_{g, \gamma }^{\ell -1}\Sigma \Vert _{L^2} \Vert \partial _{\textbf{u}}\mathcal {L}_{g, \gamma }^{\ell -1}(F_v) \Vert _{L^2} \\  &\lesssim (E^g_{tot})^{3/2}+\Lambda (T)^2.\end{aligned}\end{aligned}$$

Combining (5.21) with Corollary 6.4 gives

8.5 $$\begin{aligned} \begin{aligned} \Vert [\partial _{\textbf{u}},\mathcal {L}_{g, \gamma }]\mathcal {L}_{g, \gamma }^{\ell -1}\Sigma \Vert _{L^2}&\lesssim \varepsilon e^{\max \{-1+\mu ,-\lambda \} T}\big (\Vert \Sigma \Vert _{H^{N-1}} + \Vert g-\gamma \Vert _{H^N}\big ) + \Lambda (T)^2 \\  &\lesssim \varepsilon e^{\max \{-1+\mu ,-\lambda \} T}\Big (({E^g_{\partial _{\textbf{u}},N-1}})^{1/2} + (E^g_{N-1} )^{1/2}+ \Lambda (T)\Big ).\end{aligned} \nonumber \\ \end{aligned}$$

Then this gives control over the final term in $G_2$.

Finally we use the above estimates and Lemma 5.19 to estimate $G_9$ by

$$\begin{aligned} \Big |\big (\partial _{\textbf{u}}\mathcal {L}_{g, \gamma }^{\ell -1}(F_v),\partial _{\textbf{u}}\mathcal {L}_{g, \gamma }^{\ell }(h),\big )_{L^2_{g,\gamma }}\Big |&\lesssim \Vert \partial _{\textbf{u}}\mathcal {L}_{g, \gamma }^{\ell -1}(F_v) \Vert _{L^2}\Vert \partial _{\textbf{u}}\mathcal {L}_{g, \gamma }^{\ell }(h)\Vert _{L^2} \\&\lesssim \Lambda (T) ({E^g_{\partial _{\textbf{u}},N-1}})^{1/2}. \end{aligned}$$

$$\square $$

#### Remark 8.6

In the next proposition, it is crucial that the three terms of $G_3$ are treated together, since there is an important cancellation that appears.

#### Proposition 8.7

We have,

$$\begin{aligned}\begin{aligned} |G_3|+|G_5|+|G_6|&\lesssim \big ( |\tau | \Vert u\Vert _{H^{N-1}}+ \Lambda (T) \big )E^g_{tot}+ \Lambda (T) ({E^g_{\partial _{\textbf{u}},N-1}})^{1/2} \\  &\quad +(E^g_{tot})^{3/2}+ \Lambda (T)^2.\end{aligned}\end{aligned}$$

#### Proof

First note from (3.1) that

$$\begin{aligned}\begin{aligned} \big (\partial _{\textbf{u}}\mathcal {L}_{g, \gamma }^{\ell }(w \mathcal {L}_{g, \gamma }h),\partial _{\textbf{u}}\mathcal {L}_{g, \gamma }^{\ell -1}(v)\big )_{L^2_{g,\gamma }}&= \big (\partial _{\textbf{u}}\mathcal {L}_{g, \gamma }^{\ell -1}(w \mathcal {L}_{g, \gamma }h),\partial _{\textbf{u}}\mathcal {L}_{g, \gamma }^{\ell }(v)\big )_{L^2_{g,\gamma }} + G_3^e ,\end{aligned}\end{aligned}$$

where we have defined

$$\begin{aligned}&G_3^e :=\big ([\partial _{\textbf{u}},\mathcal {L}_{g, \gamma }]\mathcal {L}_{g, \gamma }^{\ell -1}(w \mathcal {L}_{g, \gamma }h),\partial _{\textbf{u}}\mathcal {L}_{g, \gamma }^{\ell -1}(v)\big )_{L^2_{g,\gamma }}\\&\quad +\big (\partial _{\textbf{u}}\mathcal {L}_{g, \gamma }^{\ell -1}(w \mathcal {L}_{g, \gamma }h),[\partial _{\textbf{u}},\mathcal {L}_{g, \gamma }]\mathcal {L}_{g, \gamma }^{\ell -1}(v)\big )_{L^2_{g,\gamma }}. \end{aligned}$$

A computation then gives,

$$\begin{aligned}\begin{aligned} \tfrac{1}{9} G_3&= \big (\partial _{\textbf{u}}\mathcal {L}_{g, \gamma }^{\ell }(wv),\partial _{\textbf{u}}\mathcal {L}_{g, \gamma }^{\ell }(h)\big )_{L^2_{g,\gamma }} -\big (\partial _{\textbf{u}}\mathcal {L}_{g, \gamma }^{\ell -1}(w \mathcal {L}_{g, \gamma }h),\partial _{\textbf{u}}\mathcal {L}_{g, \gamma }^{\ell }(v)\big )_{L^2_{g,\gamma }} -\tfrac{1}{2} G_3^e.\end{aligned} \end{aligned}$$

The worst term above occurs when all the derivatives hit the geometric variables *v*, *h* instead of the lapse variable *w*. However, crucially, such terms cancel

$$\begin{aligned} \big (w \partial _{\textbf{u}}\mathcal {L}_{g, \gamma }^{\ell }(v),\partial _{\textbf{u}}\mathcal {L}_{g, \gamma }^{\ell }(h)\big )_{L^2_{g,\gamma }} -\big (w\partial _{\textbf{u}}\mathcal {L}_{g, \gamma }^{\ell -1}( \mathcal {L}_{g, \gamma }h),\partial _{\textbf{u}}\mathcal {L}_{g, \gamma }^{\ell }(v)\big )_{L^2_{g,\gamma }} =0. \end{aligned}$$

We are left to consider

8.6 $$\begin{aligned} \begin{aligned}&\big (\partial _{\textbf{u}}\mathcal {L}_{g, \gamma }^{\ell }(N \Sigma ),\partial _{\textbf{u}}\mathcal {L}_{g, \gamma }^{\ell }(h)\big )_{L^2_{g,\gamma }} -\big (\partial _{\textbf{u}}\mathcal {L}_{g, \gamma }^{\ell -1}(N \mathcal {L}_{g, \gamma }h),\partial _{\textbf{u}}\mathcal {L}_{g, \gamma }^{\ell }(\Sigma )\big )_{L^2_{g,\gamma }} \\  &\quad = \big (\partial _{\textbf{u}}\mathcal {L}_{g, \gamma }^{\ell -1}([\mathcal {L}_{g, \gamma },N] \Sigma ),\partial _{\textbf{u}}\mathcal {L}_{g, \gamma }^{\ell }(h)\big )_{L^2_{g,\gamma }}\\&\qquad -\big (\partial _{\textbf{u}}\mathcal {L}_{g, \gamma }^{\ell }(\Sigma ),\partial _{\textbf{u}}\mathcal {L}_{g, \gamma }^{\ell -2}([\mathcal {L}_{g, \gamma },N] \mathcal {L}_{g, \gamma }h)\big )_{L^2_{g,\gamma }}.\end{aligned} \end{aligned}$$

For the first term on the RHS of (8.6),

$$\begin{aligned}\begin{aligned}&\Big | \big (\partial _{\textbf{u}}\mathcal {L}_{g, \gamma }^{\ell -1}([\mathcal {L}_{g, \gamma },N] \Sigma ),\partial _{\textbf{u}}\mathcal {L}_{g, \gamma }^{\ell }(h)\big )_{L^2_{g,\gamma }} \Big | \\&\quad \lesssim \sum _{\begin{array}{c} |I|+|J|\le 2\ell \\ |I|\ge 1 \end{array}}\Vert \partial _{\textbf{u}}( \hat{\nabla }^{I} \widehat{N}\cdot \hat{\nabla }^J \Sigma )\Vert _{L^2} \Vert \partial _{\textbf{u}}\mathcal {L}_{g, \gamma }^{\ell }(h)\Vert _{L^2} \\&\quad \lesssim \Lambda (T) \Big ( ({E^g_{\partial _{\textbf{u}},N-1}})^{1/2} + (E^g_{N-1})^{1/2} + \Lambda (T)\Big ) ({E^g_{\partial _{\textbf{u}},N-1}})^{1/2}.\end{aligned} \end{aligned}$$

For the second term on the RHS of (8.6) we need to apply the integration by parts identity (5.6a). This gives

$$\begin{aligned}\begin{aligned}&\big (\partial _{\textbf{u}}\mathcal {L}_{g, \gamma }^{\ell }(\Sigma ),\partial _{\textbf{u}}\mathcal {L}_{g, \gamma }^{\ell -2}([\mathcal {L}_{g, \gamma },N] \mathcal {L}_{g, \gamma }h)\big )_{L^2_{g,\gamma }} \\&\quad = \big (g^{ab}\hat{\nabla }_a\partial _{\textbf{u}}\mathcal {L}_{g, \gamma }^{\ell -1}(\Sigma ),\hat{\nabla }_b \partial _{\textbf{u}}\mathcal {L}_{g, \gamma }^{\ell -2}([\mathcal {L}_{g, \gamma },N] \mathcal {L}_{g, \gamma }h)\big )_{L^2_{g,\gamma }} \\&\qquad - 2 \big (\text {Riem}[\gamma ]\circ \partial _{\textbf{u}}\mathcal {L}_{g, \gamma }^{\ell -1}(\Sigma ),\partial _{\textbf{u}}\mathcal {L}_{g, \gamma }^{\ell -2}([\mathcal {L}_{g, \gamma },N] \mathcal {L}_{g, \gamma }h)\big )_{L^2_{g,\gamma }}\\&\qquad + \big ([\partial _{\textbf{u}},\mathcal {L}_{g, \gamma }]\mathcal {L}_{g, \gamma }^{\ell -1}(\Sigma ),\partial _{\textbf{u}}\mathcal {L}_{g, \gamma }^{\ell -2}([\mathcal {L}_{g, \gamma },N] \mathcal {L}_{g, \gamma }h)\big )_{L^2_{g,\gamma }},\end{aligned} \end{aligned}$$

and thus

$$\begin{aligned} \begin{aligned} \Big |&\big (\partial _{\textbf{u}}\mathcal {L}_{g, \gamma }^{\ell }(\Sigma ),\partial _{\textbf{u}}\mathcal {L}_{g, \gamma }^{\ell -2}([\mathcal {L}_{g, \gamma },N] \mathcal {L}_{g, \gamma }h)\big )_{L^2_{g,\gamma }} \Big |\\&\lesssim \Big (\Vert \partial _{\textbf{u}}\mathcal {L}_{g, \gamma }^{\ell -1} \Sigma \Vert _{H^1} + \Vert [\partial _{\textbf{u}},\mathcal {L}_{g, \gamma }]\mathcal {L}_{g, \gamma }^{\ell -1}(\Sigma )\Vert _{L^2} \Big ) \Vert \partial _{\textbf{u}}\mathcal {L}_{g, \gamma }^{\ell -2}([\mathcal {L}_{g, \gamma },N] \mathcal {L}_{g, \gamma }h)\Vert _{H^1} \\&\lesssim \Big (\Vert \partial _{\textbf{u}}\mathcal {L}_{g, \gamma }^{\ell -1} \Sigma \Vert _{H^1} + \Vert [\partial _{\textbf{u}},\mathcal {L}_{g, \gamma }]\mathcal {L}_{g, \gamma }^{\ell -1}(\Sigma )\Vert _{L^2} \Big ) \sum _{\begin{array}{c} |I|+|J|\le N-1\\ |I| \ge 1, |J| \ge 2 \end{array}} \Vert \partial _{\textbf{u}}\big ( \hat{\nabla }^I \widehat{N}\hat{\nabla }^J h\big )\Vert _{H^1}.\end{aligned} \end{aligned}$$

All these terms can be controlled by estimates in Corollary 5.16, Corollary 6.4 and (8.5), together with distributing derivatives and applying Lemma 6.6 and Corollary 5.7 as needed. We refer to the example given in (7.4). All together, we find

$$\begin{aligned} \begin{aligned} \Big |\big (&\partial _{\textbf{u}}\mathcal {L}_{g, \gamma }^{\ell }(N \Sigma ),\partial _{\textbf{u}}\mathcal {L}_{g, \gamma }^{\ell }(h)\big )_{L^2_{g,\gamma }} -\big (\partial _{\textbf{u}}\mathcal {L}_{g, \gamma }^{\ell -1}(N \mathcal {L}_{g, \gamma }h),\partial _{\textbf{u}}\mathcal {L}_{g, \gamma }^{\ell }(\Sigma )\big )_{L^2_{g,\gamma }} \Big |\\&\lesssim \Lambda (T) \Big ( {E^g_{\partial _{\textbf{u}},N-1}}+ E^g_{N-1}+ \Lambda (T)^2\Big ).\end{aligned} \end{aligned}$$

Finally, we need to estimate the terms in $G_3^e$. These in fact require the most care. For the first term in $G_3^e$, when using the commutator identity (5.11) on the first factor we see that this has potentially too-many derivatives hitting the metric *h*

$$\begin{aligned} {[}\partial _{\textbf{u}},\mathcal {L}_{g, \gamma }]\mathcal {L}_{g, \gamma }^{\ell -1}(N \mathcal {L}_{g, \gamma }h) \end{aligned}$$

Using the schematic identity given below (5.11), we see these problematic terms come when all the derivatives hit the metric *h*:

$$\begin{aligned}\begin{aligned} (\partial _{\textbf{u}}g^{-1}) *\hat{\nabla }^2 \hat{\nabla }^{N-1} h + \hat{\nabla }u^0 *\hat{\nabla }\hat{\nabla }^{N-1}\partial _T h +\tau \hat{\nabla }u^c *\hat{\nabla }^2 \hat{\nabla }^{N-1} h .\end{aligned}\end{aligned}$$

Thus for these terms we integrate by parts using (5.6b). For example, we find

$$\begin{aligned}\begin{aligned}&\Big |\Big ((\partial _{\textbf{u}}g^{ab})\hat{\nabla }_a \hat{\nabla }_b \mathcal {L}_{g, \gamma }^{\ell -1}(N \mathcal {L}_{g, \gamma }h),\partial _{\textbf{u}}\mathcal {L}_{g, \gamma }^{\ell -1}(\Sigma )\Big )_{L^2_{g,\gamma }}\Big | \\&\quad \lesssim \Vert \partial _{\textbf{u}}g\Vert _{H^3} \Vert \mathcal {L}_{g, \gamma }^\ell h \Vert _{H^{1}}\Vert \partial _{\textbf{u}}\mathcal {L}_{g, \gamma }^{\ell -1}\Sigma \Vert _{H^1}, \\&\Big |\Big ((\tau (\hat{\nabla }^a u^c) \hat{\nabla }_a \hat{\nabla }_c \mathcal {L}_{g, \gamma }^{\ell -1}(N \mathcal {L}_{g, \gamma }h),\partial _{\textbf{u}}\mathcal {L}_{g, \gamma }^{\ell -1}(\Sigma )\Big )_{L^2_{g,\gamma }}\Big | \\&\quad \lesssim |\tau | \Vert u\Vert _{H^4} \Vert \mathcal {L}_{g, \gamma }^\ell h \Vert _{H^{1}}\Vert \partial _{\textbf{u}}\mathcal {L}_{g, \gamma }^{\ell -1}\Sigma \Vert _{H^1}.\end{aligned} \end{aligned}$$

These terms can be controlled using Corollary 5.7, Corollary 5.16 and Corollary 6.4. We eventually obtain the following estimate for the first term in $G_3^e$

8.7 $$\begin{aligned} \begin{aligned}&\Big | \big ([\partial _{\textbf{u}},\mathcal {L}_{g, \gamma }]\mathcal {L}_{g, \gamma }^{\ell -1}(N \mathcal {L}_{g, \gamma }h),\partial _{\textbf{u}}\mathcal {L}_{g, \gamma }^{\ell -1}(\Sigma )\big )_{L^2_{g,\gamma }} \Big |\\&\quad \lesssim |\tau | \Vert u\Vert _{H^{N-1}}{E^g_{\partial _{\textbf{u}},N-1}}+ |\tau | \Vert u\Vert _{H^{N-1}}E^g_{N-1} \\  &\qquad + \Lambda (T) {E^g_{\partial _{\textbf{u}},N-1}}+ \Lambda (T) E^g_{N-1}+ \Lambda (T)^2.\end{aligned} \end{aligned}$$

For the second term of $G_3^e$, we use the original commutator estimate (5.12) combined with Corollary 6.4 to find

$$\begin{aligned}\begin{aligned}&\Big | \big (\partial _{\textbf{u}}\mathcal {L}_{g, \gamma }^{\ell -1}(w \mathcal {L}_{g, \gamma }h),[\partial _{\textbf{u}},\mathcal {L}_{g, \gamma }]\mathcal {L}_{g, \gamma }^{\ell -1}(v)\big )_{L^2_{g,\gamma }} \Big |\\&\quad \lesssim \Vert \partial _{\textbf{u}}\mathcal {L}_{g, \gamma }^{\ell -1}(N \mathcal {L}_{g, \gamma }h)\Vert _{L^2} \Vert [\partial _{\textbf{u}},\mathcal {L}_{g, \gamma }]\mathcal {L}_{g, \gamma }^{\ell -1}(\Sigma )\Vert _{L^2} \\  &\quad \lesssim ({E^g_{\partial _{\textbf{u}},N-1}})^{1/2} \Big (\Vert g-\gamma \Vert _{H^N}^2 + \Vert \Sigma \Vert _{H^{N-1}}^2 + \Lambda (T)^2 + |\tau | \Vert u\Vert _{H^2} \Vert \Sigma \Vert _{H^{N-1}}^2\Big ). \\  &\quad \lesssim ({E^g_{\partial _{\textbf{u}},N-1}})^{1/2} \Big ({E^g_{\partial _{\textbf{u}},N-1}}+ E^g_{N-1} + \Lambda (T)\Big ).\end{aligned} \end{aligned}$$

Finally we turn to the estimates for $G_5$ and $G_6$. The first of these is straightforward in light of previous estimates:

$$\begin{aligned}\begin{aligned} |G_5|&= \Big |\big (\partial _{\textbf{u}}\mathcal {L}_{g, \gamma }^{\ell -1}(\widehat{N}\mathcal {L}_{g, \gamma }h),\partial _{\textbf{u}}\mathcal {L}_{g, \gamma }^{\ell }(h)\big )_{L^2_{g,\gamma }}\Big |\\&\lesssim \sum _{\begin{array}{c} |I|+|J|\le N-1\\ |J| \ge 2 \end{array}} \Vert \partial _{\textbf{u}}(\hat{\nabla }^{I}\widehat{N}\hat{\nabla }^J h) \Vert _{L^2}\Vert \partial _{\textbf{u}}\mathcal {L}_{g, \gamma }^{\ell }h\Vert _{L^2} \\  &\lesssim \Lambda (T)\Big (({E^g_{\partial _{\textbf{u}},N-1}})^{1/2} + (E^g_{N-1})^{1/2} + \Lambda (T)\Big ) ({E^g_{\partial _{\textbf{u}},N-1}})^{1/2} .\end{aligned} \end{aligned}$$

For $G_6$ we need to integrate by parts once just as for the first term in $G_3^e$, however the estimate follows in the same way and so we omit the details. $\square$

#### Proposition 8.8

We have,

$$\begin{aligned} \begin{aligned} |G_4|+|G_8|&\lesssim \Lambda (T) {E^g_{\partial _{\textbf{u}},N-1}}+ \Lambda (T) E^g_{N-1}+ \Lambda (T)^2.\end{aligned} \end{aligned}$$

#### Proof

The two terms appearing in $G_4$ are

$$\begin{aligned}\begin{aligned} \big (\partial _{\textbf{u}}\mathcal {L}_{g, \gamma }^{\ell }(X^m \hat{\nabla }_m v),\partial _{\textbf{u}}\mathcal {L}_{g, \gamma }^{\ell -1}(v)\big )_{L^2_{g,\gamma }} +\big (\partial _{\textbf{u}}\mathcal {L}_{g, \gamma }^{\ell }(v),\partial _{\textbf{u}}\mathcal {L}_{g, \gamma }^{\ell -1}(X^m \hat{\nabla }_m v)\big )_{L^2_{g,\gamma }}.\end{aligned} \end{aligned}$$

We explain the estimate for the first term only since the second one follows in the same way. Using (8.3), we obtain

8.8 $$\begin{aligned} \begin{aligned}&\big (\partial _{\textbf{u}}\mathcal {L}_{g, \gamma }^{\ell }(X^m\hat{\nabla }_m \Sigma ),\partial _{\textbf{u}}\mathcal {L}_{g, \gamma }^{\ell -1}(\Sigma )\big )_{L^2_{g,\gamma }} \\&\quad = \big (g^{ab}\hat{\nabla }_a\partial _{\textbf{u}}\mathcal {L}_{g, \gamma }^{\ell -1}(X^m\hat{\nabla }_m \Sigma ),\hat{\nabla }_b\partial _{\textbf{u}}\mathcal {L}_{g, \gamma }^{\ell -1}(\Sigma )\big )_{L^2_{g,\gamma }} \\  &\qquad - 2\big (\text {Riem}[\gamma ]\circ \partial _{\textbf{u}}\mathcal {L}_{g, \gamma }^{\ell -1}(X^m\hat{\nabla }_m \Sigma ),\partial _{\textbf{u}}\mathcal {L}_{g, \gamma }^{\ell -1}(\Sigma )\big )_{L^2_{g,\gamma }} \\  &\qquad +\big ([\partial _{\textbf{u}},\mathcal {L}_{g, \gamma }] \mathcal {L}_{g, \gamma }^{\ell -1}(X^a\hat{\nabla }_a \Sigma ),\partial _{\textbf{u}}\mathcal {L}_{g, \gamma }^{\ell -1}(\Sigma )\big )_{L^2_{g,\gamma }}.\end{aligned} \end{aligned}$$

We focus on the first term on the RHS of (8.8) and use (7.2) to write it as

8.9 $$\begin{aligned} \begin{aligned} \hat{\nabla }_a&\partial _{\textbf{u}}\mathcal {L}_{g, \gamma }^{\ell -1}(X^m \hat{\nabla }_m \Sigma ) = X^m\hat{\nabla }_m \hat{\nabla }_a \partial _{\textbf{u}}\mathcal {L}_{g, \gamma }^{\ell -1} (\Sigma ) + G_4^e,\end{aligned} \end{aligned}$$

where we have introduced

$$\begin{aligned}\begin{aligned} G_4^e&:=\hat{\nabla }_a X^m \cdot \hat{\nabla }_m \partial _{\textbf{u}}\mathcal {L}_{g, \gamma }^{\ell -1} \Sigma + X^m [\hat{\nabla }_a, \hat{\nabla }_m] \partial _{\textbf{u}}\mathcal {L}_{g, \gamma }^{\ell -1} \Sigma \\  &\quad + \hat{\nabla }_a \Big [ \partial _{\textbf{u}}[\mathcal {L}_{g, \gamma }^{\ell -1} , X^m] \hat{\nabla }_m \Sigma + \partial _{\textbf{u}}\big ( X^m [\mathcal {L}_{g, \gamma }^{\ell -1} , \hat{\nabla }_m] \Sigma \big )\\&\quad + \partial _{\textbf{u}}X^m \cdot \hat{\nabla }_m \mathcal {L}_{g, \gamma }^{\ell -1} \Sigma + X^m [\partial _{\textbf{u}}, \hat{\nabla }_m] \mathcal {L}_{g, \gamma }^{\ell -1} \Sigma \Big ].\end{aligned} \end{aligned}$$

By a slight adaption of the integration by parts estimate (5.6c), we see that

8.10 $$\begin{aligned} \begin{aligned} \big | (g^{ab}X^m \hat{\nabla }_m \hat{\nabla }_a V, \hat{\nabla }_b V)_{L^2_{g,\gamma }} \big |&\lesssim \Vert X \Vert _{H^3} \Vert V\Vert _{H^1}^2.\end{aligned} \end{aligned}$$

Using this, Corollary 5.16 and Corollary 6.4, the first term of (8.9) is thus estimated as

$$\begin{aligned}\begin{aligned}&\big |\big (g^{ab}X^m \hat{\nabla }_m \hat{\nabla }_a\partial _{\textbf{u}}\mathcal {L}_{g, \gamma }^{\ell -1}(\Sigma ),\hat{\nabla }_b\partial _{\textbf{u}}\mathcal {L}_{g, \gamma }^{\ell -1}(\Sigma )\big )_{L^2_{g,\gamma }}\big | \\&\quad \lesssim \Vert X\Vert _{H^3}\Vert \partial _{\textbf{u}}\mathcal {L}_{g, \gamma }^{\ell -1} \Sigma \Vert _{H^1}^2 \\&\quad \lesssim \Lambda (T) \Big ({E^g_{\partial _{\textbf{u}},N-1}}+ E^g_{N-1} + \Lambda (T)^2\Big ).\end{aligned} \end{aligned}$$

We then turn to the remaining terms in (8.9), namely $G_4^e$. The first, second and fifth terms of $G_4^e$ are fairly straightfowardly estimated by

$$\begin{aligned}\begin{aligned}&\Vert X\Vert _{H^3} \Vert \partial _{\textbf{u}}\mathcal {L}_{g, \gamma }^{\ell -1}\Sigma \Vert _{H^1} + \Vert \partial _{\textbf{u}}X\Vert _{H^3} \Vert \mathcal {L}_{g, \gamma }^{\ell -1} \Sigma \Vert _{H^2} \\&\quad \lesssim \Lambda (T) \Big (\Vert \partial _{\textbf{u}}\mathcal {L}_{g, \gamma }^{\ell -1}\Sigma \Vert _{H^1} + \Vert \Sigma \Vert _{H^{N-1}}\Big ) \\  &\quad \lesssim \Lambda (T) \Big (({E^g_{\partial _{\textbf{u}},N-1}})^{1/2} + (E^g_{N-1})^{1/2} + \Lambda (T)\Big ).\end{aligned}\end{aligned}$$

The third and fourth terms of $G_4^e$ merely require a careful counting of derivatives. As an example, the fourth term of $G_4^e$ is estimated by

$$\begin{aligned}\begin{aligned}&\Vert \partial _{\textbf{u}}\big ( X^m [\mathcal {L}_{g, \gamma }^{\ell -1}, \hat{\nabla }_m] (\Sigma )\big )\Vert _{H^1} \\&\quad \lesssim \Vert \partial _{\textbf{u}}X\Vert _{H^3} \Vert [\mathcal {L}_{g, \gamma }^{\ell -1},\hat{\nabla }]\Sigma \Vert _{H^1} + \Vert X\Vert _{H^3} \Vert \partial _{\textbf{u}}[\mathcal {L}_{g, \gamma }^{\ell -1},\hat{\nabla }]\Sigma \Vert _{H^1}.\end{aligned}\end{aligned}$$

The first commutator term here can be studied using Lemma 5.14, in particular the same ideas as in (5.14) yield

$$\begin{aligned}\begin{aligned} \Vert [\mathcal {L}_{g, \gamma }^{\ell -1}, \hat{\nabla }_m]\Sigma \Vert _{H^1}&\lesssim \Vert g-\gamma \Vert _{H^{N-3}} \Vert \Sigma \Vert _{H^{N-2}} + \Vert \Sigma \Vert _{H^{N-4}} \lesssim (E^g_{N-1})^{1/2}.\end{aligned}\end{aligned}$$

While we use (5.13) and the same ideas in the proof of Proposition 7.3 to estimate the second commutator term by

$$\begin{aligned}\begin{aligned} \Vert \partial _{\textbf{u}}[\mathcal {L}_{g, \gamma }^{\ell -1},\hat{\nabla }]\Sigma \Vert _{H^1}&\lesssim \sum _{\begin{array}{c} |I|+|J|\le 2\ell -1\\ |I|\ge 1, |J|\ge 2 \end{array}} \Vert \partial _{\textbf{u}}(\hat{\nabla }^{I}g \hat{\nabla }^J \Sigma ) \Vert _{L^2} \\&\quad + \sum _{|I|+|J|\le 2(\ell -2)} \Vert \partial _{\textbf{u}}(\hat{\nabla }^I \text {Riem}[\gamma ]*\hat{\nabla }^J \Sigma ) \Vert _{L^2} \\  &\lesssim ({E^g_{\partial _{\textbf{u}},N-1}})^{1/2} + (E^g_{N-1})^{1/2} + \Lambda (T).\end{aligned}\end{aligned}$$

Finally, for the last term of $G_4^e$ we use the original commutator identity (5.10) to estimate it by

$$\begin{aligned}\begin{aligned}&\Vert X\Vert _{H^3} \Vert [\partial _{\textbf{u}}, \hat{\nabla }]\mathcal {L}_{g, \gamma }^{\ell -1}\Sigma \Vert _{L^2} \\&\quad \lesssim \Lambda (T) \Big ( |\tau | \Vert u\Vert _{H^3} \Vert \mathcal {L}_{g, \gamma }^{\ell -1}\Sigma \Vert _{H^1} + \Vert \hat{\nabla }u^0 \Vert _{H^2} \Vert \partial _T \Sigma \Vert _{H^{2(\ell -1)}}\Big ) \\  &\quad \lesssim \Lambda (T) \Big ( |\tau | \Vert u\Vert _{H^{N-1}} (E^g_{N-1})^{1/2} + \Lambda (T)(E^g_{N-1})^{1/2} + \Lambda (T)^2\Big ).\end{aligned}\end{aligned}$$

In summary, we obtain

$$\begin{aligned} | G_4^3| \lesssim \Lambda (T){E^g_{\partial _{\textbf{u}},N-1}}+ \Lambda (T) E^g_{N-1} + \Lambda (T)^2. \end{aligned}$$

We now turn to the second term on the RHS of (8.8), and estimate it by

$$\begin{aligned}\begin{aligned}&\Vert \partial _{\textbf{u}}\mathcal {L}_{g, \gamma }^{\ell -1}\Sigma \Vert _{L^2} \sum _{\begin{array}{c} |I|+|J|\le N-2\\ |J| \ge 1 \end{array}} \Vert \partial _{\textbf{u}}(\hat{\nabla }^{I}X^m \hat{\nabla }^J \Sigma ) \Vert _{L^2}\\&\quad \lesssim \Lambda (T){E^g_{\partial _{\textbf{u}},N-1}}+ \Lambda (T) E^g_{N-1} + \Lambda (T)^2.\end{aligned}\end{aligned}$$

We finally turn to the last term on the RHS of (8.8) and use the identity (5.11) on the first factor

$$\begin{aligned} {[}\partial _{\textbf{u}},\mathcal {L}_{g, \gamma }]\mathcal {L}_{g, \gamma }^{\ell -1}(X^a \hat{\nabla }_a \Sigma ). \end{aligned}$$

Using the schematic identity given below (5.11), we see that certain problematic terms arise when all the derivatives miss the shift and hit $\Sigma$:

$$\begin{aligned}\begin{aligned} (\partial _{\textbf{u}}g^{-1}) *X *\hat{\nabla }^2 \hat{\nabla }^{N-2} \Sigma + \hat{\nabla }u^0 *X*\hat{\nabla }\hat{\nabla }^{N-2}\partial _T \Sigma +\tau \hat{\nabla }u^c *X *\hat{\nabla }^2 \hat{\nabla }^{N-2} \Sigma .\end{aligned} \end{aligned}$$

Thus, just as for the first term in $G_3^e$ given in the proof of Proposition 8.7, we need to integrate by parts using (5.6b) on these terms. Given the close similarities, we omit the details and just state the result:

$$\begin{aligned}&\Big |\big ([\partial _{\textbf{u}},\mathcal {L}_{g, \gamma }] \mathcal {L}_{g, \gamma }^{\ell -1}(X^a\hat{\nabla }_a \Sigma ),\partial _{\textbf{u}}\mathcal {L}_{g, \gamma }^{\ell -1}(\Sigma )\big )_{L^2_{g,\gamma }}\Big | \\&\quad \lesssim \Lambda (T) {E^g_{\partial _{\textbf{u}},N-1}}+ \Lambda (T) E^g_{N-1}+ \Lambda (T)^2. \end{aligned}$$

Note, however, that the above estimate is better than in (8.7). This is because of the additional shift term appearing which always gives us additional decay (unlike the lapse which needs at least one derivative acting on it).

Finally we turn to $G_8$ and remark that

$$\begin{aligned}&\Big |\big (\partial _{\textbf{u}}\mathcal {L}_{g, \gamma }^{\ell -1}(X^m \hat{\nabla }_m v),\partial _{\textbf{u}}\mathcal {L}_{g, \gamma }^{\ell }(h)\big )_{L^2_{g,\gamma }}\Big |\\&\quad \lesssim \sum _{\begin{array}{c} |I|+|J|\le N-2\\ |J| \ge 1 \end{array}} \Vert \partial _{\textbf{u}}(\hat{\nabla }^{I}X^m \hat{\nabla }^J \Sigma ) \Vert _{L^2} \Vert \partial _{\textbf{u}}\mathcal {L}_{g, \gamma }^{\ell }h\Vert _{L^2}, \end{aligned}$$

and this is easily estimated using previous ideas. $\square$

### Estimates on $\mathbf {G_c}$ and $\mathbf {G_{cc}}$

In this final part of the section we prove estimates on the commutator error terms $G_c$ and $G_{cc}$. We first prove a preliminary lemma concerning the commutator between the operators $\partial _T$ and $\partial _{\textbf{u}}\mathcal {L}_{g, \gamma }$, and then use this lemma in the subsequent proposition.

#### Lemma 8.9

We have,

$$\begin{aligned} \begin{aligned} \Vert [\partial _{\textbf{u}}\mathcal {L}_{g, \gamma }^{\ell },\partial _T]h \Vert _{L^2} + \Vert [\partial _{\textbf{u}}\mathcal {L}_{g, \gamma }^{\ell -1},\partial _T]\Sigma \Vert _{H^1}&\lesssim {E^g_{\partial _{\textbf{u}},N-1}}+ E^g_{N-1} + \Lambda (T).\end{aligned} \end{aligned}$$

#### Proof

Let *V* be a (0, 2)-tensor on *M*. A computation yields

8.11 $$\begin{aligned} \begin{aligned} {[}\partial _T, \partial _{\textbf{u}}\mathcal {L}_{g, \gamma }]V_{ij}&= - \partial _T u^0 \cdot \mathcal {L}_{g, \gamma }\partial _T V_{ij} + \partial _T u^0 \partial _T g^{ab} \hat{\nabla }_a \hat{\nabla }_b V_{ij} - \tau u^c \hat{\nabla }_c (\mathcal {L}_{g, \gamma }V_{ij}) \\  &\quad + \tau \partial _T u^c \hat{\nabla }_c (\mathcal {L}_{g, \gamma }V_{ij}) - \partial _{\textbf{u}}(\partial _T g^{ab})\hat{\nabla }_a\hat{\nabla }_b V_{ij}\\&\quad - u^0 \partial _T g^{ab} \cdot \hat{\nabla }_a\hat{\nabla }_b \partial _T V_{ij} +\tau (\partial _T g^{ab}) u^c \hat{\nabla }_c\hat{\nabla }_a\hat{\nabla }_b V_{ij} .\end{aligned} \end{aligned}$$

Using Sobolev embedding, we find that

$$\begin{aligned}\begin{aligned} \Vert [\partial _T, \partial _{\textbf{u}}\mathcal {L}_{g, \gamma }]V \Vert _{L^2}&\lesssim \Big (\Vert \partial _T \hat{u}^0\Vert _{H^2} \Vert \partial _T h\Vert _{H^{2}}+ \Vert \partial _{\textbf{u}}\partial _T (g^{-1})\Vert _{H^2} \Big ) \Vert V\Vert _{H^{2}} \\  &\quad + |\tau |\Big (\Vert u\Vert _{H^2} + \Vert \partial _T u^c\Vert _{H^2} \Big )\Vert V\Vert _{H^{3}}\\&\quad + \Big ( \Vert \partial _T \hat{u}^0\Vert _{H^2} + \Vert \partial _T g \Vert _{H^2} \Big )\Vert \partial _T V\Vert _{H^2}.\end{aligned} \end{aligned}$$

We use (2.8) to schematically compute that

$$\begin{aligned} \partial _{\textbf{u}}\partial _T g^{-1} = \partial _{\textbf{u}}( g^{-1} g^{-1} \partial _T h )= \partial _{\textbf{u}}\Sigma +\partial _{\textbf{u}}\widehat{N}+ \partial _{\textbf{u}}(F_h) + \text {h.o.t}. \end{aligned}$$

Using Lemma 5.4, Corollary 5.7 and Corollary 5.23, we obtain

$$\begin{aligned} \Vert \partial _{\textbf{u}}\partial _T (g^{-1})\Vert _{H^2}&\lesssim \Vert \partial _{\textbf{u}}\Sigma \Vert _{H^2} +\Vert \partial _{\textbf{u}}\widehat{N}\Vert _{H^2} + \Vert \partial _{\textbf{u}}(F_h) \Vert _{H^2} + \text {h.o.t. } \\&\lesssim (E^g_{N-1})^{1/2} + \Lambda (T). \end{aligned}$$

By applying the estimates from (5.7), (5.9) and Lemma 5.6,

$$\begin{aligned} \begin{aligned} \Vert [\partial _T, \partial _{\textbf{u}}\mathcal {L}_{g, \gamma }]V \Vert _{L^2}&\lesssim \Big ( |\tau |\Vert u\Vert _{H^{2}} + |\tau |\Vert F_{u^a} \Vert _{H^{2}}+ \Lambda (T) + (E^g_{N-1})^{1/2} \Big )\Vert V\Vert _{H^{3}} \\  &\quad + \big (E^g_{N-2} + \Lambda (T)\big ) \Vert \partial _T V\Vert _{H^2} \\&\lesssim \Big ( |\tau |\Vert u\Vert _{H^{2}} + (E^g_{N-1})^{1/2} + \Lambda (T) \Big )\Vert V\Vert _{H^{3}}\\&+ \big (E^g_{N-2} + \Lambda (T)\big ) \Vert \partial _T V\Vert _{H^2}.\end{aligned}\end{aligned}$$

Thus, from Lemma 5.6, Lemma 5.14 and Corollary 6.4,

$$\begin{aligned}\begin{aligned}&\Vert [\partial _T, \partial _{{\textbf {u}}}\mathcal {L}_{g, \gamma }]\mathcal {L}_{g, \gamma }^{\ell -1} h\Vert _{L^2} \\  &\quad \lesssim \Big ( |\tau |\Vert u\Vert _{H^{2}} + (E^g_{N-1})^{1/2} + \Lambda (T) \Big )\Vert g -\gamma \Vert _{H^{N}}\\  &\qquad + \big (E^g_{N-2} + \Lambda (T)\big ) \Vert \partial _T h\Vert _{H^{N-1}}. \\  &\quad \lesssim {E^g_{\partial _{{\textbf {u}}},N-1}}+ E^g_{N-1} + \Lambda (T).\end{aligned}\end{aligned}$$

In addition, we note that at higher order $s \in \mathbb {Z}_{\ge 1}$, we have the identity

8.12 $$\begin{aligned} \begin{aligned} [\partial _T, \partial _{\textbf{u}}\mathcal {L}_{g, \gamma }^s]V_{ij}&= [\partial _T, \partial _{\textbf{u}}\mathcal {L}_{g, \gamma }](\mathcal {L}_{g, \gamma }^{s-1} V_{ij}) + (\partial _{\textbf{u}}g^{ab}) \hat{\nabla }_a \hat{\nabla }_b ([\partial _T, \mathcal {L}_{g, \gamma }^{s-1}] V_{ij}) \\  &\quad + g^{ab} \partial _{\textbf{u}}\hat{\nabla }_a \hat{\nabla }_b ([\partial _T, \mathcal {L}_{g, \gamma }^{s-1}] V_{ij}). \end{aligned}\nonumber \\ \end{aligned}$$

Using this and the commutator Lemma 5.14, we find that

$$\begin{aligned}\begin{aligned} \Vert [\partial _T, \partial _{\textbf{u}}\mathcal {L}_{g, \gamma }^\ell ] h \Vert _{L^2}&\lesssim \Vert [\partial _T, \partial _{\textbf{u}}\mathcal {L}_{g, \gamma }](\mathcal {L}_{g, \gamma }^{\ell -1} h)\Vert _{L^2} + \Vert \partial _{\textbf{u}}g \Vert _{H^2} \Vert [\partial _T, \mathcal {L}_{g, \gamma }^{\ell -1}] h\Vert _{H^2}\\&\quad + \Vert g^{ab} \partial _{\textbf{u}}\hat{\nabla }_a \hat{\nabla }_b ([\partial _T, \mathcal {L}_{g, \gamma }^{\ell -1}] h) \Vert _{L^2} \\&\lesssim {E^g_{\partial _{\textbf{u}},N-1}}+ E^g_{N-1} + \Lambda (T) + \Vert g^{ab} \partial _{\textbf{u}}\hat{\nabla }_a \hat{\nabla }_b ([\partial _T, \mathcal {L}_{g, \gamma }^{\ell -1}] h) \Vert _{L^2}.\end{aligned}\end{aligned}$$

It remains to control this last term, which we do so using the commutator identity (5.15)

$$\begin{aligned}\begin{aligned}&\Vert g^{ab}\partial _{\textbf{u}}\hat{\nabla }_a \hat{\nabla }_b \big ( [\partial _T, \mathcal {L}_{g, \gamma }^{\ell -1}]h\big ) \Vert _{L^2} \\&\quad \lesssim \sum _{i=1}^{\ell -1} \Vert g^{ab}\partial _{\textbf{u}}\hat{\nabla }_a \hat{\nabla }_b \Big ( \mathcal {L}_{g, \gamma }^{i-1} (\partial _T g^{a_i b_i})\cdot \hat{\nabla }_{a_i}\hat{\nabla }_{b_i} \big ( \mathcal {L}_{g, \gamma }^{\ell -1-i} (h)\big )\Big )\Vert _{L^2} \\  &\quad \lesssim \sum _{\begin{array}{c} |I|+|J|\le 2\ell \\ |J|\ge 2 \end{array}} \Vert \partial _{\textbf{u}}(\hat{\nabla }^{I} \partial _T g) \hat{\nabla }^{J}h\Vert _{L^2} + \Vert \hat{\nabla }^{I} \partial _T g \cdot \partial _{\textbf{u}}(\hat{\nabla }^{J}h)\Vert _{L^2} \\  &\quad \lesssim {E^g_{\partial _{\textbf{u}},N-1}}+ E^g_{N-1} + \Lambda (T)^2,\end{aligned}\end{aligned}$$

where in the final line we used (2.8) to replace $\partial _T g$ and then distributed derivatives and applied Lemma 5.6, Corollary 6.5 and Lemma 6.6 as needed.

The $\Sigma$ estimate follows in exactly the same way. $\square$

#### Proposition 8.10

We have,

$$\begin{aligned}\begin{aligned} |G_{c}| + |G_{cc}| \lesssim (E^g_{tot})^{3/2}+ \Lambda (T) ({E^g_{\partial _{\textbf{u}},N-1}})^{1/2}.\end{aligned} \end{aligned}$$

#### Proof

There are three terms to estimate in $G_c$. By Corollary 5.7 and Lemma 8.9, the first term is easily estimated as

$$\begin{aligned}\begin{aligned} \Big | \big ([\partial _{\textbf{u}}\mathcal {L}_{g, \gamma }^{\ell }, \partial _T](h),\partial _{\textbf{u}}\mathcal {L}_{g, \gamma }^{\ell }(h) \big )_{L^2_{g,\gamma }} \Big |&\lesssim \Vert [\partial _{\textbf{u}}\mathcal {L}_{g, \gamma }^{\ell }, \partial _T](h)\Vert _{L^2_{g,\gamma }}\Vert \partial _{\textbf{u}}\mathcal {L}_{g, \gamma }^{\ell }(h)\Vert _{L^2_{g,\gamma }}\\  &\lesssim (E^g_{tot})^{3/2}+ \Lambda (T) ({E^g_{\partial _{\textbf{u}},N-1}})^{1/2}.\end{aligned}\end{aligned}$$

The terms appearing in $G_{cc}$ are similarly easily estimated:

$$\begin{aligned}\begin{aligned} \Big |\big (&[\partial _T, \partial _{\textbf{u}}\mathcal {L}_{g, \gamma }^{\ell -1}](v),\partial _{\textbf{u}}\mathcal {L}_{g, \gamma }^{\ell }(h)\big )_{L^2_{g,\gamma }}\Big | +\Big |\big ( \partial _{\textbf{u}}\mathcal {L}_{g, \gamma }^{\ell -1} (v) ,[\partial _T,\partial _{\textbf{u}}\mathcal {L}_{g, \gamma }^{\ell }] (h)\big )_{L^2_{g,\gamma }}\Big | \\&\lesssim \Vert [\partial _{\textbf{u}}\mathcal {L}_{g, \gamma }^{\ell -1}, \partial _T](\Sigma )\Vert _{L^2_{g,\gamma }}\Vert \partial _{\textbf{u}}\mathcal {L}_{g, \gamma }^{\ell }(h)\Vert _{L^2_{g,\gamma }} + \Vert [\partial _{\textbf{u}}\mathcal {L}_{g, \gamma }^{\ell }, \partial _T](h)\Vert _{L^2_{g,\gamma }}\Vert \partial _{\textbf{u}}\mathcal {L}_{g, \gamma }^{\ell -1}(\Sigma )\Vert _{L^2_{g,\gamma }} \\&\lesssim (E^g_{tot})^{3/2}+ \Lambda (T) (E^g_{tot})^{1/2} + \Lambda (T)^2.\end{aligned}\end{aligned}$$

For the second term of $G_c$, we integrate it by parts using (8.3) to obtain

$$\begin{aligned}\begin{aligned}&\big (\partial _{\textbf{u}}\mathcal {L}_{g, \gamma }^{\ell } (v) ,[\partial _{\textbf{u}}\mathcal {L}_{g, \gamma }^{\ell -1},\partial _T] (v)\big )_{L^2_{g,\gamma }}\\&\quad = \big (g^{ab}\hat{\nabla }_a \partial _{\textbf{u}}\mathcal {L}_{g, \gamma }^{\ell -1} (v) ,\hat{\nabla }_b [\partial _{\textbf{u}}\mathcal {L}_{g, \gamma }^{\ell -1},\partial _T] (v)\big )_{L^2_{g,\gamma }} \\  &\qquad - 2\big ( \text {Riem}[\gamma ]\circ \partial _{\textbf{u}}\mathcal {L}_{g, \gamma }^{\ell -1} (v), [\partial _{\textbf{u}}\mathcal {L}_{g, \gamma }^{\ell -1},\partial _T] (v)\big )_{L^2_{g,\gamma }}\\&\qquad + \big ([\partial _{\textbf{u}},\mathcal {L}_{g, \gamma }] \mathcal {L}_{g, \gamma }^{\ell -1} (v) ,[\partial _{\textbf{u}}\mathcal {L}_{g, \gamma }^{\ell -1},\partial _T] (v)\big )_{L^2_{g,\gamma }}.\end{aligned}\end{aligned}$$

Thus by the estimate (5.21), Corollary 5.16, Corollary 6.4 and Lemma 8.9,

$$\begin{aligned}\begin{aligned}&\Big | \big (\partial _{\textbf{u}}\mathcal {L}_{g, \gamma }^{\ell } (v) ,[\partial _{\textbf{u}}\mathcal {L}_{g, \gamma }^{\ell -1},\partial _T] (v)\big )_{L^2_{g,\gamma }} \Big | \\&\quad \lesssim \big ( \Vert \partial _{\textbf{u}}\mathcal {L}_{g, \gamma }^{\ell -1}\Sigma \Vert _{H^1} + \Vert [\partial _{\textbf{u}}, \mathcal {L}_{g, \gamma }]\mathcal {L}_{g, \gamma }^{\ell -1}\Sigma \Vert _{L^2}\big ) \Vert [\partial _{\textbf{u}}\mathcal {L}_{g, \gamma }^{\ell }, \partial _T](\Sigma )\Vert _{H^1} \\&\quad \lesssim (E^g_{tot})^{3/2}+ \Lambda (T) (E^g_{tot})^{1/2} + \Lambda (T)^2.\end{aligned}\end{aligned}$$

For the final term of $G_c$, namely

$$\begin{aligned} \big ( [\partial _{\textbf{u}}\mathcal {L}_{g, \gamma }^{\ell },\partial _T](\Sigma ) ,\partial _{\textbf{u}}\mathcal {L}_{g, \gamma }^{\ell -1} (\Sigma ) \big )_{L^2_{g,\gamma }}, \end{aligned}$$

we need to integrate certain terms by parts since some of the factors contain too many derivatives on $\Sigma$. For example, using (8.11) and (8.12), we see that two such terms are

$$\begin{aligned} \big ( \partial _T u^0 \mathcal {L}_{g, \gamma }\partial _T (\mathcal {L}_{g, \gamma }^{\ell -1}\Sigma ) ,\partial _{\textbf{u}}\mathcal {L}_{g, \gamma }^{\ell -1} (\Sigma ) \big )_{L^2_{g,\gamma }} + \big ( \tau u^c \hat{\nabla }_c (\mathcal {L}_{g, \gamma }^\ell \Sigma ),\partial _{\textbf{u}}\mathcal {L}_{g, \gamma }^{\ell -1} (\Sigma ) \big )_{L^2_{g,\gamma }}. \end{aligned}$$

Using the integration by parts estimate (5.6b) and (5.9) we find

$$\begin{aligned}\begin{aligned} \Big |\big (\partial _T u^0 \mathcal {L}_{g, \gamma }\partial _T (\mathcal {L}_{g, \gamma }^{\ell -1}\Sigma ),\partial _{\textbf{u}}\mathcal {L}_{g, \gamma }^{\ell -1} (\Sigma ) \big )_{L^2_{g,\gamma }}\Big |&\lesssim \Vert \partial _T u^0 \Vert _{H^3} \Vert \partial _T \Sigma \Vert _{H^{N-2}} \Vert \partial _{\textbf{u}}\mathcal {L}_{g, \gamma }^{\ell -1}\Sigma \Vert _{H^1} \\  &\lesssim \Lambda E^g_{tot}+ (E^g_{tot})^2 + \Lambda (T)^3.\end{aligned}\end{aligned}$$

Similarly,

$$\begin{aligned}\begin{aligned}&\Big |\big ( \tau u^c \hat{\nabla }_c (\mathcal {L}_{g, \gamma }^\ell \Sigma ),\partial _{\textbf{u}}\mathcal {L}_{g, \gamma }^{\ell -1} (\Sigma ) \big )_{L^2_{g,\gamma }}\Big | \\&\quad \lesssim |\tau | \Vert u \Vert _{H^3} \Vert \Sigma \Vert _{H^{N-1}} \Vert \partial _{\textbf{u}}\mathcal {L}_{g, \gamma }^{\ell -1}\Sigma \Vert _{H^1} \lesssim |\tau | \Vert u \Vert _{H^{N-1}} E^g_{tot}+ \Lambda (T)^2.\end{aligned} \end{aligned}$$

All of the remaining terms can be controlled using the ideas from before. We eventually obtain

$$\begin{aligned}\begin{aligned}&\Big | \big ( [\partial _{\textbf{u}}\mathcal {L}_{g, \gamma }^{\ell },\partial _T]( v) ,\partial _{\textbf{u}}\mathcal {L}_{g, \gamma }^{\ell -1} (v) \big )_{L^2_{g,\gamma }}\Big | \\&\quad \lesssim \Big ( |\tau | \Vert u\Vert _{H^{N-1}}+ \Lambda (T) \Big )E^g_{tot}+ \Lambda (T) ({E^g_{\partial _{\textbf{u}},N-1}})^{1/2} \\  &\qquad +(E^g_{tot})^{3/2}+ \Lambda (T)^2. \end{aligned} \end{aligned}$$

$$\square $$

## Energy Estimates for the Dust Variables

In this section we establish energy estimates for the dust variables $\rho$ and $u^a$ in two propositions. An integration by parts identity, where due to symmetry a high-derivative term can be brought back onto the LHS, plays a crucial role in both propositions. We write the argument out explicitly for the first proposition, although note in principle the argument is just as in Lemma 5.3, (5.6c).

### Definition 9.1

[$\mathcal {E}_k{[}\rho {]}$] Define the functionals

$$\begin{aligned}\begin{aligned} E_k[\rho ](T)&:=\frac{1}{2} \int _M |\nabla ^k \rho (T, \cdot )|^2 \mu _g, \qquad \mathcal {E}_k[\rho ](T)&:=\sum _{0\le \ell \le k} E_\ell [\rho ](T).\end{aligned}\end{aligned}$$

### Proposition 9.2

[Time evolution of $\mathcal {E}_{N-2}{[}\rho {]}$ for fluid energy density] We have

$$\begin{aligned} |\partial _T \mathcal {E}_{N-2}[\rho ](T)| \lesssim \varepsilon e^{\max \{-1+\mu ,-\lambda \} T} \mathcal {E}_{N-2}[\rho ](T). \end{aligned}$$

### Proof

Recall from (2.7d) that the equation of motion for $\rho$ is

$$\begin{aligned}\begin{aligned} \partial _T \rho&= (u^0)^{-1}\rho F_\rho + \tau (u^0)^{-1} u^a \nabla _a \rho .\end{aligned}\end{aligned}$$

Let $3 \le k \le N-2$. Using (3.2) and the integration by parts estimate (5.6b), we have

$$\begin{aligned}\begin{aligned} \partial _T E_k[\rho ](T)&\lesssim \Vert \widehat{N}\Vert _\infty E_k[\rho ](T) + \int _M \partial _T (|\nabla ^k \rho |^2)\mu _g x+ \int _M X^c \hat{\nabla }_c (|\nabla ^k \rho |^2) \mu _g \\&\lesssim \Big ( \Vert \widehat{N}\Vert _{H^2} + \Vert X\Vert _{H^3} \Big ) E_k[\rho ](T)\\&\quad +\int _M \partial _T \Big ( g^{a_1 b_1} \cdots g^{a_k b_k}\Big ) (\nabla ^{a_1}\cdots \nabla ^{a_k}\rho )(\nabla _{a_1}\cdots \nabla _{a_k}\rho )\mu _g \\&\quad + \int _M g^{a_1 b_1} \cdots g^{a_k b_k}\Big ( \partial _T \nabla _{a_1}\cdots \nabla _{a_k}\rho \Big ) (\nabla _{b_1}\cdots \nabla _{b_k}\rho )\mu _g \\&\lesssim \Big ( \Vert \widehat{N}\Vert _{H^2} + \Vert X\Vert _{H^3} + \Vert \Sigma \Vert _{H^2} \Big ) E_k[\rho ](T) + I_k, \end{aligned} \end{aligned}$$

where we have defined that

$$\begin{aligned} I_k :=\int _M g^{a_1 b_1} \cdots g^{a_k b_k}\Big ( \partial _T \nabla _{a_1}\cdots \nabla _{a_k}\rho \Big ) (\nabla _{b_1}\cdots \nabla _{b_k}\rho )\mu _g. \end{aligned}$$

We focus on the integrand of $I_k$, commuting the derivatives, to find that

$$\begin{aligned}\begin{aligned} \partial _T \nabla _{a_1}\cdots \nabla _{a_k}\rho&= \nabla _{a_1}\cdots \nabla _{a_k}\Big ( \frac{\rho }{u^0} F_\rho + \tau \frac{u^i}{u^0} \nabla _i\rho \Big )+ [\partial _T, \nabla _{a_1}\cdots \nabla _{a_k}]\rho .\end{aligned} \end{aligned}$$

We first look at the term $\tau u^i \nabla _i\rho$ which contains the most number of derivatives on $\rho$. By commuting and integration by parts, this term becomes

$$\begin{aligned}\begin{aligned} \int _M&\tau \frac{u^i}{u^0} \big ( \nabla _{a_1}\cdots \nabla _{a_k}\nabla _i\rho \big ) (\nabla ^{a_1}\cdots \nabla ^{a_k}\rho )\mu _g \\&= \int _M \tau \frac{u^i}{u^0} \big ( [\nabla _{a_1}\cdots \nabla _{a_k},\nabla _i]\rho \big ) (\nabla ^{a_1}\cdots \nabla ^{a_k}\rho )\mu _g\\&\quad - \int _M \tau \nabla _i \big (\frac{u^i}{u^0} \big ) \big ( \nabla _{a_1}\cdots \nabla _{a_k}\rho \big ) (\nabla ^{a_1}\cdots \nabla ^{a_k}\rho )\mu _g \\&\quad - \int _M \tau \frac{u^i}{u^0} \big ( \nabla ^{a_1}\cdots \nabla ^{a_k}\rho \big ) ([\nabla _i ,\nabla _{a_1}\cdots \nabla _{a_k}]\rho )\mu _g \\&\quad - \int _M \tau \frac{u^i}{u^0} \big ( \nabla ^{a_1}\cdots \nabla ^{a_k}\rho \big ) (\nabla _{a_1}\cdots \nabla _{a_k} \nabla _i \rho )\mu _g.\end{aligned}\end{aligned}$$

Rearranging, we find that

$$\begin{aligned}\begin{aligned} \int _M&\tau \frac{u^i}{u^0} \big ( \nabla _{a_1}\cdots \nabla _{a_k}\nabla _i\rho \big ) (\nabla ^{a_1}\cdots \nabla ^{a_k}\rho )\mu _g \\&= \int _M \tau \frac{u^i}{u^0} \big ( [\nabla _{a_1}\cdots \nabla _{a_k},\nabla _i]\rho \big ) (\nabla ^{a_1}\cdots \nabla ^{a_k}\rho )\mu _g - \frac{1}{2} \int _M \tau \nabla _i \big (\frac{u^i}{u^0} \big )|\nabla ^k \rho |^2 \mu _g.\end{aligned}\end{aligned}$$

We see that we need to study two commutator error terms. Using (5.3), and noting that $\rho$ is a scalar, we see the first error term takes the form

$$\begin{aligned} |[\partial _T,\nabla _{a_1}\cdots \nabla _{a_k}]\rho | \lesssim \sum _{\begin{array}{c} |I|+|J|= k-1 \\ |J|\ge 1 \end{array}} |\nabla ^{I} (\partial _T \Gamma [g] ) ||\nabla ^{J} \rho |. \end{aligned}$$

Thus, using (5.2),

$$\begin{aligned} \Big | \int _M \big ( [\partial _T, \nabla _{a_1}\cdots \nabla _{a_k}]\rho \big ) (\nabla ^{a_1}\cdots \nabla ^{a_k}\rho )\mu _g\Big |&\lesssim \Vert \rho \Vert _{H^{k}}^2 \Vert \partial _T \Gamma [g]\Vert _{H^{N-2}} \\&\lesssim \varepsilon e^{-\lambda T}\mathcal {E}_k[\rho ](T). \end{aligned}$$

For the second commutator error term, we use (5.4) to find

$$\begin{aligned} |[\nabla _i,\nabla _{a_1}\cdots \nabla _{a_k}]\rho |\lesssim \sum _{\begin{array}{c} |I|+|J| = k-1\\ |J| \ge 1 \end{array}}| \nabla ^{I} \text {Riem} || \nabla ^{J} \rho |, \end{aligned}$$

and so we have

$$\begin{aligned}&\Big | \int _M \tau u^i \big ( [\nabla _{a_1}\cdots \nabla _{a_k},\nabla _i]\rho \big ) (\nabla ^{a_1}\cdots \nabla ^{a_k}\rho )\mu _g\Big | \\&\quad \lesssim |\tau | \Vert u \Vert _{H^2} \Vert \text {Riem} \Vert _{H^{k-2}}\Vert \rho \Vert _{H^{k}}^2 \lesssim \varepsilon e^{(-1+\mu )T} \mathcal {E}_k[\rho ](T). \end{aligned}$$

Putting this all together, and using Lemma 5.9, we obtain

$$\begin{aligned}\begin{aligned}|I_k|&\lesssim \Vert (u^0)^{-1} \rho \Vert _{H^k} \Vert \rho \Vert _{H^k} \Vert F_\rho \Vert _{H^k} + |\tau | \Vert \rho \Vert _{H^k} \Vert \nabla (\tfrac{u^i}{u^0}) \cdot \nabla _i \rho \Vert _{H^{k-1}} \\  &\quad + |\tau | \Vert \tfrac{u^i}{u^0}\Vert _{H^3} E_k[\rho ](T) +\varepsilon e^{\max \{-1+\mu ,-\lambda \} T} \mathcal {E}_k[\rho ](T). \\  &\lesssim \varepsilon e^{\max \{-1+\mu ,-\lambda \} T} \mathcal {E}_k[\rho ](T).\end{aligned} \end{aligned}$$

By summing over *k* we can conclude that

$$\begin{aligned} |\partial _T \mathcal {E}_{N-2}[\rho ](T)| \lesssim \varepsilon e^{\max \{-1+\mu ,-\lambda \} T}\mathcal {E}_{N-2}[\rho ](T). \end{aligned}$$

$$\square $$

### Definition 9.3

[$\mathcal {E}_k{[}u{]}$] Define the functionals

$$\begin{aligned}\begin{aligned} E_k[u](T)&:=\frac{1}{2} \int _M |\nabla ^k u(T, \cdot )|^2 \mu _g, \qquad \mathcal {E}_k[u](T)&:=\sum _{0\le \ell \le k} E_\ell [u](T).\end{aligned}\end{aligned}$$

### Proposition 9.4

(Evolution of $\mathcal {E}_{N-1}{[}u{]}$ fors spatial fluid velocity components) The following estimate holds:

$$\begin{aligned} \begin{aligned} |\partial _T \mathcal {E}_{N-1}[u](T)|&\lesssim \varepsilon e^{(-1+\mu )T} \mathcal {E}_{N-1}[u](T) + |\tau |^{-1} \Lambda (T) (\mathcal {E}_{N-1}[u](T))^{1/2}.\end{aligned} \end{aligned}$$

### Proof

Let $3 \le k \le N-1$. The proof is similar to the estimate for $\mathcal {E}_k[\rho ](T)$. As in the proof of Proposition 9.2, we obtain

$$\begin{aligned}\begin{aligned} \partial _T E_k[u](T)&\lesssim \Big ( \Vert \widehat{N}\Vert _{H^2} + \Vert X\Vert _{H^3} + \Vert \Sigma \Vert _{H^2} \Big ) E_k[u](T) + I_k',\end{aligned}\end{aligned}$$

where we have defined that

$$\begin{aligned} I'_k :=\int _M g_{ij}g^{a_1 b_1} \cdots g^{a_k b_k}\big ( \partial _T \nabla _{a_1}\cdots \nabla _{a_k}u^i\big ) (\nabla _{b_1}\cdots \nabla _{b_k}u^j)\mu _g. \end{aligned}$$

The integration by parts analysis on $I_k'$ follows unchanged to $I_k$. The main difference now arises in the commutator estimates since $u^a$ is a vector while $\rho$ is a scalar. For example, we now have an error term of the form

$$\begin{aligned} |[\partial _T,\nabla _{a_1}\cdots \nabla _{a_k}]u^i | \lesssim \sum _{|I|+|J|= k-1} |\nabla ^{I} (\partial _T \Gamma [g] )|| \nabla ^{J} u^i|. \end{aligned}$$

Thus, using (5.2),

$$\begin{aligned}&\Bigg | \int _M \big ([\partial _T, \nabla _{a_1}\cdots \nabla _{a_k}]u^i \big ) (\nabla ^{a_1}\cdots \nabla ^{a_k}u_i )\mu _g\Bigg | \\&\quad \lesssim \Vert u^a\Vert _{H^{k}}^2 \Vert \partial _T \Gamma (g)\Vert _{H^{N-2}} \lesssim \varepsilon e^{-\lambda T} \mathcal {E}_k[u^a](T) . \end{aligned}$$

Similarly, the second commutator error term looks like

$$\begin{aligned} |[\nabla _i,\nabla _{a_1}\cdots \nabla _{a_k}]u^i | \lesssim \sum _{|I|+|J| = k-1} |\nabla ^{I} \text {Riem} || \nabla ^{J} u^i|, \end{aligned}$$

and so we have

$$\begin{aligned}&\Bigg | \int _M \tau u^a \big ( [\nabla _{a_1}\cdots \nabla _{a_k},\nabla _a]u^i \big ) (\nabla ^{a_1}\cdots \nabla ^{a_k}u_i )\mu _g\Bigg | \\&\quad \lesssim |\tau |\Vert u\Vert _{H^2} \Vert \text {Riem} \Vert _{H^{k-1}}\Vert u\Vert _{H^{k}}^2 \lesssim \varepsilon e^{(-1+\mu ) T}\mathcal {E}_k[u](T) . \end{aligned}$$

Recalling from (2.7d) the equation of motion

$$\begin{aligned} \partial _Tu^j = \tau (u^0)^{-1} u^i \nabla _i u^j + \tau ^{-1}u^0 N\nabla ^j N+(u^0)^{-1}F_{u^j}, \end{aligned}$$

we obtain

$$\begin{aligned}\begin{aligned} |I'_k|&\lesssim |\tau | \Vert (u^0)^{-1}u^a \Vert _{H^3}E_k[u](T) + |\tau | \Vert u \Vert _{H^k} \Vert \nabla (\tfrac{u^i}{u^0}) \cdot \nabla _i u^a \Vert _{H^{k-1}} \\  &\quad + |\tau |^{-1} \Vert \widehat{N}\Vert _{H^{k+1}} \Vert u\Vert _{H^k} + \Vert (u^0)^{-1}F_{u^j} \Vert _{H^k}\Vert u\Vert _{H^k} \\  &\quad +\varepsilon e^{\max \{-1+\mu ,-\lambda \} T} E_k[u](T) \\  &\lesssim \varepsilon e^{(-1+\mu )T} E_k[u](T) + \Big ( |\tau |^{-1} \Vert \widehat{N}\Vert _{H^{k+1}} + \Vert F_{u^j}\Vert _{H^k} \Big )E_k[u](T)^{1/2}.\end{aligned}\end{aligned}$$

The conclusion then follows by summing over *k* and using the source estimates for $F_{u^j}$ given in Lemma 5.9. $\square$

## Proof of Theorem 1.2

In this final section we bring together our main estimates to conclude the bootstrap argument. One standard, yet technical, aspect of the argument is deferred to Appendix 11.

### Proof

Smallness of the initial data guarantees the existence of a constant $C_0>0$ such that at $T=T_0$

$$\begin{aligned} \begin{aligned}&\Vert \widehat{N}\Vert _{H^N} + \Vert X\Vert _{H^N} + \Vert \partial _T N \Vert _{H^{N-1}} + \Vert \partial _T X \Vert _{H^{N-1}} \\&\qquad + ({E^g}_{N-1}(T_0))^{1/2} + (\mathcal {E}^g_{\partial _{\textbf{u}},N-1}(T_0))^{1/2} + \mathcal {E}_{N-2}[\rho ](T_0)^{1/2} +\mathcal {E}_{N-1}[u^a](T_0)^{1/2}\\&\quad \le C_0 \varepsilon .\end{aligned} \end{aligned}$$

Proposition 9.2, together with Grönwall’s inequality, implies

$$\begin{aligned} \Vert \rho \Vert _{H^{N-2}} \le \mathcal {E}_{N-2}[\rho ](T_0)^{1/2} \exp \Big ( \int _{T_0}^T C\varepsilon e^{\max \{-1+\mu ,-\lambda \} s} \text {d}s \Big )^{1/2} \le C_0 \varepsilon \exp (C' \varepsilon ). \end{aligned}$$

Thus, by Lemma 7.1, and shrinking $\varepsilon$ as needed,

$$\begin{aligned}\begin{aligned} \Vert \widehat{N}\Vert _{H^{N}} + \Vert X \Vert _{H^N}&\le |\tau | C\Vert \rho \Vert _{H^{N-2}} + \varepsilon ^2 Ce^{\max \{-2\lambda , -2+\mu \}T} \le C C_0 \varepsilon e^{-T}.\end{aligned} \end{aligned}$$

Turning next to Lemma 7.2 we find that

$$\begin{aligned}&\Vert \partial _T N \Vert _{H^{N-1}} + \Vert \partial _T X \Vert _{H^{N-1}}\\&\quad \le C \Vert \widehat{N}\Vert _{H^N}+C \Vert X\Vert _{H^N} + |\tau |C \Vert \rho \Vert _{H^{N-2}} + \varepsilon ^2 C e^{\max \{-2\lambda , -2+\mu \}T}\\&\quad \le C_0 \varepsilon e^{-T}. \end{aligned}$$

Next, we divide the estimate in Proposition 9.4 by the square root of the energy, to obtain

$$\begin{aligned} |\partial _T \mathcal {E}_{N-1}[u](T)^{1/2}| \le C \varepsilon e^{(-1+\mu )T} \mathcal {E}_{N-1}[u](T)^{1/2} + C C_0 \varepsilon . \end{aligned}$$

An application of Grönwall’s inequality then produces

$$\begin{aligned} \begin{aligned} \Vert u\Vert _{H^{N-1}}&\le \Big (\mathcal {E}_{N-1}[u](T_0)^{1/2} + \int _{T_0}^T C C_0\varepsilon \text {d}s \Big ) \exp \Big (\int _{T_0}^TC \varepsilon e^{(-1+\mu )s}\text {d}s\Big ) \\  &\le C\big (C_0 \varepsilon + C_0\varepsilon (T-T_0) \big ).\end{aligned} \end{aligned}$$

Up to possibly redefining $T_0$, see the discussion around (11.1) in Appendix 11, these inequalities now imply

$$\begin{aligned} \Lambda (T) \le C C_0 \varepsilon e^{-T}. \end{aligned}$$

Finally adding together the results of Proposition 8.1 and Theorem 8.3 and using the above improved estimates, we find

$$\begin{aligned}\begin{aligned} \partial _T E^g_{tot}&\le -2\alpha E^g_{tot}+ CC_0 \varepsilon e^{-1+\mu } E^g_{tot}+ CC_0 \varepsilon e^{-T} (E^g_{tot})^{1/2} + C(E^g_{tot})^{3/2} + C\Lambda (T)^2.\end{aligned} \end{aligned}$$

Thus, by the bootstrap argument presented in Appendix 11, we obtain

$$\begin{aligned} {E^g_{\partial _{\textbf{u}},N-1}}+ E^g_{N-1} \le \frac{1}{2} C_1^2 \varepsilon ^2 e^{-2\lambda T}, \end{aligned}$$

where $C_1 \gg C_0$. Finally, as a consequence of Corollary 6.4,

$$\begin{aligned}\begin{aligned} \Vert g-\gamma \Vert _{H^N}^2 +\Vert \Sigma \Vert _{H^{N-1}}^2&\lesssim {E^g_{\partial _{\textbf{u}},N-1}}+ E^g_{N-1} + \Lambda (T)^2 \le \frac{3}{4} C_1^2 \varepsilon ^2 e^{-2\lambda T}.\end{aligned} \end{aligned}$$

It remains to show that ${\tilde{\rho }}\ge 0$ given that initially ${\tilde{\rho }}_0\ge 0$. We recall an argument given in [15]. We can rewrite the evolution equation for the energy density, to obtain

$$\begin{aligned} \tilde{u}^{\alpha }{\bar{\nabla }}_{\alpha }{\tilde{\rho }}+{\tilde{\rho }}{\bar{\nabla }}_\alpha \tilde{u}^{\alpha }=0. \end{aligned}$$

Moreover, the spacetime is ruled by the geodesics tangent to $\tilde{u}^\mu$. Along the geodesics positivity of ${\tilde{\rho }}$ or ${\tilde{\rho }}=0$ is conserved due to the equation above and the regularity of $\tilde{u}^\mu$. Hence ${\tilde{\rho }}\ge 0$ on the future development holds. $\square$

## Data Availability

Data sharing is not applicable to this article as no datasets were generated or analysed during the current study.

## Appendix A. Background geometry

In this appendix we derive the background solutions given in Remark 2.5. On the Milne background the ADM variables induced on a $t_c=const$ slice are

$$\begin{aligned} (\tilde{g}_{ab}, \tilde{k}_{ab}, \tilde{N}, \tilde{X}^a)|_B = (\tfrac{t_c^2}{9} \gamma _{ab}, -\tfrac{1}{t_c}\gamma _{ab}, 1, 0) , \quad \tau = \tilde{g}^{ab}\tilde{k}_{ab} = -\tfrac{3}{t_c}. \end{aligned}$$

Condition (2.2) implies $(\tilde{u}^{t_c})^2 = 1+\tfrac{t_c^2}{9}\gamma (\tilde{u}, \tilde{u}).$ The fluid equations of motion in cosmological coordinates $(t_c, x^i)$ read

$$\begin{aligned}\begin{aligned} \tilde{u}^{t_c}\partial _{t_c} \ln {\tilde{\rho }}+\tilde{u}^i \partial _i \ln {\tilde{\rho }}+3 \partial _{t_c} \tilde{u}^{0}+ \partial _i \tilde{u}^i+3t_c^{-1}\tilde{u}^{t_c}+\Gamma ^i_{ij}[\gamma ]\tilde{u}^j&=0, \\ \tilde{u}^{0}\partial _{t_c}\tilde{u}^{0} +\tilde{u}^i\partial _i \tilde{u}^{0}-\tfrac{1}{t_c}\gamma (\tilde{u}, \tilde{u})&=0,\\ \tilde{u}^{t_c}\partial _{t_c}\tilde{u}^{i} +\tilde{u}^i\partial _i \tilde{u}^{i}-2\tfrac{t_c}{9}\tilde{u}^{t_c}\tilde{u}^i+ \Gamma ^i_{jk}[\gamma ]\tilde{u}^j\tilde{u}^k&=0. \end{aligned} \end{aligned}$$

Picking $\tilde{u}^{t_c}=1, \tilde{u}^i=0$ we have $\partial _{t_c} \ln {\tilde{\rho }}+ 3t_c^{-1}= 0$. Thus on the background the fluid solution is

$$\begin{aligned} (\tilde{u}^{t_c}, \tilde{u}^i, {\tilde{\rho }})|_B = (1, 0, {\tilde{\rho }}_0 t_c^{-3}) \end{aligned}$$

where ${\tilde{\rho }}_0>0$ is a constant. By rescaling the variables as in Definition 2.2 we arrive at Remark 2.5.

## Appendix B. Matter Variables

In this appendix we discuss the rescaled components of the energy momentum tensor given in Definition 2.6. To see the origin of the various terms, we follow [1] and introduce the following notation for the matter variables

$$\begin{aligned} \begin{aligned} \tilde{E}&:=\tilde{T}^{\mu \nu }n_\mu n_\nu =\tilde{T}^{00}\tilde{N}^2, \quad&{\tilde{\jmath }}^a&:=-\tilde{T}^{\mu \nu }\perp ^a _\mu n_\nu , \\ \tilde{\eta }&:=\tilde{E}+\tilde{g}^{ab}\tilde{T}_{ab}, \quad&\tilde{S}_{ab}&:=\tilde{T}_{ab}-\frac{1}{2} \text {Tr}_{\bar{g}} \tilde{T}\cdot \tilde{g}_{ab}, \quad \tilde{T}^{ab} :={\tilde{\rho }}\tilde{u}^a\tilde{u}^b \bar{g}^{ab}.\end{aligned} \end{aligned}$$

Using (2.1) we find

$$\begin{aligned} \begin{aligned} \text {Tr}_{\bar{g}} \tilde{T}&= \bar{g}^{\mu \nu }\tilde{T}_{\mu \nu }=-{\tilde{\rho }}, \\ \tilde{T}_{ab}&= \bar{g}_{a\mu } \bar{g}_{b\nu } \tilde{T}^{\mu \nu } = {\tilde{\rho }}( \tilde{X}_a \tilde{u}^0 + \tilde{g}_{ai}\tilde{u}^i)(\tilde{X}_b \tilde{u}^0 + \tilde{g}_{bj}\tilde{u}^j ), \\ \tilde{g}^{ab}\tilde{T}_{ab}&= {\tilde{\rho }}(\tilde{X}^a\tilde{X}_a (\tilde{u}^0)^2 + 2 \tilde{X}_b \tilde{u}^b \tilde{u}^0 + \tilde{g}_{ab}\tilde{u}^a\tilde{u}^b ).\end{aligned} \end{aligned}$$

A calculation then yields

$$\begin{aligned} \begin{aligned} \tilde{E}&=|\tau |^3 \rho (u^0)^2 N^2 , \qquad {\tilde{\jmath }}^a =|\tau |^5 \rho u^0 u^a N , \\ \tilde{\eta }&= |\tau |^3 \rho (u^0)^2 N^2 + |\tau |^3 \rho \Big (X_a X^a (u^0)^2 + 2\tau X_b u^b u^0 + \tau ^2 g_{ab} u^a u^b \Big ), \\ \tilde{S}_{ab}&= |\tau | \rho \Big ( X_a X_b (u^0)^2 + \tau u^0 u^c X_b g_{ac} + \tau u^0 u^c X_a g_{bc} + \tau ^2 g_{ac}g_{bd}u^cu^d\Big ) +|\tau | \frac{\rho }{2}g_{ab} , \\ \tilde{T}^{ab}&= |\tau |^7\rho u^au^b.\end{aligned} \end{aligned}$$

Finally, we introduce the following rescaled variables

$$\begin{aligned}\begin{aligned} E&:=|\tau |^{-3} \tilde{E} , \quad&\jmath ^a&:=|\tau |^{-5} \tilde{\jmath }^a, \\ \eta&:=|\tau |^{-3}\tilde{\eta }, \quad&S_{ab}&:=|\tau |^{-1} \tilde{S}_{ab}, \quad \underline{T}^{ab}&:=|\tau |^{-7}\tilde{T}^{ab},\end{aligned} \end{aligned}$$

which yield the expressions given in Definition 2.6.

## Appendix C. Bootstrap

In this appendix we detail how the bootstrap assumption for $E^g_{tot}(=f)$ is closed. Let $\lambda <1$ and $\mu \ll 1$ be fixed constants. Let $f:[T_0,T_*)\rightarrow [0,\infty )$ be a continuous function, where $T_0 \le T_* \le \infty$. Suppose $f(T_0)\le C_0^2 \varepsilon ^2$. Suppose also, that for each $T_0 \le T<T_*$ the assumption $f(T) \le C_1^2 \varepsilon ^2 e^{-2\lambda T}$ allows us to show

$$\begin{aligned} \partial _T f \le - 2\alpha f + C C_0 \varepsilon e^{(-1+\mu )T} f + C C_0 \varepsilon e^{-T} f^{1/2} + C f^{3/2} + C C_{0}^2\varepsilon ^2 e^{-2T}. \end{aligned}$$

Since $\alpha \in [1-\delta _\alpha , 1]$ we pick a $\zeta$ such that $\lambda< \zeta <1$ and $\lambda< \alpha \zeta <\tfrac{1}{2}(1+\lambda )$.^1^ Define also $\beta >0$ to be the difference $\beta :=\alpha \zeta - \lambda$. We then have

$$\begin{aligned}\begin{aligned} \partial _T (e^{2\alpha \zeta T} f)&\le -2\alpha (1-\zeta ) e^{2\alpha \zeta T}f + C C_0 \varepsilon e^{(-1+\mu + 2\alpha \zeta )T} f\\&\quad + C C_0 \varepsilon e^{(-1+ 2\alpha \zeta ) T} f^{1/2} + C f^{3/2} e^{2\alpha \zeta T} + C C_{0}^2\varepsilon ^2 e^{(-2+ 2\alpha \zeta )T} \\&\le -\Big ( 2\alpha (1-\zeta ) - C C_0 \varepsilon e^{(-1+\mu )T} - C f^{1/2}\Big ) e^{2\alpha \zeta T}f\\&\quad + C C_0 \varepsilon e^{(-1+ 2\alpha \zeta ) T} f^{1/2} + C C_{0}^2\varepsilon ^2.\end{aligned} \end{aligned}$$

Substituting in the bootstrap assumption, and choosing $\varepsilon$ sufficiently small:

$$\begin{aligned} \begin{aligned} \partial _T (e^{2\alpha \zeta T} f)&\le -\Big ( 2\alpha (1-\zeta ) - C C_0 \varepsilon - C C_1 \varepsilon \Big ) e^{2\alpha \zeta T}f \\&\quad + C C_0 \varepsilon e^{(-1+ 2\alpha \zeta ) T} f^{1/2} + C C_{0}^2\varepsilon ^2 \\&\le C C_1 C_0 \varepsilon ^2 e^{(-1+ 2\alpha \zeta -\lambda ) T} + C C_{0}^2\varepsilon ^2 \\&\le C C_1 C_0 \varepsilon ^2.\end{aligned} \end{aligned}$$

Thus, by Grönwall’s inequality,

$$\begin{aligned} \begin{aligned} e^{2\alpha \zeta T} f&\le C_0^2 \varepsilon ^2 + \int _{T_0}^T C C_1 C_0 \varepsilon ^2 \text{ d }s \\ \Rightarrow f&\le \big (C C_0^2 \varepsilon ^2 + C C_0 C_1 \varepsilon ^2(T-T_0) \big )e^{-2\beta T}e^{-2\lambda T}.\end{aligned} \end{aligned}$$

Let $T_0^*$ be such that, for all $T\ge T_0^*$,

11.1 $$\begin{aligned} \big (C C_0^2 + C C_0 C_1 (T-T_0) \big )e^{-2\beta T} < \frac{1}{2} C_1^2. \end{aligned}$$

We then *a fortiori* choose $T_0\ge T_0^*$. Thus we have shown that, for all $T'\in [T_0,T_*)$, if $f(T) \le C_1^2 \varepsilon ^2 e^{-2\lambda T}$ for each $T \in [T_0, T']$, that $f(T) \le \frac{1}{2} C_1^2 \varepsilon ^2 e^{-2\lambda T}$. Therefore $f(T) \le \frac{1}{2} C_1^2 \varepsilon ^2 e^{-2\lambda T}$ for all $T \in [T_0, T_*)$.
